# A Concise Synthesis of Three Branches Derived from Polysaccharide RN1 and Anti-Pancreatic Cancer Activity Study

**DOI:** 10.3390/polym9100536

**Published:** 2017-10-21

**Authors:** Deqin Cai, Yanli Yao, Yubo Tang, Zheng Wang, Wei Shi, Wei Huang, Kan Ding

**Affiliations:** 1 University of Chinese Academy of Sciences, No.19A Yuquan Road, Beijing 100049, China; deqincai2012@163.com (D.C.); yaoyanli1016@163.com (Y.Y.); hsw201204@163.com (W.S.); huangwei@simm.ac.cn (W.H.); 2 Glycochemistry and Glycobiology Lab, Shanghai Institute of Materia Medica, Chinese Academy of Sciences, 555 Zuchongzhi Road, Shanghai 201203, China; wzheng92@mail.ustc.edu.cn; 3 CAS Key Laboratory of Receptor Research, CAS Center for Excellence in Molecular Cell Science, Shanghai Institute of Materia Medica, Chinese Academy of Sciences, 555 Zuchongzhi Road, Pudong, Shanghai 201203, China; yubotang1107@163.com; 4 Key Laboratory of Structure-Based Drug Design and Discovery, Ministry of Education, Shenyang Pharmaceutical University, Shenyang 110016, China; 5 Nano Science and Technology Institute, University of Science and Technology of China, 96 Jin Zhai Road, Hefei 230026, China

**Keywords:** arabinogalactan, pancreatic cancer, gemcitabine resistance, carbohydrate chemistry

## Abstract

RN1, a polysaccharide from flowers of *Panax pseudo-ginsieng* Wall. Var. *notoginseng* (Burkill) Hoo & Tseng, is a potential multi-targeting drug candidate for pancreatic cancer treatment. However, the active targeting domain of RN1 is still unknown. Herein, three RN1 derived branches were synthesized via [3+2] or [2+2] strategies, efficiently. Two pentasaccharides, **18** and **27**, showed similar inhibition effect on pancreatic cancer BxPC-3 cells to that of RN1 at same concentration. Interestingly, tetrasaccharide **21** potently inhibited gemcitabineresistant cell line Panc-1 at high concentration. These suggest that the branches of RN1 might be the active targeting domain and tetrasaccharide **21** might be a potential leading compound for pancreatic cancer with gemcitabine resistance.

## 1. Introduction

Pancreatic cancer is a cancer with high mortality, which is close to incidence [1]. Gemcitabine is currently one of the major treatments for pancreatic cancers, apart from operation in clinical. However, the response rate of gemcitabine had been decreased year by year [2]. Despite new adjuvant gemcitabine, gemcitabine combination therapy, and new active compounds were reported [3,4,5,6,7], the low survival rate of patients with pancreatic cancer never changed essentially. Carbohydrate-based drug possess the specific advantage in cytotoxicity because of the consisting of saccharide residues. Sugar drugs usually function in a specific mechanism and offer new opportunities for drug discovery [8].

**Figure 1:**
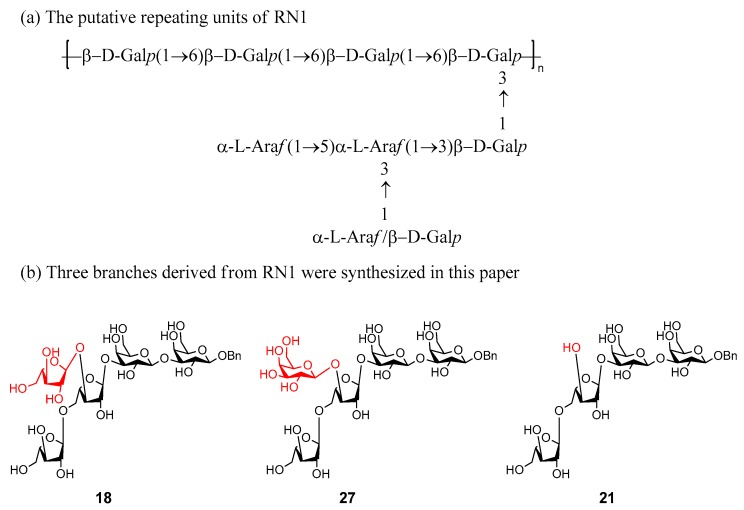
(**a**) The putative repeating units of RN1, a Gal-β-1,6-Gal tetrasaccharide backbone carrying one of the two possible arabinogalactan branches; (**b**) Three branches derived from RN1 were synthesized in this paper. They share same tetrasaccharide **21** skeleton.

Recently, an arabinogalactan polysaccharide RN1, a multi-targeting polysaccharide from flowers of *Panax notoginseng* that has anti-pancreatic cancer activity, was reported in this lab [9,10]. The complexity of the structure of polysaccharides prevent their investigation of polysaccharide, including drug discovery. The possible unknown impurities in natural extract polysaccharides may cause adverse effects. In addition, quality control of polysaccharide-based drugs is always a challenge. So, discovering the active domain of polysaccharide RN1 and synthesis the corresponding structure and well-defined sugar chains are necessary. As shown in Figure 1a, the putative repeating unit of RN1 is a Gal-β-1,6-Gal tetrasaccharide backbone carrying one of the two possible arabinogalactan branches [9]. In this paper, we chemically synthesized two branches of RN1 to explore the role of branches in anti-pancreatic cancer. Besides, a common tetrasaccharide **21** of these two branches was synthesized to compare with the other two branch compounds (**18**, **27** in Figure 1b). Certainly, it’s better to protect the reducing end of these three oligosaccharides with benzyl than free hydroxyl, because of the possible π–π interacting with targeting proteins. There are also other choices in the reducing end, but the structure-activity relationship would be investigated in the future.

## 2. Materials and Method

### 2.1. General Experimental Procedures

All chemical reagents (AR) and solvents (AR) were purchased from Sinopharm Chemical Reagent Co. (Shanghai, China) and used without further purification. Reactions were monitored by TLC (Thin layer chromatography) on glass Silica Gel HSGF254 (Sinopharm Chemical Reagent Co., Shanghai, China) plates with UV 254 nm detection. TLC staining for carbohydrate samples was performed by dipping the plates into 10% H_2_SO_4_ in ethanol and drying with a heat gun. Anhydrous dichloromethane (DCM) was freshly distilled from calcium hydride under nitrogen prior to use. Molecular sieves 4Å powder was purchase from Sigma-Aldrich Co. LLC. (St. Louis, MO, USA), and was activated by heating at 200 °C in vacuum for 2 h. Nuclear magnetic resonance (NMR) spectra were measured on a Varian-MERCURY Plus (400 MHz) (Varian, Inc., Palo Alto, CA, USA) and Bruker AVANCE III (500 MHz) (Bruker Corporation, Billerica, MA, USA). The chemical shifts were assigned in ppm and the coupling constants in Hz. ESI-HRMS spectra were measured on an Agilent 6230 LC-TOF MS spectrometer (Agilent Technologies Inc., Santa Clara, CA, USA).

Detail experimental operations and the corresponding data are available in the supplementary materials. The structures of each new compound were shown in Figures S2–S15 in Supplementary Materials. The pictorial form of NMR data of each new compound were shown in Figures S16–S74. These figures were arranged according to the numeric name of new compounds from small to large. 

### 2.2. Cells and Materials

All cells were obtained from the Cell Bank in the Type Culture Collection Center of the Chinese Academy of Sciences, Shanghai, China. The human pancreatic ductal adenocarcinoma cell lines Panc-1, BxPC-3 and the normal hepatic cell line LO2 were cultured in RPMI-1640 medium supplemented with 10% fetal bovine serum and 1% penicillin/streptomycin. All reagents used for the cell culture studies were purchased from Gibco (Pittsburgh, PA, USA). All cells were maintained at 37 °C, in a humidified atmosphere, with 5% CO_2_ in air, and subcultured every 3–5 days. MTT, 3-(4,5-dimethylthiazol-2-yl)-2,5-diphenyltetrazolium bromide, used to determine cell viability, was obtained from Sigma-Aldrich (Louis, MO, USA).

### 2.3. Cell Viability Assessment

Cell viability was determined using MTT assays. Briefly, cancer cells and normal liver cells were treated with certain concentrations of compounds (0–500 μM) for 72 h. MTT (20 μL, 0.5 mg/mL) was added and incubated for another 4 h, and then the supernatant was removed and the dye crystals were dissolved in 200 μL DMSO. Absorbance was measured at 490 nm using a microplate reader (Bio-Rad, Hercules, CA, USA).

## 3. Result and Discussion

### 3.1. Retrosynthetic Analysis

**Figure 2:**
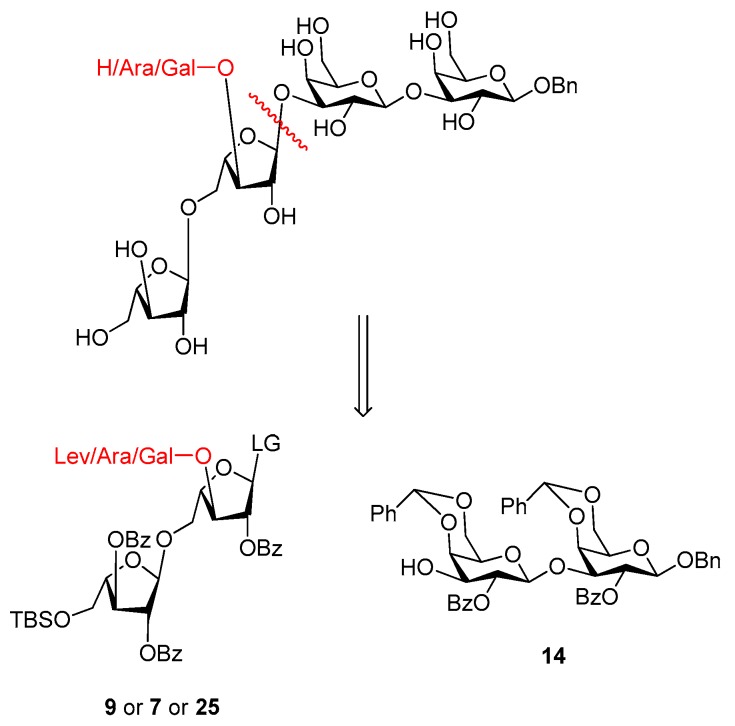
Retrosynthetic analysis of three branches derived from RN1. Bz = benzoyl, Bn = benzyl, Lev = levulinoyl, TBS = *t*-butyldimethylsilyl.

As shown in Figure 2, these three oligosaccharides were synthesized via [3+2] or [2+2] strategies. Trisaccharides or disaccharide donors were used for the corresponding target oligosaccharides. Gal-Gal disaccharide **14** was shared as common acceptor. With the protection of 4,6-O-benzylidene, 3-OH will be more active, facilitate further glycosylation. Donor **7** and donor **9** were reacted with acceptor **14** through directly activating *p*-tolyl under NIS/TMSOTf (N-Iodosuccinimide/Trimethylsilyl Triflate). But, donor **25** was a trichloroacetimidate, for the failure of the synthesis of its *p*-tolyl structure. 

### 3.2. Synthesis of Donors ***7***, ***9***, and Acceptor ***14***

**Scheme 1:**
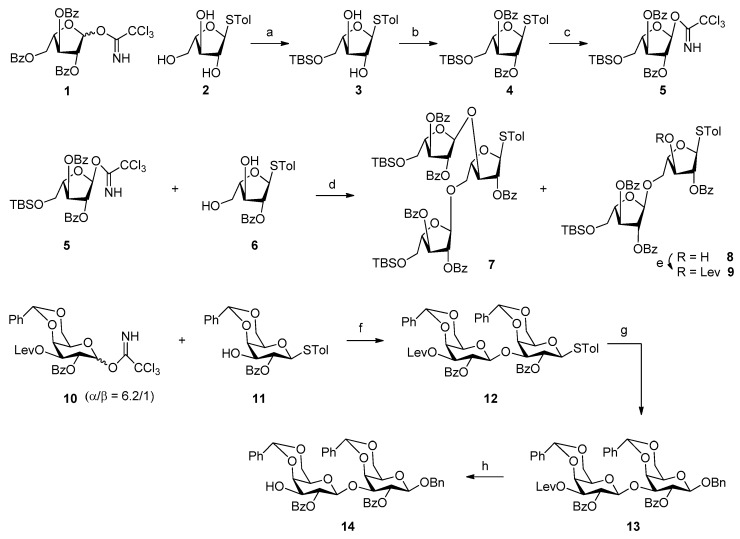
Preparation of donors **7** and **9** and acceptor **14**. (**a**) TBSCl (*t*-Butyldimethylsilyl chloride), imidazole, DCM (Dichloromethane, 66%); (**b**) BzCl, pyridine, ice-bath (84%); (**c**) (*i*) NIS, TFA (Trifluoracetic acid), DCM, ice-bath; (*ii*) CCl_3_CN, DBU (1,5-Diaza(5,4,0)undec-5-ene), DCM (56% in two steps); (**d**) TMSOTf, DCM, ice-bath (18% for **7**, 54% for **8,** or 74% for **8** only); (**e**) EDCI, DMAP, LevOH, DCM (87%); (**f**) TMSOTf, DCM, −78 °C (62%); (**g**) NIS, TMSOTf, BnOH (benzyl alcohol), ice-bath; (**h**) N_2_H_4_–H_2_O, AcOH, MeOH/DCM (72% in two steps).

To prepare the l-arabinose trisaccharide donor for petasaccharide **18**, full Bz protected l-Ara donor **1** (Scheme 1) and the known acceptor **6** were used at the beginning according to the published d-arabinose trisaccharide by employing a full Bz protected d-Ara donor [11]. Interestingly, donor **1** cannot work in the glycosylation, but results in a thiolphenol byproduct. A similar result had also been reported in the synthesis of glucans [12,13]. According to the previous work [14], Bz is a disarmed protect group, which reduces the reactivity of the donor. Additional two Bz lead to the disparity of the reactivity of donor **1** and acceptor **6**, so then *p*-tolylthiol was transferred. In the next step, a more reactive l-Ara, donor **5**, was designed. The 5-O-Bz of donor **1** was replaced by TBS (*t*-Butyldimethylsilyl). There were three reasons to choose TBS: (1) TBS can selectively protect primary hydroxyl in high yield; (2) TBS is a good armed protect group, even better than benzyl [15]; (3) TBS can be removed under acidic condition together with benzylidene of Gal to simplify deprotection. The synthesis of donor **5** start from the known compound **2** [16], over four steps. Controlling the ratio of donor **5** and acceptor **6** in glycosylation may lead to different yields of trisaccharide **7** and disaccharide **8**. The free hydroxyl of disaccharide **8** was then Levprotected to afford disaccharide donor **9** under EDCI/DMAP/LevOH (1-Ethyl-3-(3-dimethylaminopropyl)-carbodiimide hydrochloride/4-Dimethylaminopyridine/Levulinic acid). 

To prepare disaccharide acceptor **14**, donor **10** and the known acceptor **11** [17] were reacted under TMSOTf at −78 °C to afford disaccharide **12**. Low temperature is necessary in this reaction. Activating of **12** to couple with benzyl alcohol, and the resulting mixture continued to deprotect Lev without further purification, to afford important acceptor **14**.

### 3.3. Exploration of the Synthesis of the Gal-Containing Trisaccharide Donor

**Scheme 2:**
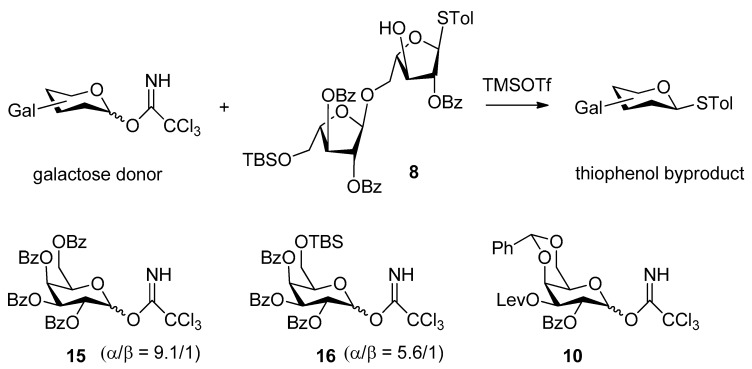
Exploration of the synthesis of Gal containing trisaccharide donor for **27**. Because of the instability of *p*-Tolyl, thiolphenol byproduct was the major product (>50%), instead of the target trisaccharide.

It is difficult to synthesize the remaining Gal-containing trisaccharide donor. Similar to trisaccharide **7**, disaccharide **8** was used as acceptor, along with two easily prepared and one already available Gal donors—**15**, **16** and **10**—but none of them can afford the target trisaccharide in a major product, instead a thiolphenol byproduct (Scheme 2). Perhaps more active Gal donors would work, such as *p*-Tol 2-O-Benzoyl-3,4,6-O-tribenzylthiogalactoside, but access to those donors need additional several steps and extra deprotection of benzyl will be needed, which will make the synthetic route lower efficient. 

### 3.4. Synthesis of Target Compound ***18***, ***21***

**Scheme 3:**
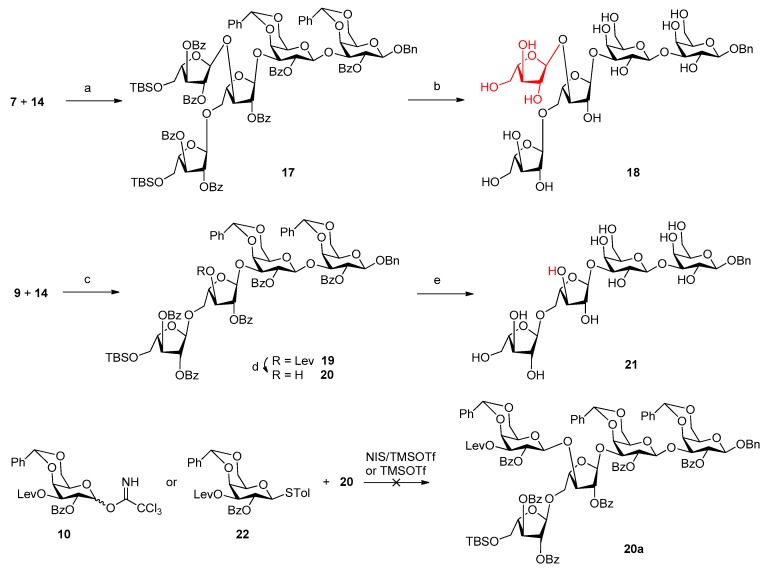
Synthesis of target pentasaccharide **18** and tetrasaccharide **21**, and exploration of the synthesis of pentasaccharide **20a**. The free hydroxyl of **20** is rather low activity and can hardly react with donors **10** and **22**. (**a**) NIS, TMSOTf, DCM, ice-bath (71%); (**b**) (*i*) 80% acetic acid, 80 °C; (*ii*) NaOMe, MeOH (79% in two steps); (**c**) NIS, TMSOTf, DCM, ice-bath; (**d**) N_2_H_4_-H_2_O, AcOH, MeOH/DCM (47% in two steps); (**e**) (*i*) 80% acetic acid, 80 °C; (*ii*) NaOMe, MeOH (56% in two steps).

With donors **7**, **9** and acceptor **14** in hand, pentasaccharide **17** and tetrasaccharide **19** were prepared via thiolphenol glycosidic method under NIS/TMSOTf. The resulting reaction mixture of **19** then removed Lev to afford **20** without further purification. The deprotection of **17** and **20** was similar. **17** or **20** was treated with 80% acetic aqueous solution at 80 °C to remove TBS and benzylidene, and the acyl groups were removed in methanol at pH about **10**, the resulting mixture was purified by “P2” column to afford pentasaccharide **18** and tetrasaccharide **21**, respectively. Tetrasaccharide **20** was also tried as acceptor for pentasaccharide **20a**. Probably, for the steric hindrance, neither donor **10** nor donor **22** work, not to mention lower active donors **15** and **16**.So, the challenging glycosidic bond should be formed with a disaccharide acceptor (Scheme 3).

### 3.5. Synthesis of the Target and Challenging ***27***

**Scheme 4:**
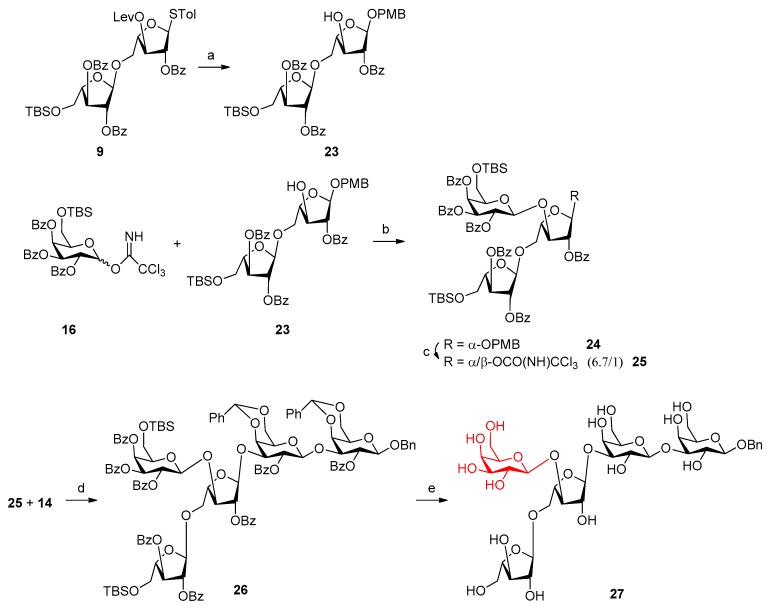
Synthesis of the target pentasaccharide **27**. (**a**) (*i*) NIS, TMSOTf, DCM, ice-bath; (*ii*) N_2_H_4_-H_2_O, AcOH, MeOH/DCM (75% in two steps); (**b**) TMSOTf, DCM, ice-bath (65%); (**c**) (*i*) DDQ, DCM/water; (*ii*) CCl_3_CN, DBU, DCM (66% in two steps); (**d**) TMSOTf, DCM, ice-bath (62%); (**e**) (*i*) 80% acetic acid, 80 °C; (*ii*) NaOMe, MeOH (56% in two steps).

As shown in Scheme 4, in order to avoid the thiophenol byproduct, disaccharide **9** was coupled with *p*-methoxybenzyl alcohol and subsequently to remove Lev without further purification to afford a new disaccharide, acceptor **23**. Compared to donor **15**, donor **16** was more active and obtained much higher yields to afford **24**. The PMB (4-Methoxybenzylchloride) of **24** was removed by DDQ (1,2-Dichloro-4,5-dicyanobenzoquinone) and then converted into trichloroacetimidate to afford trisaccharide donor **25**. Then, **25** was successfully coupling with common disaccharide acceptor **14** to afford **26** in good yield, and through a similar deprotection to **17** and **20** to afford the remaining target pentasaccharide **27**.

### 3.6. Anti-Pancreatic Cancer Activity Test 

**Figure 3:**
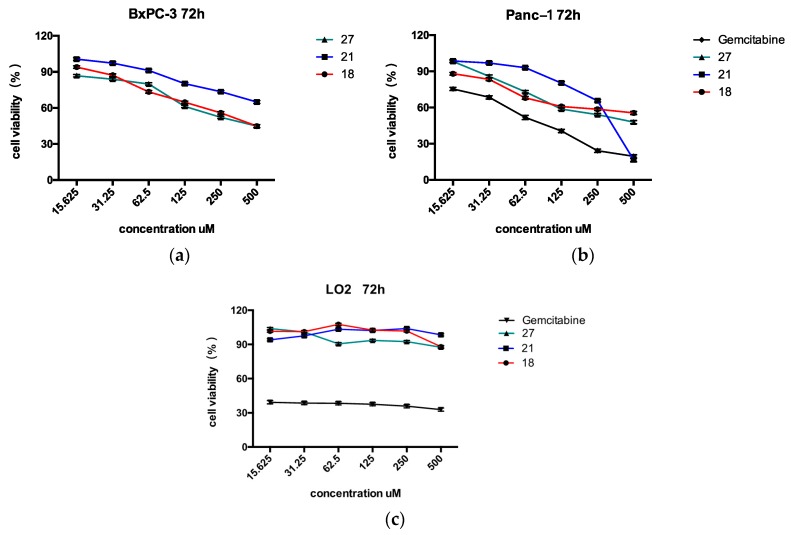
Anti-pancreatic cancer activity and cytotoxicity test of oligosaccharides **18**, **21**, and **27**. BxPC-3 (**a**), PANC-1 cells (**b**) were treated with compounds **18**, **21**, **27**, and gemcitabine (as a positive control) at concentrations of 15.625, 31.24, 62.5, 125, 250, and 500 μM for 72 h followed by MTT test. LO2 cells (**c**) were treated with compounds **18**, **21**, **27** at concentrations of 15.625, 31.24, 62.5, 125, 250, and 500 μM for 72 h followed by MTT test.

Similar to RN1, these three saccharides were then tested in pancreatic cancer cell line BxPC-3. As shown in Figure 3a, pentasaccharides **18** and **27** exhibited about 55% inhibition effect on BxPC-3 at around concentrations of 500 μM (414 μg/mL for **18** and 429 μg/mL for **27**), which is similar to the published RN1 polysaccharide at same mass concentration. But, activity of tetrasaccharide **21** is not well than that of the pentasaccharides in BxPC-3. Meanwhile, these three oligosaccharides were also tested in the gemcitabineresistant pancreatic cancer cell line Panc-1. Surprisingly, tetrasaccharide **21** exhibit nearly 85% inhibition at 500 μM, which is similar to gemcitabine (Figure 3b). In Figure 3b, tetrasaccharide **21** exhibited different linear relationship to the other three compounds. This result suggested that **21** probably targeted a specific molecule which was expressed in Panc-1 cells, but not in BxPC-3 and LO2 cells, while such interaction might require high concentrations. These three oligosaccharides were also tested in AsPC-1 cell line, and tetrasacchride **21** exhibit similar effect on AsPC-1 cell line to that of gemcitabine (see Figure S1 in the Supplementary Materials). In addition, these three saccharides showed scarce cytotoxicity effect on the LO2 cell line (Figure 3c), which show great advantage to that of gemcitabine. Although there’re already some compounds for gemcitabineresistant pancreatic cancer, such as nocodazole and combretastatin [5,7], comparatively, sugar compounds often show low or even no cytotoxicity. So, **21** can be a potential novel leading compound for gemcitabine-resistant pancreatic cancer.

## 4. Conclusions

We had synthesized three branches derived from RN1 polysaccharide via [3+2] and [2+2] strategies. Disaccharide **14** was shared as a common acceptor, and different donors derived from **8** were employed. Many intermediates were shared to make the synthetic route more economical. The deprotection was also perform in an efficient way, requiring only two steps, for TBS can be removed with benzylidene together. The thiolphenol byproducts were solved by employing a more active donor (Scheme 1) or a more stable acceptor (Scheme 2). 

The target three oligosaccharides were tested in two different pancreatic cancer cell lines. Pentasaccharides **18** and **27** exhibited similar inhibition at the same mass concentration (about 500 μg/mL) to the published RN1 in BxPC-3. Additionally, tetrasaccharide **21** exhibited high inhibition at 500 μM in Panc-1. All these three RN1derived branches show almost no cytotoxicity in LO2. In conclusion, we’ve found the possible active targeting domain of RN1 (**18** and **27**) and a potential leading compound (**21**) for pancreatic cancer with gemcitabine resistance. The biological mechanism will be investigated in the future.

## Supplementary Materials

The following are available online at www.mdpi.com/2073-4360/9/10/536/s1.
