# Supplementary material for: A Concise Synthesis of Three Branches Derived from Polysaccharide RN1 and Anti-Pancreatic Cancer Activity Study
Source: Polymers (Basel). 2017 Oct 21;9(10):536. doi: 10.3390/polym9100536 (PMC6418633; doi:10.3390/polym9100536)
Supplement: Supplementary file 1 [file polymers-09-00536-s001.pdf]

polymers

Supplementary

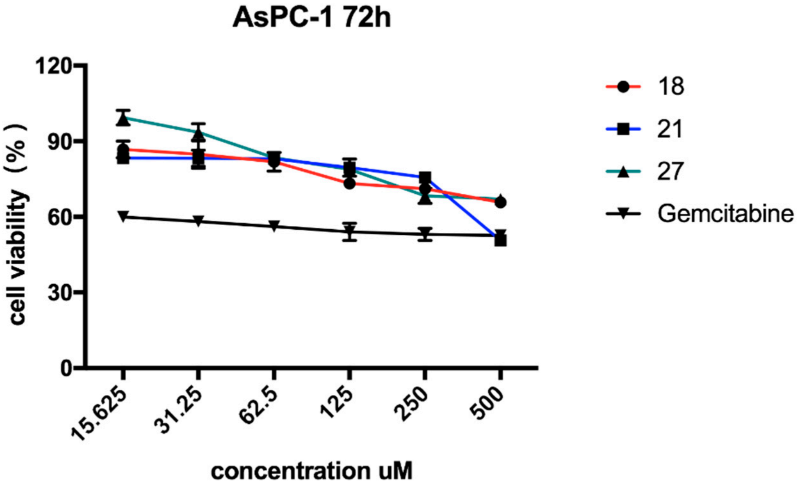

Figure S1. Inhibition effect of oligosaccharides 18, 21 and 27 on pancreatic cell line AsPC-1

Figure legend: Anti-pancreatic cancer activity and cytotoxicity test of oligosaccharides **18**, **21** and **27** on AsPC-1 cells. AsPC-1 cells were treated with compounds 18, 21, 27 and gemcitabine (as a positive control) at concentration of 15.625, 31.24, 62.5, 125, 250 and 500 μM for 72 h followed by MTT test.

General Experimental Procedures

Chemical reagents and solvents were purchased from Sinopharm Chemical Reagent Co. (Shanghai, China) and used without further purification. Reactions were monitored by TLC on glass Silica Gel HSGF254 plates with UV 254 nm detection. TLC staining for carbohydrate samples was performed by dipping the plates into 10% H_2SO_4 in ethanol and drying with a heat gun. Anhydrous dichloromethane (DCM) was freshly distilled from calcium hydride under nitrogen prior to use. Molecular sieves 4 Å powder was purchase from Sigma-Aldrich Co. LLC (USA) and was activated by heating at 200 °C in vacuum for 2 h. Nuclear magnetic resonance (NMR) spectra were measured on a Varian-MERCURY Plus (400 MHz) or/and Bruker AVANCE III (500 MHz). The chemical shifts were assigned in ppm and the coupling constants in Hz. ESI-HRMS spectra were measured on an Agilent 6230 LC-TOF MS spectrometer.

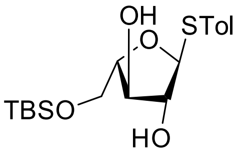

3

Figure S2. *p*-Tolyl 5-O-*tert*-butyldimethylsilyl-1-thio-α-L-arabinofuranoside (3).

To solution of compound **2** (290 mg, 1.13 mmol) and imidazole (154 mg, 2.26 mmol) in DCM (11 mL), t-butyldimethylsilyl chloride (TBSCl, 203 mg, 1.35 mmol) was added, and the reaction mixture was stirred at rt until TLC indicated the end of reaction. Quenched by methanol and concentrated, the residue was purified by silica gel column chromatography using a gradient of EA : PE = 1 : 1.5 afford **3** as transparent syrup (243 mg, 66%).

^{1}H NMR (400 MHz, CDCl3): δ 7.39 (bd, 2H, Aromatic), 7.12 (bd, 2H, Aromatic), 5.49 (s, 1H, H1), 4.30 (m, 1H), 4.25 (d, 1H, J = 10.78 Hz), 4.18-4.09 (m, 2H), 3.84 (m, 2H), 2.82 (d, 1H, J = 9.12 Hz), 2.33 (s, 3H), 0.89 (s, 9H), 0.10 (s, 3H), 0.09 (s, 3H).
^{13}C NMR (125 MHz, CDCl3): δ 138.1, 132.9, 130.1, 129.5, 93.6, 87.1, 81.0, 79.1, 63.6, 25.9, 21.2, 18.5, -5.4, -5.5.
m/z (HRMS) calcd for $C_{18}H_{30}O_4SSiNa^+$: 393.1532, found: 393.1525.

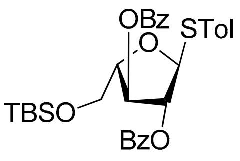

4

Figure S3. ***p***-Tolyl 2,3-di-O-Benzoyl-5-O-*tert*-butyldimethylsilyl-1-thio-α-L-arabinofuranoside (4).

To solution of compound **3** (200 mg, 0.54 mmol) in Pyridine (7 mL), benzoyl chloride (BzCl, 122 μL, 1.05 mmol) was added in ice-bath, and the reaction mixture was stirred in ice-bath until TLC indicated the end of reaction. The resulted mixture was quenched by water and diluted with DCM, washed with 1 M $HCl_{(aq)}$ X 3, $NaHCO3_{(aq)}$ a nd brine. The mixture was concentrated and the residue was purified by silica gel column chromatography using a gradient of EA : PE = 1 : 12 afford **4** as transparent syrup (261 mg, 84%).
^{1}H NMR (400 MHz, CDCl3): δ 8.14-8.01 (m, 4H, Aromatic), 7.63-7.39 (m, 8H, Aromatic), 7.12 (bd, 2H, Aromatic), 5.70 (d, 1H, J = 2.27 Hz), 5.65 (t, 1H, J = 2.14 Hz), 5.60 (dd, 1H, J = 2.01 Hz, J = 4.78 Hz), 4.55 (m, 1H), 4.02 (m, 2H), 2.33 (s, 3H), 0.89 (s, 9H), 0.08 (bd, 6H).
^{13}C NMR (125 MHz, CDCl3): δ 165.7, 165.5, 138.0, 133.6, 132.9, 130.1, 129.9, 129.4, 129.3, 128.6, 128.6, 91.4, 83.6, 82.4, 77.8, 63.1, 26.0, 21.3, 18.5, -5.2.
m/z (HRMS) calcd for $C_{32}H_{38}O_6SSiNa^+$: 601.2056, found: 601.2048.

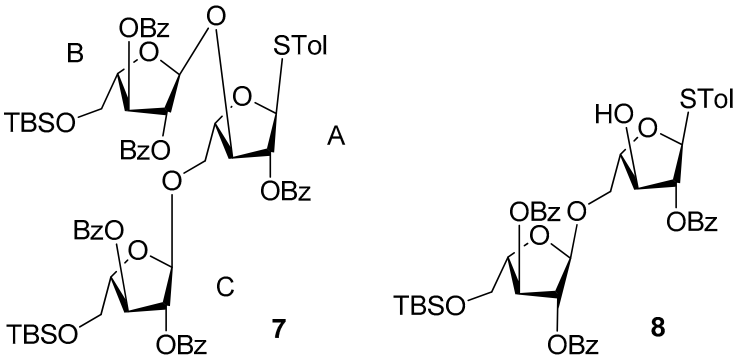

Figure S4. ***p***-Tolyl 2-O-Benzoyl-3,5-di-O-(2,3-di-O-Benzoyl-5-O-*tert*-butyldimethylsilyl-α-L-arabinofuranosyl) -1-thio-α-L-arabinofuranoside (7). ***p***-Tolyl 2-O-Benzoyl-5-O-(2,3-di-O-Benzoyl-5-O-*tert*-butyldimethylsilyl-α-L-arabinofuranosyl)-1-thio-α-L-arabinofuranoside (8).

Preparation of **7** and **8**: Donor **5** (150 mg, 0.24 mmol) and acceptor **6** (62 mg, 0.17 mmol) were dissolved in DCM (8 mL) in the presence of 4 Å molecular sieves (500 mg), the reaction mixture was stirred at RT for 15 min under N_2. TMSOTf (1.5 μL, 8.5 μmol) was injected in ice-bath, and the mixture was stirred for additional 1 h, TLC indicated the completion of the reaction. The reaction was quenched by triethyl amine. The suspension was diluted with DCM , filtered and washed with DCM/MeOH (20/1), concentrated and purified by silica gel column chromatography using a gradient of EA : PE = 1 : 6 to 1 : 4 to afford **7** as white foam solid (39 mg, 18%) and **8** (76 mg, 54%).

Preparation of **8**: Donor **5** (74 mg, 0.12 mmol) and acceptor **6** (39 mg, 0.11 mmol) were dissolved in DCM (2 mL) in the presence of 4 Å molecular sieves (300 mg), the reaction mixture was stirred at RT for 15 min under N_2. TMSOTf (1 μL, 5.5 μmol) was injected in ice-bath, and the mixture was stirred for additional 1 h, TLC indicated the completion of the reaction. The reaction was quenched by

triethyl amine. The suspension was diluted with DCM , filtered and washed with DCM/MeOH (20/1), concentrated and purified by silica gel column chromatography using a gradient of EA : PE = 1 : 4 to afford **8** (65 mg, 74%).

7: ^{1}H NMR (400 MHz, CDCl3): δ 8.18-7.88 (m, 10H, Aromatic), 7.62-7.26 (m, 17H, Aromatic), 7.06 (bd, 2H, Aromatic), 5.67 (s, 1H, $H1^A$), 5.57 (t, 1H, J = 1.64 Hz, $H2^A$), 5.53 (m, 2H), 5.49 (d, 1H, J = 1.12 Hz), 5.47 (m, 2H, $H1^B$), 5.33 (s, 1H, $H1^C$), 4.64 (m, 1H, $H4^A$), 4.57 (m, 1H, $H3^A$), 4.34 (m, 2H), 4.13 (dd, 1H, J = 4.44 Hz, J = 11.48 Hz, $H5a^A$), 3.97-3.87 (m, 5H, $H5b^A$), 2.27 (s, 3H), 0.85 (s, 9H), 0.83 (s, 9H), 0.05 (bd, 6H), 0.02 (s, 6H).

^{13}C NMR (125 MHz, CDCl3): δ 165.7, 165.6, 165.3, 130.1, 129.9, 128.6, 128.5, 106.1, 105.8, 91.4, 84.5, 83.9, 82.2, 81.0, 65.6, 63.0, 62.9, 29.8, 26.0, 21.2, 18.5, -5.2, -5.3.

8: ^{1}H NMR (400 MHz, CDCl3): δ 8.07-7.94 (m, 6H, Aromatic), 7.58 (m, 2H, Aromatic), 7.42 (m, 7H, Aromatic), 7.31 (bt, 2H, Aromatic), 7.08 (bd, 2H, Aromatic), 5.67 (d, 1H, J = 3.61 Hz), 5.49 (m, 2H), 5.31 (s, 1H), 5.16 (t, 1H, J = 3.83 Hz), 4.43 (m, 1H), 4.35 (m, 2H), 4.04 (dd, 1H, J = 4.14 Hz, J = 11.58 Hz), 3.96 (m, 2H), 3.88 (dd, 1H, J = 3.58 Hz, J = 11.59 Hz), 3.50 (d, 1H, J = 2.97 Hz), 2.29 (s, 3H), 0.89 (s, 9H), 0.09(s, 6H).

^{13}C NMR (125 MHz, CDCl3): δ 167.5, 165.8, 165.5, 138.1, 133.9, 133.6, 133.4, 132.7, 130.1, 129.9, 129.5, 129.4, 129.0, 128.7, 128.6, 128.5, 106.3, 89.4, 86.9, 83.6, 82.4, 81.7, 76.7, 65.9, 63.0, 26.0, 21.3, 18.5, -5.1, -5.2.

7: m/z (HRMS) calcd for $C_{69}H_{80}O_{17}SSi_2Na^+$: 1291.4552, found: 1291.4541.

8: m/z (HRMS) calcd for $C_{44}H_{50}O_{11}SSiNa^+$: 837.2741, found: 837.2734.

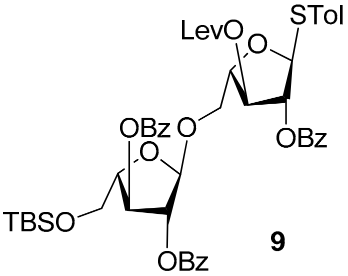

9

Figure S5. ***p***-Tolyl 2-O-Benzoyl-3-O-levulinoyl-5-O-(2,3-di-O-Benzoyl-5-O-*tert*-butyldimethylsilyl-*α*-L-arabinofuranosyl)-1-thio-*α*-L-arabinofuranoside (9).

To stirred solution of compound **8** (62 mg, 76 μmol), 3-(3-dimethylaminopropyl)-1-ethylcarbodiimide hydrochloride (EDCI, 29 mg, 0.15 mmol) and 4-dimethylaminopyridine (DMAP, 4.6 mg, 38 μmol) in DCM (2 mL), Levulinic acid (LevOH, 11 μL, 0.11 mmol) were added at RT, TLC indicated the completion of reaction and quenched with methanol, stirred for additional 15 min. The mixture was concentrated and the residue was purified by silica gel column chromatography using a gradient of EA : PE = 1 : 4 to afford **9** as transparent syrup (60 mg, 87%).

^{1}H NMR (400 MHz, CDCl3): δ 8.07-7.89 (m, 6H, Aromatic), 7.61-7.26 (m, 19H, Aromatic), 7.06 (bd, 2H, Aromatic), 5.63 (d, 1H, J = 1.78 Hz), 5.54-5.45 (m, 4H), 5.33 (s, 1H), 4.54 (m, 1H), 4.39 (m, 1H), 4.12 (dd, 1H, J = 4.17 Hz, J = 11.30 Hz), 3.96 (m, 2H), 3.86 (dd, 1H, J = 2.92 Hz, J = 11.29 Hz), 2.76 (m, 2H), 2.63 (m, 2H), 2.29 (s, 3H), 2.18 (s, 3H), 0.87 (s, 9H), 0.06 (s, 6H).

^{13}C NMR (125 MHz, CDCl3): δ 206.1, 171.7, 165.6, 165.3, 137.9, 133.4, 133.1, 132.6, 129.9, 129.8, 129.4, 128.9, 128.5, 128.4, 128.3, 105.9, 91.2, 83.4, 82.2, 82.0, 81.4, 65.5, 62.8, 37.8, 29.6, 27.8, 25.9, 21.0, 18.4, -5.3.

m/z (HRMS) calcd for $C_{49}H_{56}O_{13}SSiNa^+$: 935.3109, found: 935.3103.

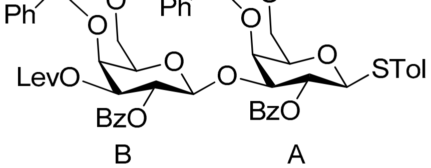

B A

12

Figure S6. *p*-tolyl 2-O-benzoyl-3-O-(2-O-benzoyl-3-O-levulinoyl-4, 6-O-benzylidene-β-D-galactopyranosyl)-4, 6-O-benzylidene-1-thio-β-D-galactopyranoside (12).

Donor **10** (443 mg, 0.72 mmol) and acceptor **11** (314 mg, 0.66 mmol) were dissolved in DCM (6.4 mL) in the presence of 4 Å molecular sieves (1.5 g), the reaction mixture was stirred at RT for 15 min under N_2. TMSOTf (1.5 μL, 8.5 μmol) was injected at -78 ℃, and the mixture was stirred for additional 1 h, TLC indicated the completion of the reaction. The reaction was quenched by triethyl amine. The suspension was diluted with DCM , filtered and washed with DCM/MeOH (20/1), concentrated and purified by silica gel column chromatography using a gradient of EA : PE : DCM = 1 : 1 : 1 to 1 : 1 : 2 to afford **12** as white foam solid (379 mg, 62%).

^{1}H NMR (400 MHz, CDCl3): δ 7.91 (bd, 2H, Aromatic), 7.72 (bd, 2H, Aromatic), 7.58-7.20 (m, 18H, Aromatic), 6.98 (bd, 2H, Aromatic), 5.57 (dd, 1H, J = 8.03 Hz, J = 10.25 Hz, $H2^A$), 5.47 (s, 1H), 5.43 (t, 1H, J = 9.59 Hz, $H2^B$), 5.39 (s, 1H), 4.94 (m, 2H, $H1^A$ and $H3^A$), 4.77 (d, 1H, J = 9.70 Hz, $H1^B$), 4.42 (d, 1H, J = 3.2 Hz), 4.32 (m, 3H, $H3^B$ and $H4^A$), 4.09 (dd, 1H, J = 1.03 Hz, J = 12.26 Hz), 3.98 (m, 2H), 3.52 (s, 1H), 3.44 (s, 1H), 2.58-2.33 (m, 4H), 2.28 (s, 3H), 1.86 (s, 3H).

^{13}C NMR (125 MHz, CDCl3): δ 206.3, 172.1, 165.3, 164.8, 134.0, 133.1, 129.9, 129.8, 129.6, 129.3, 128.8, 128.4, 128.0, 126.6, 101.1, 101.0, 100.2, 86.2, 76.5, 76.1, 73.4, 72.3, 70.3, 69.2, 69.0, 68.8, 66.6, 37.8, 29.8, 29.5, 28.3, 21.3.

m/z (HRMS) calcd for $C_{52}H_{50}O_{14}SNa^+$: 953.2819, found: 953.2811.

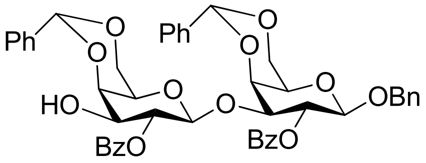

14

Figure S7. Benzyl 2-O-benzoyl-3-O-(2-O-benzoyl-4, 6-O-benzylidene-β-D-galactopyranosyl)-4, 6-O-benzylidene-β-D-galactopyranoside (14).

To solution of donor **12** (222 mg, 0.24 mmol) and benzyl alcohol (50 μL, 0.48 mmol) in DCM (4.8 mL) in the presence of 4 Å molecular sieves (500 mg), the reaction mixture was stirred at RT for 15 min under N_2. NIS (70 mg, 0.31 mmol) was added carefully. TMSOTf (1.5 μL, 8.5 μmol) was injected at -78 ℃, and the mixture was stirred for additional 1 h, TLC indicated the completion of the reaction. The reaction was quenched by triethyl amine. The suspension was diluted with DCM , filtered and washed with DCM/MeOH (20/1), and the organic layer was washed with sodium hyposulfite and sodium bicarbonate solution and brine, followed by the concentration. The obtained residue was taken up with DCM/MeOH (1/1, 5 mL). Acetic acid (137 μL, 2.4 mmol) and hydrazine hydrate (60 μL, 1.2 mmol) were added at RT, TLC indicated the completion of reaction. The reaction solution was diuted with DCM and washed with saturated sodium bicarbonate aqueous solution, water and brine, dried over sodium sulfate. The mixture was concentrated and the residue was purifiedby silica gel column chromatography using a gradient of EA : DCM = 1 : 10 to afford **14** as transparent syrup (140 mg, 72% in two steps).

^{1}H NMR (400 MHz, CDCl3): δ 7.91 (bd, 2H, Aromatic), 7.72 (bd, 2H, Aromatic), 7.58 (bt, 1H, Aromatic), 7.52-7.05 (m, 20H, Aromatic), 5.68 (dd, 1H, J = 7.91 Hz, J = 10.23 Hz), 5.52 (s, 1H), 5.44 (s, 1H), 5.32 (dd, 1H, J = 7.91 Hz, J = 10.23 Hz), 4.95 (d, 1H, J = 8.02 Hz), 4.85 (d, 1H, J = 12.72 Hz), 4.64 (d, 1H, J = 12.68 Hz), 4.58 (d, 1H, J = 7.92 Hz), 4.35 (m, 2H), 4.19 (m, 3H), 4.02 (m, 2H), 3.68 (td, 1H, J = 3.77 Hz, J = 10.60 Hz), 3.44 (bd, 2H), 2.65 (d, 1H, J = 10.64 Hz).

^{13}C NMR (125 MHz, CDCl3): δ 166.7, 165.0, 137.7, 137.5, 137.4, 133.1, 130.4, 129.9, 129.7, 129.5, 128.8, 128.7, 128.5, 128.4, 128.3, 128.1, 127.9, 127.6, 127.1, 126.5, 126.4, 101.5, 101.0, 100.0, 99.8, 76.3, 75.7, 75.3, 72.8, 72.2, 70.8, 69.7, 69.0, 67.0, 66.9.

m/z (HRMS) calcd for $C_{47}H_{44}O_{13}Na^+$: 839.2680, found: 839.2670.

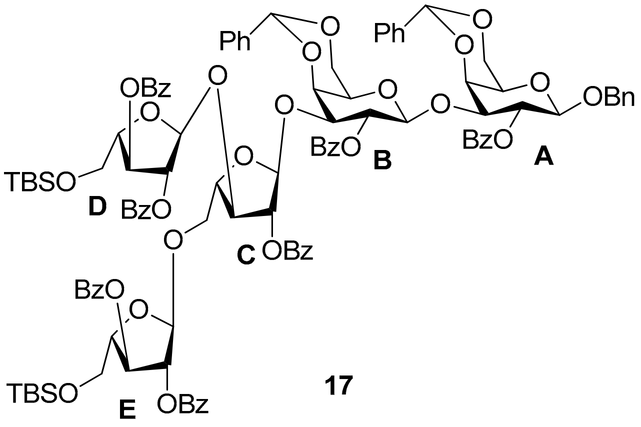

17

Figure S8. Benzyl 2-O-benzoyl-3-O-{2-O-benzoyl-3-O-[2-O-Benzoyl-3,5-di-O-(2,3-di-O-Benzoyl-5-O-*tert*-butyldimethylsilyl-α-L-arabinofuranosyl)-α-L-arabinofuranosyl]-4, 6-O-benzylidene-β-D-galacto- pyranosyl}-4, 6-O-benzylidene-β-D-galactopyranoside (17).

To solution of donor **7** (118 mg, 93 μmol) and acceptor (69 mg, 84 μmol) in DCM (4 mL) in the presence of 4 Å molecular sieves (500 mg), the reaction mixture was stirred at RT for 15 min under N_2. NIS (28 mg, 0.12 mmol) was added carefully. TMSOTf (1.5 μL, 8.5 μmol) was injected at 0 ℃, and the mixture was stirred for additional 2 h, TLC indicated the completion of the reaction. The reaction was quenched by triethyl amine. The suspension was diluted with DCM, filtered and washed with DCM/MeOH (20/1), and the organic layer was washed with sodium hyposulfite and sodium bicarbonate solution and brine, followed by the concentration. The obtained residue was purified by silica gel column chromatography using a gradient of EA : PE : DCM = 1 : 2 : 1.5 to afford **17** as white foam solid (118 mg, 71%).

^{1}H NMR (500 MHz, CDCl3): δ 8.05-7.05 (m, 50H, Aromatic), 5.63-5.50 (m, 3H, H2^{A}, H2^{B} and PhC*H*), 5.46 (m, 2H), 5.43 (d, 1H, J = 1.2 Hz), 5.41 (bd, 1H), 5.38 (bs, 2H, H1^{D} and PhC*H*), 5.27 (d, 1H, J = 2.57 Hz, H2^{C}), 5.15 (bd, 2H, H1^{E} and H1^{C}), 4.89 (d, 1H, J = 8.16 Hz, H1^{B}), 4.84 (d, 1H, J = 12.66 Hz, PhC*H*H), 4.62 (d, 1H, J = 12.66 Hz, PhCH*H*), 4.58 (d, 1H, J = 7.93 Hz, H1^{A}), 4.41 (m, 2H, H4^{C} and H4^{A}), 4.31 (m, 4H, H3^{C}), 4.25 (dd, 1H, J = 3.47 Hz, J = 10.13, H3^{A}), 4.08 (m, 1H), 4.04-3.96 (m, 2H), 3.96-3.83 (m, 4H, H5a^{C}), 3.81-3.71 (m, 4H, H5b^{C} and H3^{B}), 3.36 (s, 1H), 3.14 (s, 1H), 0.85 (s, 9H), 0.78 (s, 9H), 0.04 (s, 6H), -0.04 (s, 6H).

^{13}C NMR (125 MHz, CDCl3): δ 165.7, 165.6, 165.4, 165.1, 165.0, 164.9, 137.9, 137.8, 137.5, 133.6, 133.4, 133.3, 133.1, 132.9, 132.5, 130.5, 130.1, 130.0, 129.9, 129.8, 129.4, 129.2, 129.0, 128.6, 128.5, 128.4, 128.3, 128.1, 128.0, 127.9, 127.6, 126.4, 126.2, 107.9, 106.0, 105.3, 100.9, 100.8, 99.8, 99.7, 84.4, 83.5, 82.9, 82.2, 80.8, 80.1, 78.2, 77.5, 75.8, 74.1, 70.6, 70.5, 69.7, 69.0, 68.8, 67.0, 66.0, 63.0, 62.8, 26.0, 18.5, 18.4, -5.2, -5.3.

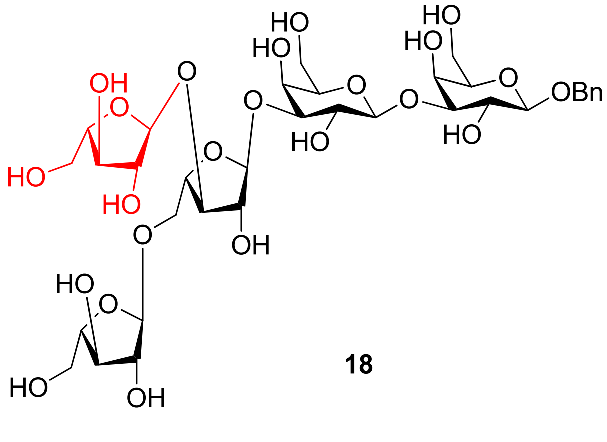

18

Figure S9. Benzyl 3-O-{3-O-[3,5-di-O-(α-L-arabinofuranosyl)-α-L-arabinofuranosyl]-β-D-galactopyranosyl}-β-D-galactopyranoside (18).

Pentasaccharide **17** (20 mg, 10 μmol) was dissolved in 80% acetic acid aqueous solution (2 mL) at 80 ℃ and continue heating at 80 ℃ until TLC indicated the end of reaction. After concentration and co-evaporated with toluene, the residue was taken up with methanol (2 mL) and the solution was regulated by NaOMe to pH about 10. The mixture was allowed to stir over night and quenched by acetic acid to pH about 5. The mixture was concentrated and the residue was taken up with water and further purified by "P2" column to afford **18** as white form solid (6.6 mg, 79% in two steps).

^{1}H NMR (500 MHz, D_2O): δ 7.61 (m, 5H, Aromatic), 5.43 (s, 1H), 5.34 (s, 1H), 5.24 (s, 1H), 5.12 (d, 1H, J = 11.60 Hz), 4.93 (d, 1H, J = 11.66 Hz), 4.82 (d, 1H, J = 7.05 Hz), 4.69 (d, 1H, J = 7.92 Hz), 4.51 (m, 2H), 4.36 (d, 1H, J = 2.86 Hz), 4.31-4.18 (m, 6H), 4.11 (m, 3H), 4.03-3.83 (m, 15H).

^{13}C NMR (125 MHz, D_2O): δ 136.6, 128.8, 128.7, 128.5, 109.4, 107.3, 107.2, 104.1, 101.4, 84.2, 84.0, 82.4, 81.5, 81.1, 80.9, 79.8, 79.6, 76.5, 74.9, 74.8, 71.3, 70.2, 69.9, 68.5, 68.4, 66.4, 61.2, 61.1, 60.9.

m/z (HRMS) calcd for $C_{34}H_{52}O_{23}Na^+$: 851.2797, found: 851.2790.

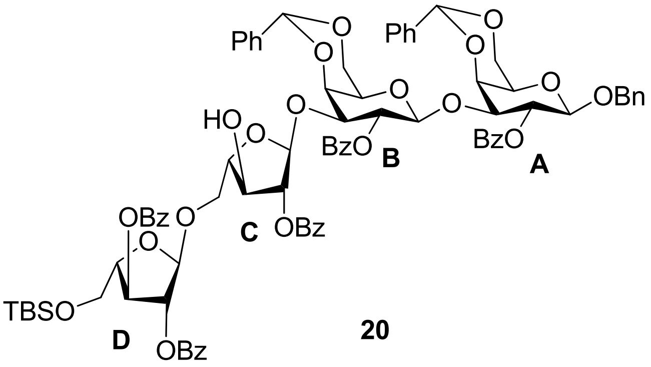

20

Figure S10. Benzyl 2-O-benzoyl-3-O-{2-O-benzoyl-3-O-[2-O-Benzoyl-5-O-(2,3-di-O-Benzoyl-5-O-*tert*butyl-dimethylsilyl-α-L-arabinofuranosyl)-α-L-arabinofuranosyl]-4, 6-O-benzylidene-β-D-galacto-pyranosyl}-4, 6-O-benzylidene-β-D-galactopyranoside (20).

To solution of donor **9** (37 mg, 40 μmol) and acceptor **14** (30 mg, 37 μmol) in DCM (2.0 mL) in the presence of 4 Å molecular sieves (300 mg), the reaction mixture was stirred at RT for 15 min under N_2. NIS (16 mg, 71 μmol) was added carefully. TMSOTf (0.7 μL, 3.8 μmol) was injected at 0 ℃, and the mixture was stirred for additional 1 h, TLC indicated the completion of the reaction. The reaction was quenched by triethyl amine. The suspension was diluted with DCM, filtered and washed with DCM/MeOH (20/1), and the organic layer was washed with sodium hyposulfite and sodium bicarbonate solution and brine, followed by the concentration. The obtained residue was taken up with DCM/MeOH (1/1, 2 mL). Acetic acid (44 μL, 0.77 mmol) and hydrazine hydrate (19 μL, 0.38 mmol) were added at RT, TLC indicated the completion of reaction. The reaction solution was diuted with DCM and washed with saturated sodium bicarbonate aqueous solution, water and brine, dried over sodium sulfate. The mixture was concentrated and the residue was purified by silica gel column chromatography using a gradient of EA : PE : DCM = 1 : 2 : 3 to afford **20** as white foam solid (26 mg, 47% in two steps).

^{1}H NMR (500 MHz, CDCl3): δ 8.06-7.90 (m, 6H, Aromatic), 7.78 (bd, 2H, Aromatic), 7.66-7.05 (m, 32H), 5.63 (dd, 1H, J = 7.95 Hz, J = 10.11 Hz, $H2^A$), 5.58 (s, 1H), 5.51 (m, 2H, $H2^B$), 5.46 (d, 1H, J = 1.38 Hz), 5.42 (s, 1H), 5.26 (s, 1H, $H1^D$), 5.22 (s, 1H, $H1^C$), 4.94 (m, 2H, , $H1^B$), 4.85 (d, 1H,), 4.64 (d,1H, J = 12.67 Hz), 4.59 (d, 1H, J = 7.92 Hz, $H1^A$), 4.42-4.29 (m, 5H, $H4^B$, $H4^A$ and $H6a^A$), 4.25 (dd, 1H, J = 6.50 Hz, J = 10.16 Hz, $H3^A$), 4.12-3.85 (m, 7H, $H5b^C$, $H6a^B$, $H6b^B$ and $H6b^A$), 3.83 (dd, 1H, J = 3.56 Hz, J = 10.03 Hz, $H3^B$), 3.76 (dd, 1H, J = 3.71 Hz, J = 11.51 Hz, $H5b^C$), 3.52 (d, 1H, J = 6.53 Hz), 3.42 (s, 1H, $H5^A$), 3.33 (s, 1H, $H5^B$), 0.88 (s, 9H), 0.07 (s, 6H).

^{13}C NMR (125 MHz, CDCl3): δ 166.1, 165.8, 165.6, 165.4, 164.9, 137.8, 137.7, 137.4, 133.7, 133.5, 133.4, 130.0, 129.9, 129.8, 129.4, 129.3, 129.2, 128.7, 128.6, 128.5, 128.4, 128.3, 128.0, 127.9, 127.6, 126.4,

106.8, 105.9, 101.1, 100.9, 99.9, 99.8, 84.8, 83.6, 83.4, 82.3, 76.4, 76.0, 75.7, 74.7, 70.7, 70.6, 69.7, 69.0, 68.8, 67.0, 66.8, 66.6, 62.9, 29.8, 26.0, 18.5, -5.2.

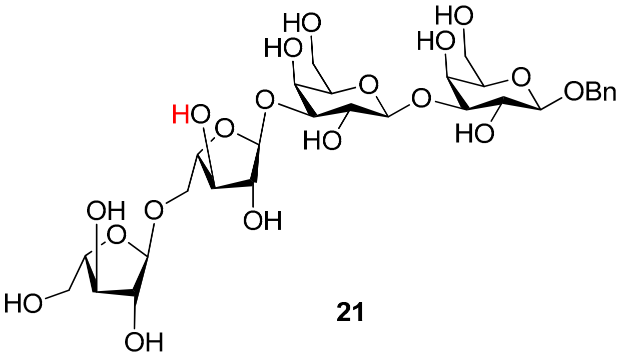

21

Figure S11. Benzyl 3-O-{3-O-[5-O-(α-L-arabinofuranosyl)-α-L-arabinofuranosyl]-β-D-galactopyranosyl}-β-D-galactopyranoside (18).

The deprotection of **20** is similar to that of **18**, and afford **21** as white foam solid (5.8 mg, 56% in two steps).

^{1}H NMR (500 MHz, D2O): δ 7.46-7.34 (m, 5H, Aromatic), 5.19 (s, 1H), 5.02 (s, 1H), 4.91 (d, 1H, J = 11.63 Hz), 4.71 (d, 1H), 4.60 (m, 1H), 4.47 (d, 1H, J = 7.87 Hz), 4.17 (m, 3H), 4.04 (m, 3H), 3.96 (dd, 1H, J = 3.33 Hz, J = 6.04 Hz), 3.90 (dd, 1H, J = 3.32 Hz, J = 5.90 Hz), 3.86-3.62 (m, 14H).

^{13}C NMR (125 MHz, D_2O): δ 136.6, 128.8, 128.7, 128.5, 109.3, 107.4, 104.1, 101.4, 83.9, 82.4, 82.2, 81.2, 80.9, 80.1, 76.7, 76.5, 74.9, 74.8, 71.3, 70.2, 69.9, 68.5, 66.8, 61.2, 60.9.

m/z (HRMS) calcd for $C_{29}H_{44}O_{19}Na^{+}$: 719.2374, found: 719.2366.

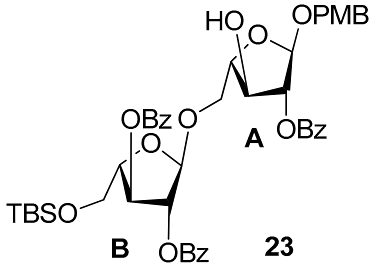

23

Figure S12. *p*-methoxylbenzyl 2-O-Benzoyl-5-O-(2,3-di-O-Benzoyl-5-O-*tert*-butyldimethylsilyl-α-L-arabino-furanosyl)-α-L-arabinofuranoside (23).

To solution of donor **9** (57 mg, 62 μmol) and *p*-methoxylbenzyl alcohol (11.7 μL, 93 μmol) in DCM (1.5 mL) in the presence of 4 Å molecular sieves (300 mg), the reaction mixture was stirred at RT for 15 min under N_2. NIS (18 mg, 80 μmol) was added carefully. TMSOTf (1.1 μL, 6.2 μmol) was injected at 0 ℃, and the mixture was stirred for additional 1 h, TLC indicated the completion of the reaction. The reaction was quenched by triethyl amine. The suspension was diluted with DCM, filtered and washed with DCM/MeOH (20/1), and the organic layer was washed with sodium hyposulfite and sodium bicarbonate solution and brine, followed by the concentration. The obtained residue was taken up with DCM/MeOH (1/1, 2 mL). Acetic acid (66 μL, 1.1 mmol) and hydrazine hydrate (29 μL, 0.58 mmol) were added at RT, TLC indicated the completion of reaction. The reaction solution was diuted with DCM and washed with saturated sodium bicarbonate aqueous solution, water and brine, dried over sodium sulfate. The mixture was concentrated and the residue was purifiedby silica gel column chromatography using a gradient of EA : PE = 1 : 4 to afford **23** (39 mg, 75% in two steps).

^{1}H NMR (500 MHz, CDCl3): δ 8.09-7.92 (m, 6H, Aromatic), 7.63-7.24 (m, 13H), 6.87 (bd, 2H, Aromatic), 5.50 (m, 2H), 5.34 (s, 1H), 5.30 (s, 1H), 5.15 (d, 1H, J = 1.8 Hz), 4.75 (d, 1H, J = 11.29 Hz), 4.50 (d, 1H, J = 11.51 Hz), 4.38 (m, 2H), 4.24 (m, 1H), 4.01 (m, 3H), 3.88 (m, 5H), 3.28 (d, 1H, J = 5.52 Hz), 0.88 (s, 9H), 0.08 (s, 6H).

^{13}C NMR (125 MHz, CDCl3): δ 166.9, 165.8, 165.5, 159.6, 130.1, 130.0, 129.9, 128.7, 128.6, 128.5, 114.1, 106.2, 104.2, 85.9, 83.6, 82.9, 82.4, 69.0, 66.6, 63.0, 55.4, 26.0, 18.5, -5.1, -5.2.

m/z (HRMS) calcd for $C_{45}H_{52}O_{13}SiNa^+$: 851.3075, found: 851.3067.

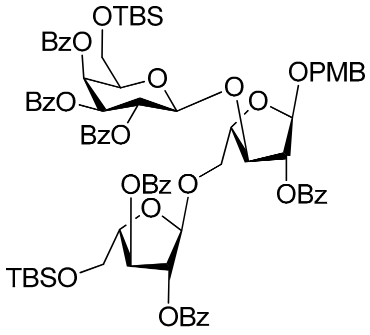

24

Figure S13. *p*-methoxylbenzyl 2-O-Benzoyl-3-O-(2,3,4-tri-benzoyl-6-O-*tert*-butyldimethylsilyl-β-D-galacto-pyranosyl)-5-O-(2,3-di-O-Benzoyl-5-O-*tert*-butyldimethylsilyl-α-L-arabinofuranosyl)-α-L-arabinofuranoside (24).

Donor **16** (48 mg, 62 μmol) and acceptor **23** (44 mg, 52 μmol) were dissolved in DCM (3 mL) in the presence of 4 Å molecular sieves (300 mg), the reaction mixture was stirred at RT for 15 min under N_2. TMSOTf (1.5 μL, 8.2 μmol) was injected in ice-bath, and the mixture was stirred for an additional 1.5 h, TLC indicated the completion of the reaction. The reaction was quenched by triethyl amine. The suspension was diluted with DCM , filtered and washed with DCM/MeOH (20/1), concentrated and purified by silica gel column chromatography using a gradient of EA : PE = 1 : 5 to afford **24** (48 mg, 65%).

^{1}H NMR (500 MHz, CDCl3): δ 8.09-7.92 (m, 10H, Aromatic), 7.79 (bd, 2H, Aromatic), 7.61-7.20 (m, 18H, Aromatic), 6.90 (bd, 2H, Aromatic), 6.72 (bd, 2H, Aromatic), 5.91 (d, 1H, J = 3.26 Hz), 5.75 (dd, 1H, J = 8.14 Hz, J = 10.34 Hz), 5.59 (dd, 1H, J = 3.41Hz, J = 10.38 Hz), 5.50 (s, 1H), 5.47 (d, 1H, J = 4.86 Hz), 5.35 (s, 1H), 5.28 (d, 1H, J = 8.07 Hz), 5.26 (s, 1H), 5.05 (s, 1H), 4.42 (m, 2H), 4.30 (m, 3H), 4.00 (m, 3H), 3.87 (m, 2H), 3.75 (s, 3H), 3.65 (m, 2H), 0.86 (s, 9H), 0.74 (s, 9H), 0.07 (s, 6H), -0.14 (s, 3H), -0.19 (s, 3H).

^{13}C NMR (125 MHz, CDCl3): δ 165.7, 165.6, 165.4, 159.1, 130.1, 130.0, 129.9, 129.5, 128.6, 128.5, 113.7, 106.0, 103.8, 100.3, 84.0, 83.0, 82.3, 82.2, 81.8, 77.5, 74.0, 72.3, 69.8, 67.9, 67.5, 66.0, 63.0, 60.6, 55.3, 26.0, 25.8, 18.5, 18.1, -5.1, -5.2, -5.6, -5.7.

m/z (HRMS) calcd for $C_{78}H_{88}O_{21}Si_2Na^+$: 1439.5254, found: 1439.5232.

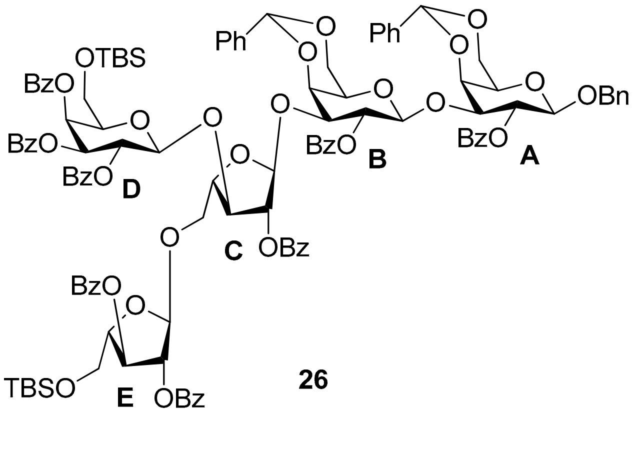

26

Figure S14. Benzyl 2-O-benzoyl-3-O-{2-O-benzoyl-3-O-[2-O-Benzoyl-3-O-(2,3,4-tri-benzoyl-6-O-*tert*-butyl-dimethylsilyl-β-D-galactopyranosyl)-5-O-(2,3-di-O-Benzoyl-5-O-*tert*-butyldimethylsilyl-*α*-L-arabinofuranosyl)-*α*-L-arabinofuranosyl]-4, 6-O-benzylidene-β-D-galactopyranosyl}-4, 6-O-benzylidene-β-D-galactopyranoside (26).

Donor **25** (27 mg, 18 μmol) and acceptor **14** (15 mg, 19 μmol) were dissolved in DCM (2 mL) in the presence of 4 Å molecular sieves (300 mg), the reaction mixture was stirred at RT for 15 min under N_2. TMSOTf (1.0 μL, 5.5 μmol) was injected in ice-bath, and the mixture was stirred for additional 1.5 h, TLC indicated the completion of the reaction. The reaction was quenched by triethyl amine. The suspension was diluted with DCM , filtered and washed with DCM/MeOH (20/1), concentrated and purified by silica gel column chromatography using a gradient of EA : PE : DCM = 1 : 3 : 2 to afford **26** (24 mg, 62%).

^{1}H NMR (500 MHz, CDCl3): δ8.06-7.02 (m, 55H, aromatic), 5.83 (d, 1H, J = 3.30 Hz, $H4^{D}$), 5.64 (dd, 1H, J = 8.04 Hz, J = 10.34 Hz, $H2^{D}$), 5.58 (dd, 1H, J = 7.98 Hz, J = 10.10 Hz, $H2^{A}$), 5.47 (m, 4H, $H3^{D}$), 5.40 (s, 1H), 5.29 (dd, 1H, J = 8.07 Hz, J = 10.10 Hz, $H2^{B}$), 5.20 (s, 1H, $H1^{E}$), 5.18 (d, 1H, J = 2.86 Hz, $H2^{C}$), 5.11 (s, 1H, $H1^{C}$), 5.00 (d, 1H, J = 8.04 Hz, $H1^{D}$), 4.88 (d, 1H, J = 8.05 Hz, $H1^{B}$), 4.83 (d, 1H, J = 12.68 Hz, PhC*H*H), 4.62 (d, 1H, J = 12.59 Hz, PhCH*H*), 4.57 (d, 1H, J = 7.95 Hz, $H1^{A}$), 4.40 (d, 1H, J = 3.45 Hz, $H4^{A}$), 4.31 (m, 4H, $H6a^{A}$ and $H3^{C}$), 4.21 (dd, 1H, J = 3.45 Hz, J = 10.14 Hz, $H3^{A}$), 4.13-3.69 (m, 10H, $H6b^{A}$, $H3^{B}$, $H4^{B}$, $H6a^{B}$, $H6b^{B}$, $H5a^{C}$, $H5b^{C}$ and $H5^{D}$), 3.59 (m, 2H), 3.39 (s, 1H, $H5^{A}$), 3.09 (s, 1H, $H5^{B}$), 0.87 (s, 9H), 0.73 (s, 9H), 0.06 (s, 6H), -0.17 (s, 3H), -0.21 (s, 3H).

^{13}C NMR (125 MHz, CDCl3): δ 165.7, 165.6, 165.5, 165.4, 165.3, 165.0, 164.8, 163.4, 137.8, 133.6, 133.2, 130.0, 129.9, 129.8, 129.7, 129.5, 128.6, 128.4, 128.3, 128.0, 127.9, 127.6, 126.4, 126.2, 107.2, 106.1, 100.9, 100.6, 99.9, 99.8, 83.5, 83.0, 82.5, 82.2, 80.2, 77.6, 77.5, 76.0, 75.9, 74.4, 74.0, 72.2, 70.7, 70.6, 69.7, 69.6, 69.0, 68.8, 67.8, 67.0, 66.9, 62.9, 60.5, 26.0, 25.8, 18.5, 18.1, -5.2, -5.6, -5.7.

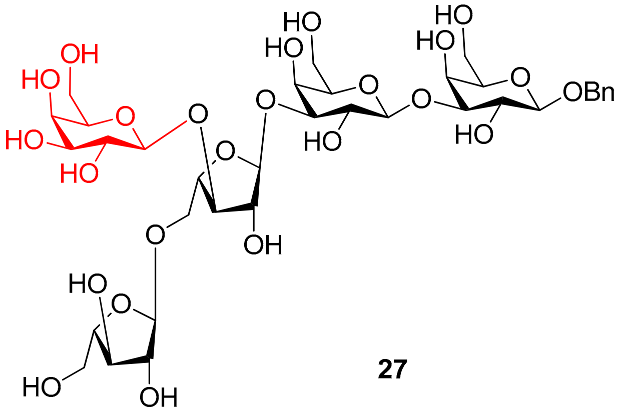

27

Figure S15. Benzyl 3-O-{3-O-[3-O-(β-D-galactopyranosyl)-5-O-(*α*-L-arabinofuranosyl)-*α*-L-arabinofuranosyl]-β-D-galactopyranosyl}-β-D-galactopyranoside (27).

The deprotection of **26** is similar to that of **18**, and afford **27** as white foam solid (7.6 mg, 84% in two steps).

^{1}H NMR (500 MHz, D2O): δ 7.40-7.27 (m, 5H, Aromatic), 5.16 (s, 1H), 4.97 (s, 1H), 4.85 (d, 1H, J = 11.58 Hz), 4.66 (d, 1H, J = 11.64 Hz), 4.54 (d, 1H, J = 7.54 Hz), 4.42 (m, 2H), 4.29 (m, 2H), 4.09 (m, 2H), 3.99 (m, 3H), 3.84 (m, 3H), 3.76 (dd, 1H, J = 2.55 Hz, J = 11.44 Hz), 3.73-3.53 (m, 16H), 3.42 (dd, 1H, J = 7.95 Hz, J = 9.68 Hz).

^{13}C NMR (125 MHz, D_2O): δ 136.6, 128.8, 128.7, 128.5, 109.3, 107.4, 104.1, 102.7, 101.4, 84.5, 84.0, 82.4, 82.1, 80.9, 79.7, 79.6, 76.5, 75.3, 74.9, 74.8, 72.5, 71.3, 70.7, 70.2, 69.9, 68.5, 66.5, 61.2, 60.9,

m/z (HRMS) calcd for $C_{35}H_{54}O_{24}Na^{+}$: 881.2903, found: 881.2898.

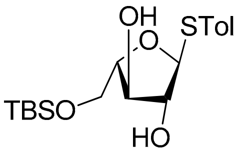

3

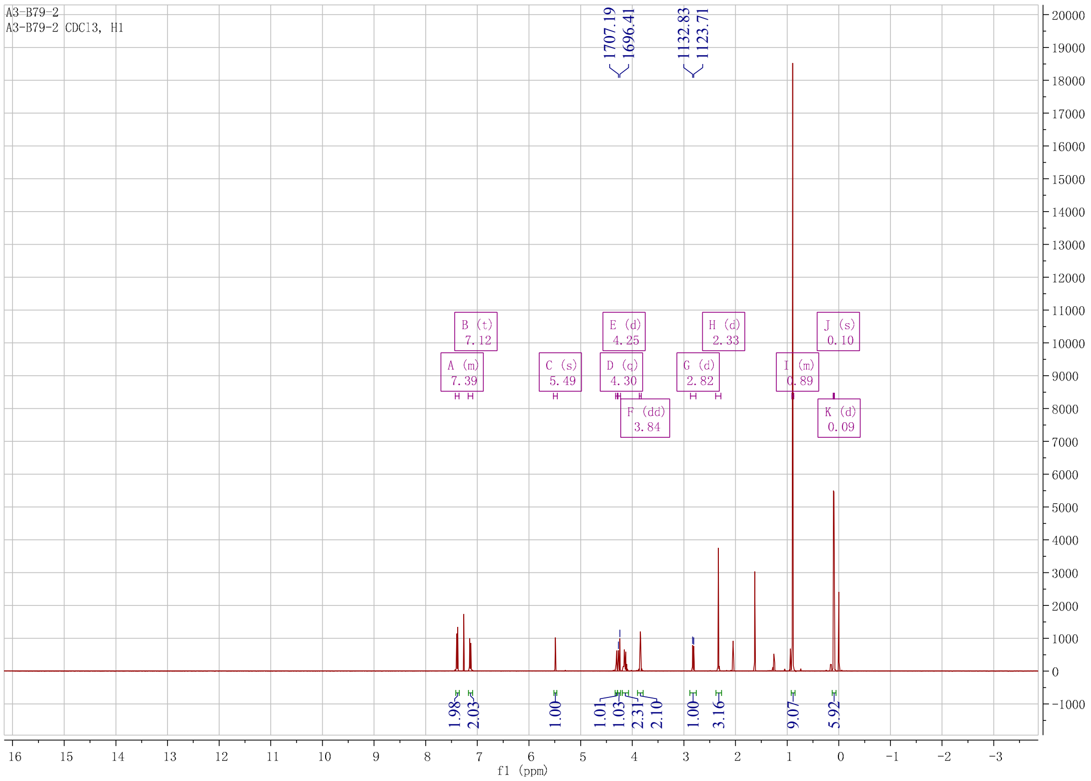

Figure S16. ^{1}H-NMR (CDCl3, 400 MHz) of 3

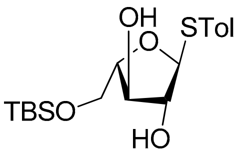

3

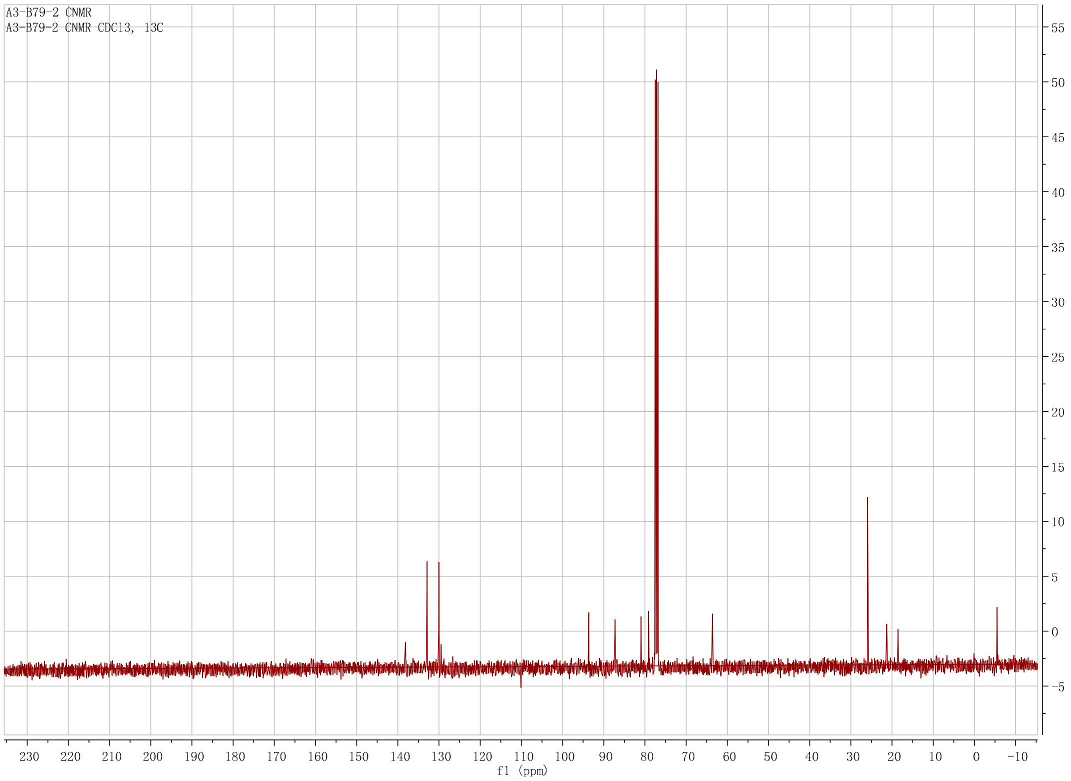

Figure S17. ^{13}C-NMR (CDCl3, 100 MHz) of 3

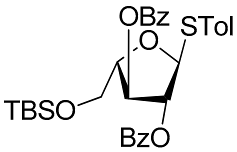

4

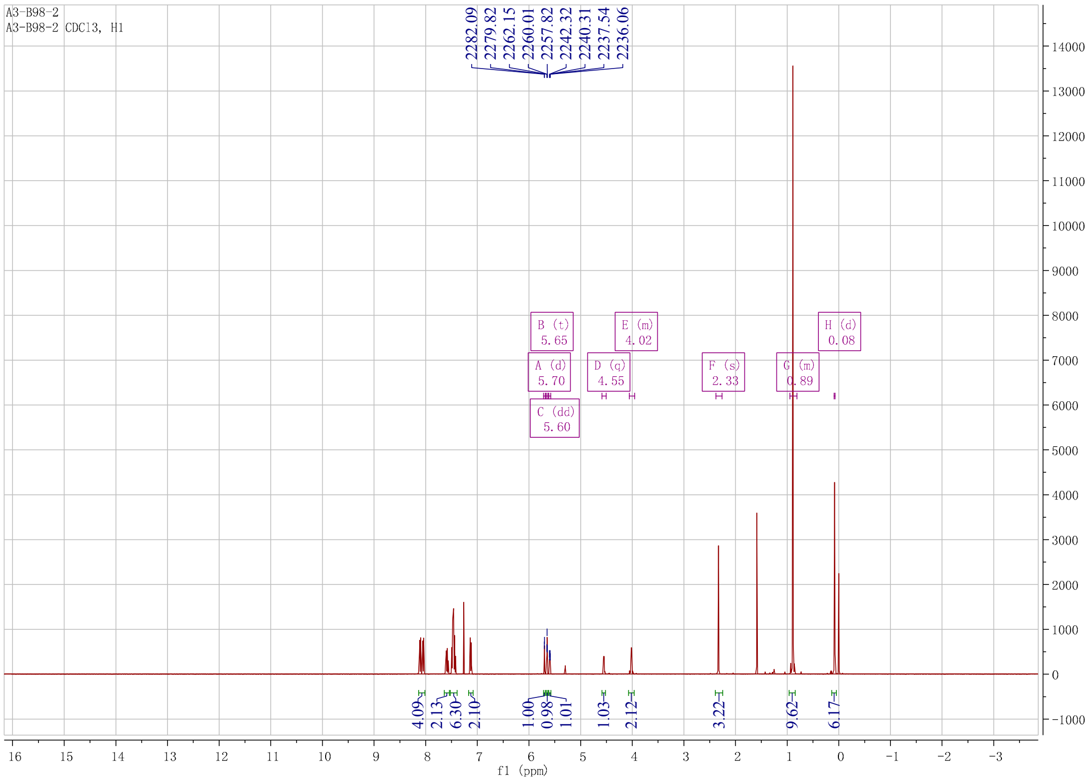

Figure S18. ^{1}H-NMR (CDCl3, 400 MHz) of 4

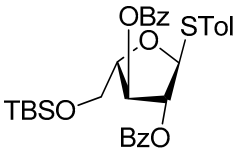

4

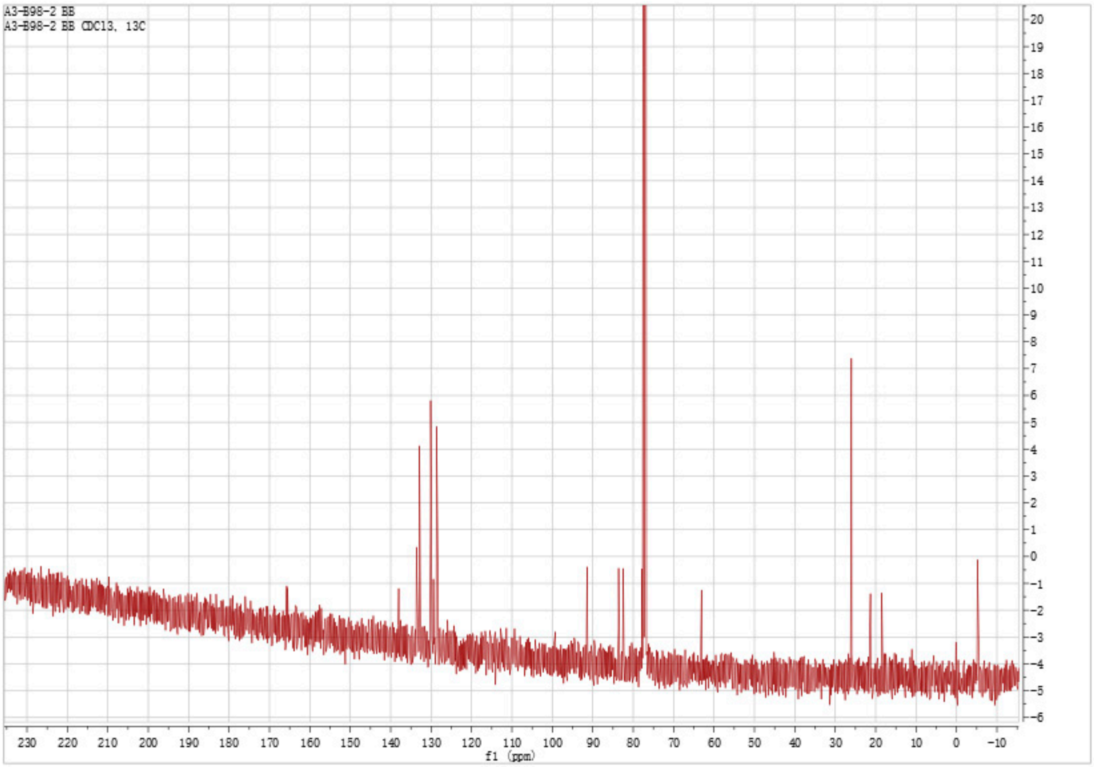

Figure s19. ^{13}C-NMR (CDCl3, 100 MHz) of 4

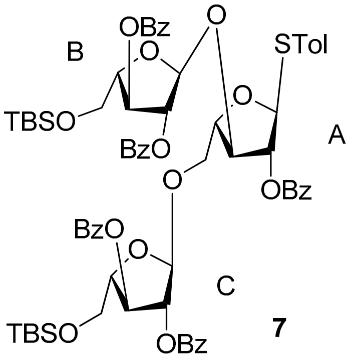

7

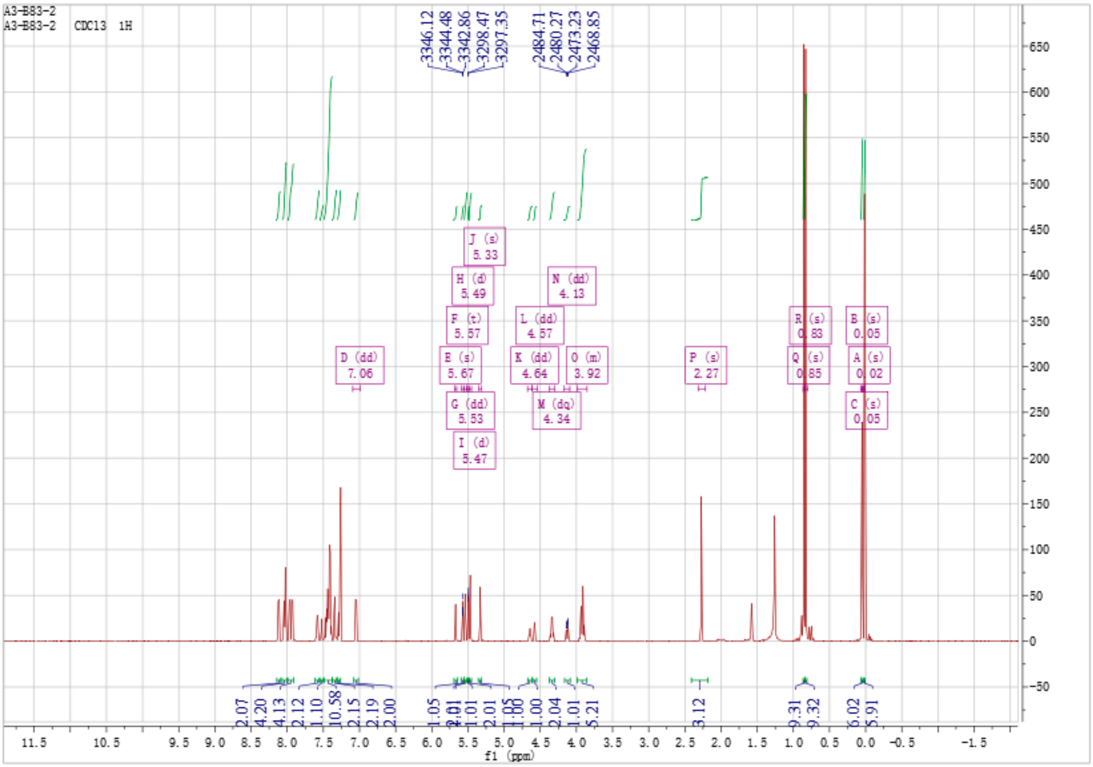

Figure S20. ^{1}H-NMR (CDCl3, 500 MHz) of 7

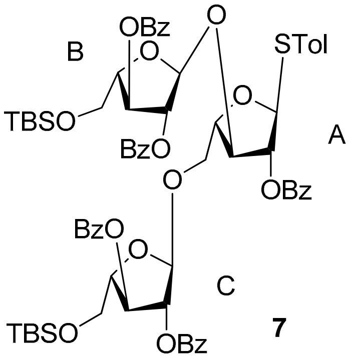

7

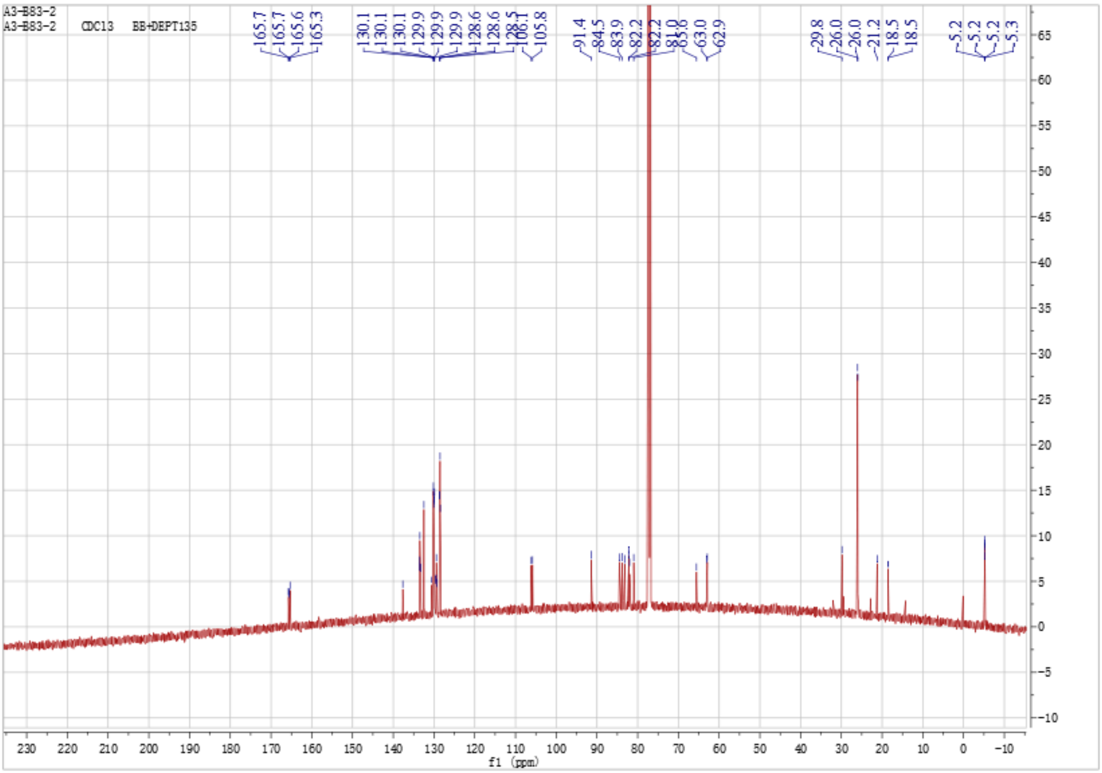

Figure S21. ^{13}C-NMR (CDCl3, 125 MHz) of 7

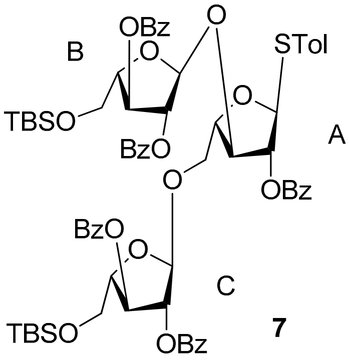

7

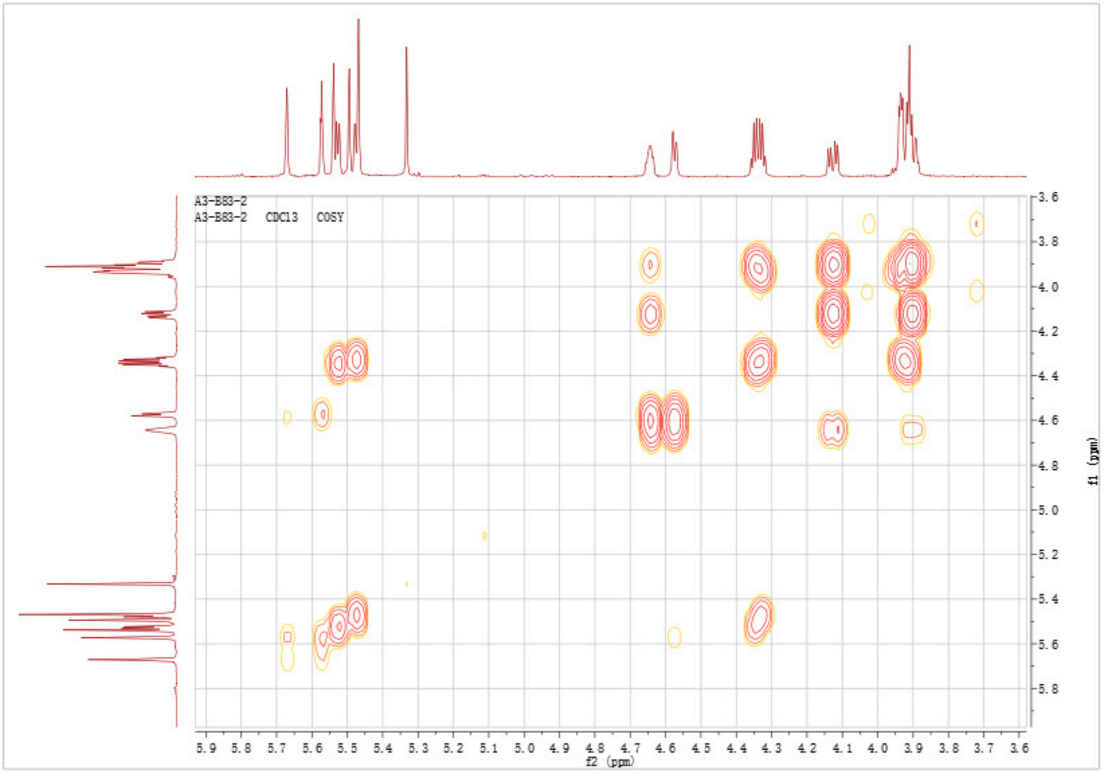

Figure S22. ^{1}H-^{1}H COSY of (CDCl3, 500 MHz) of 7

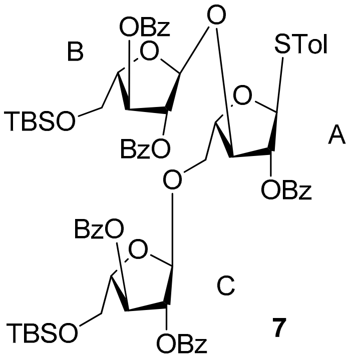

7

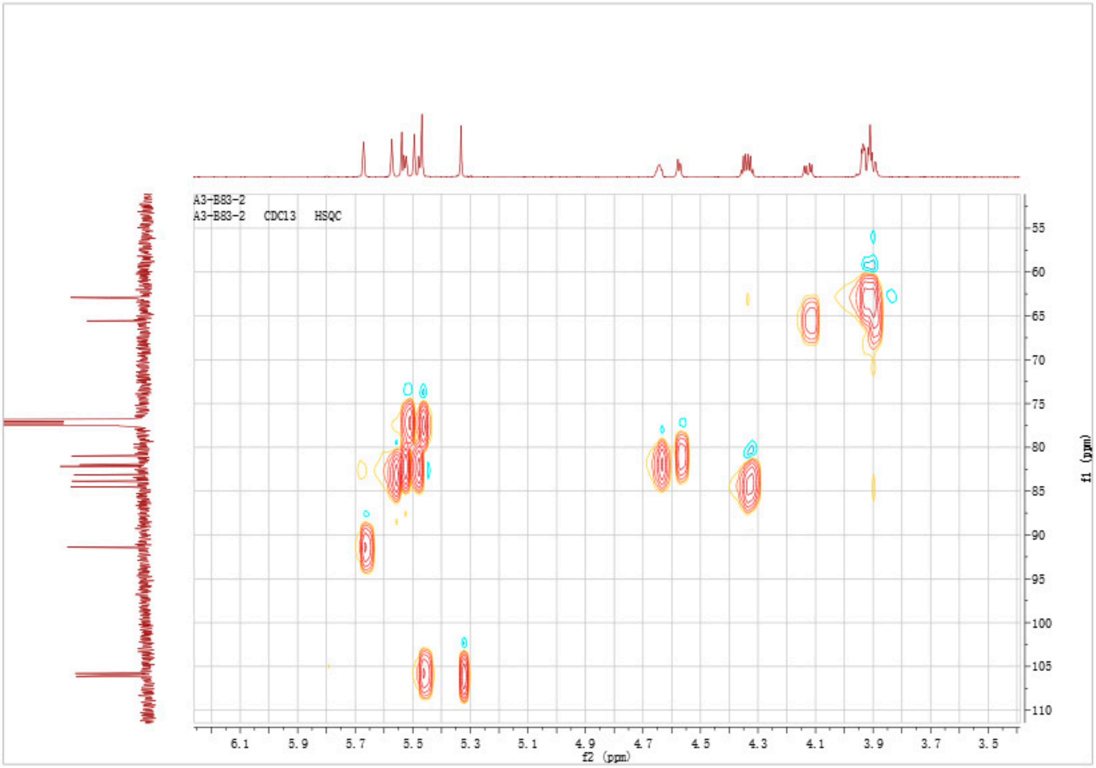

Figure S23. ^1H-^{13}C HSQC (CDCl3, 125 MHz) of 7

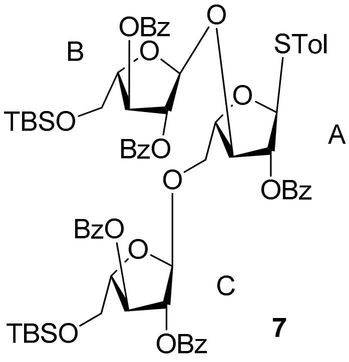

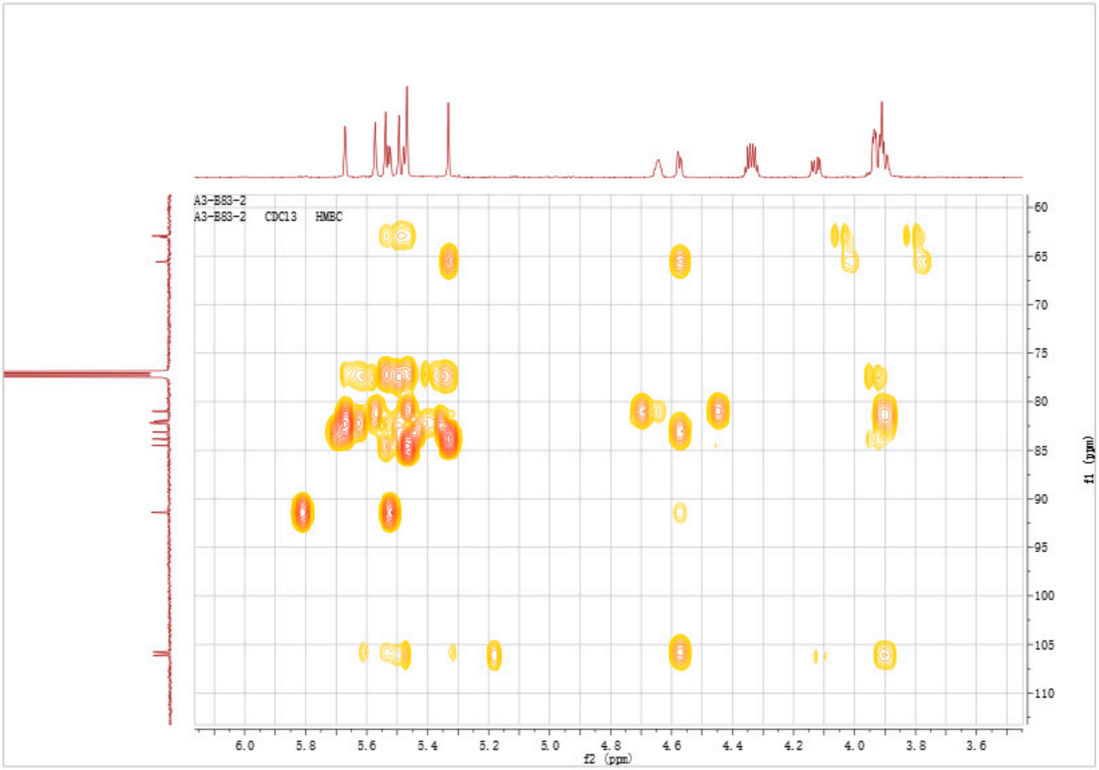

Figure S24. ^{1}H-^{13}C HMBC (CDCl3, 125 MHz) of 7

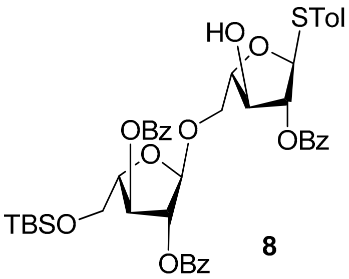

8

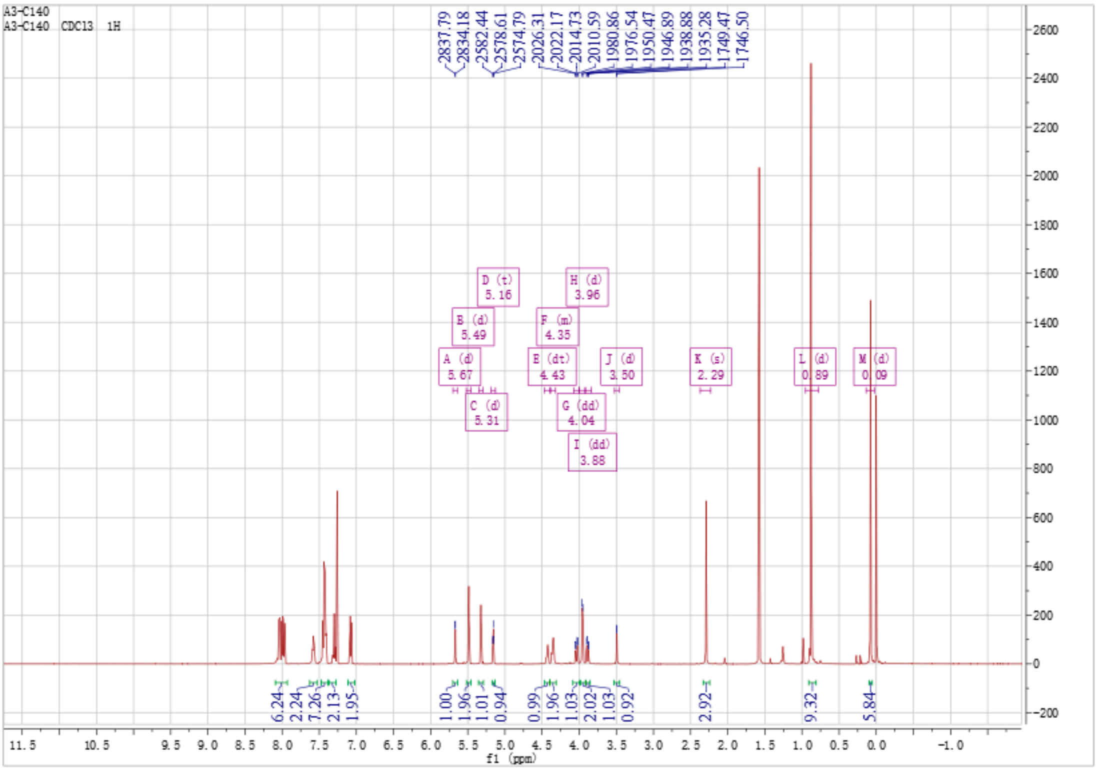

Figure S25. ^{1}H-NMR (CDCl3, 500 MHz) of 8

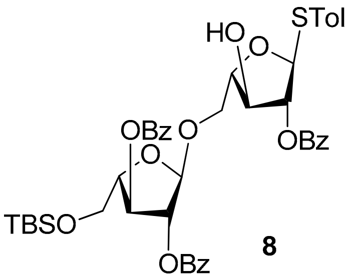

8

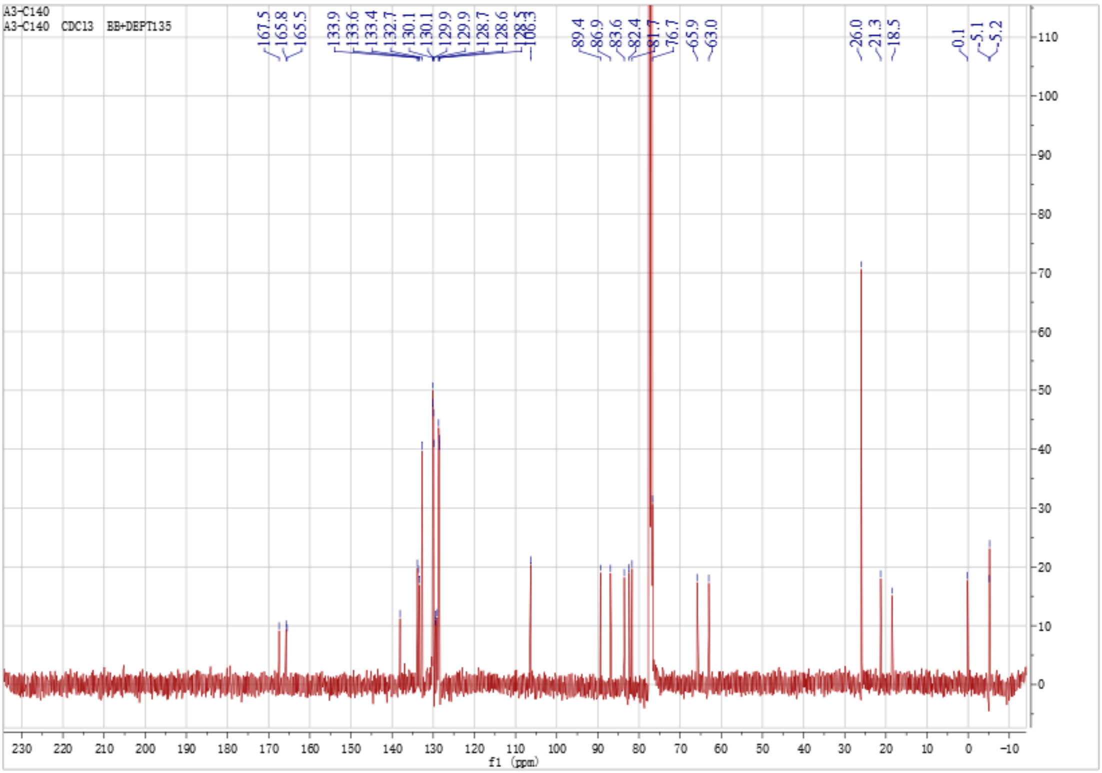

Figure S26. ^{13}C-NMR (CDCl3, 125 MHz) of 8

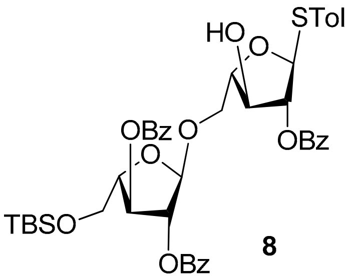

8

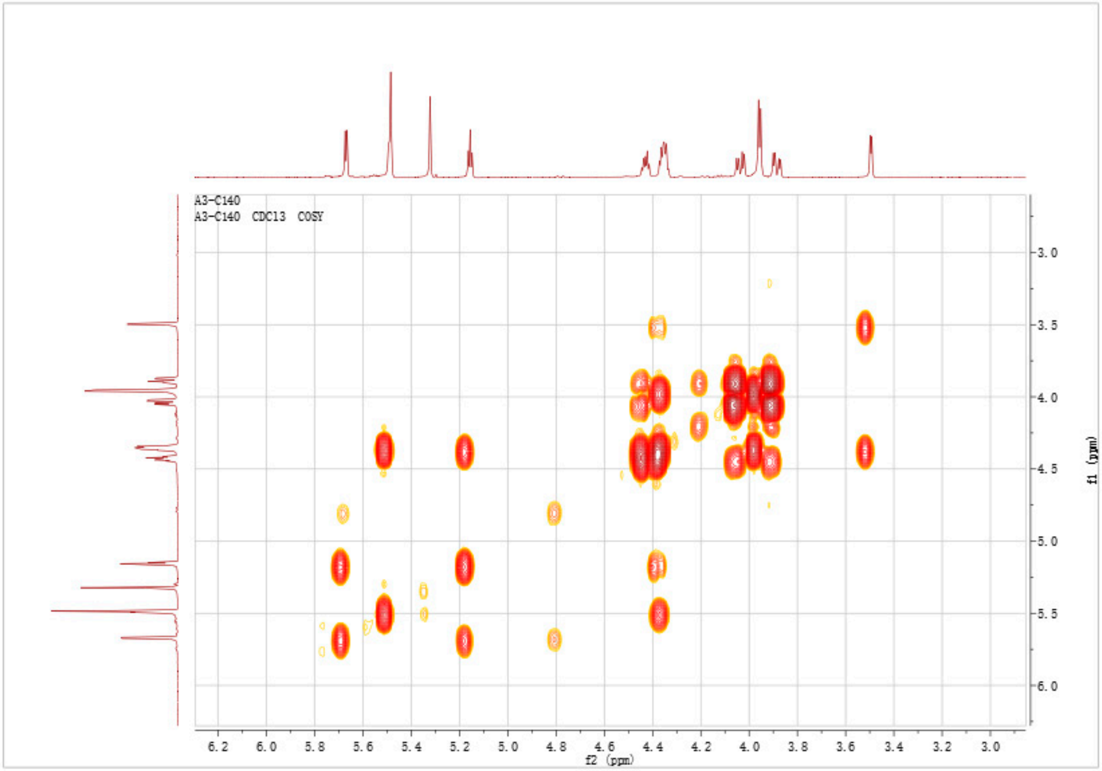

Figure.S27. ^{1}H-^{1}H COSY of (CDCl3, 500 MHz) of 8

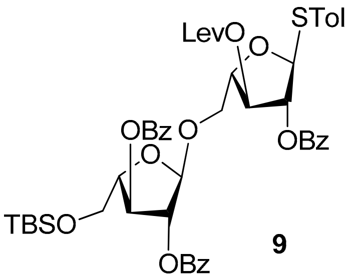

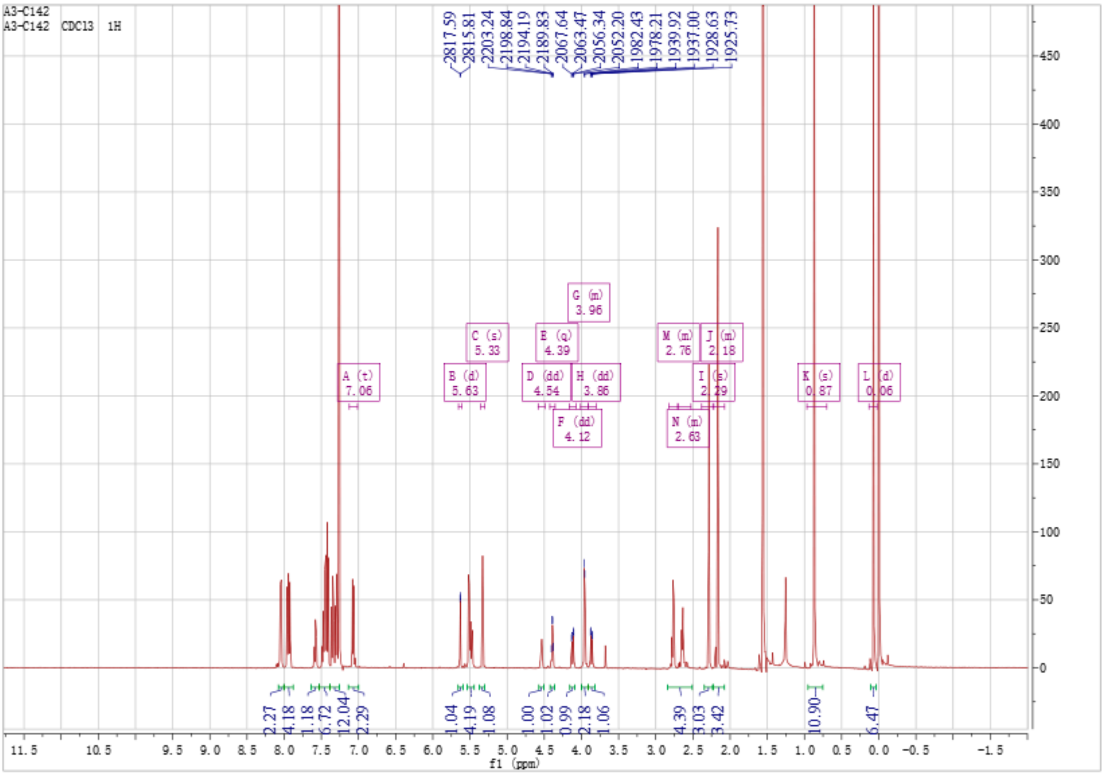

Figure.S28. ^{1}H-NMR (CDCl3, 400 MHz) of 9

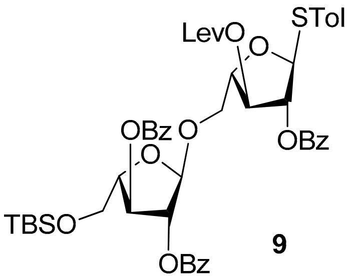

9

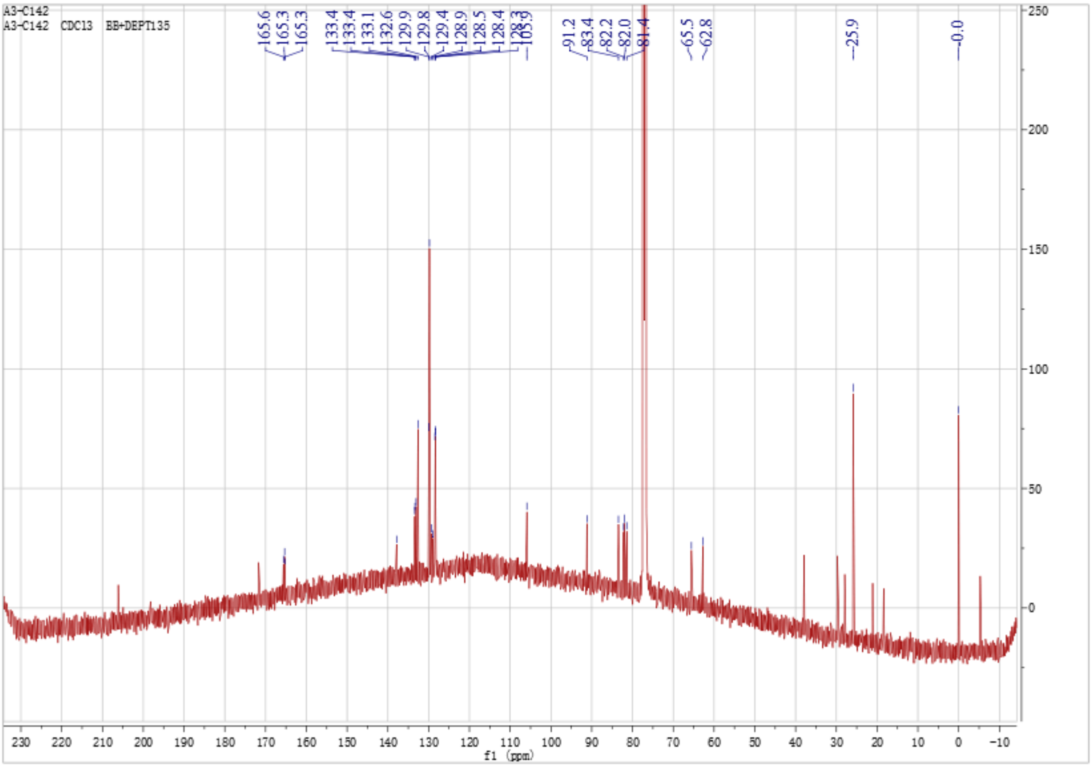

Figure S29. ^{13}C-NMR (CDCl3, 100 MHz) of 9

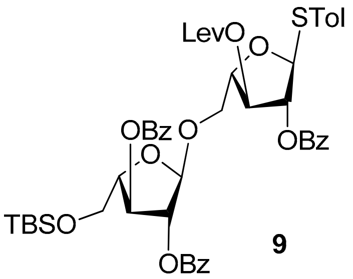

9

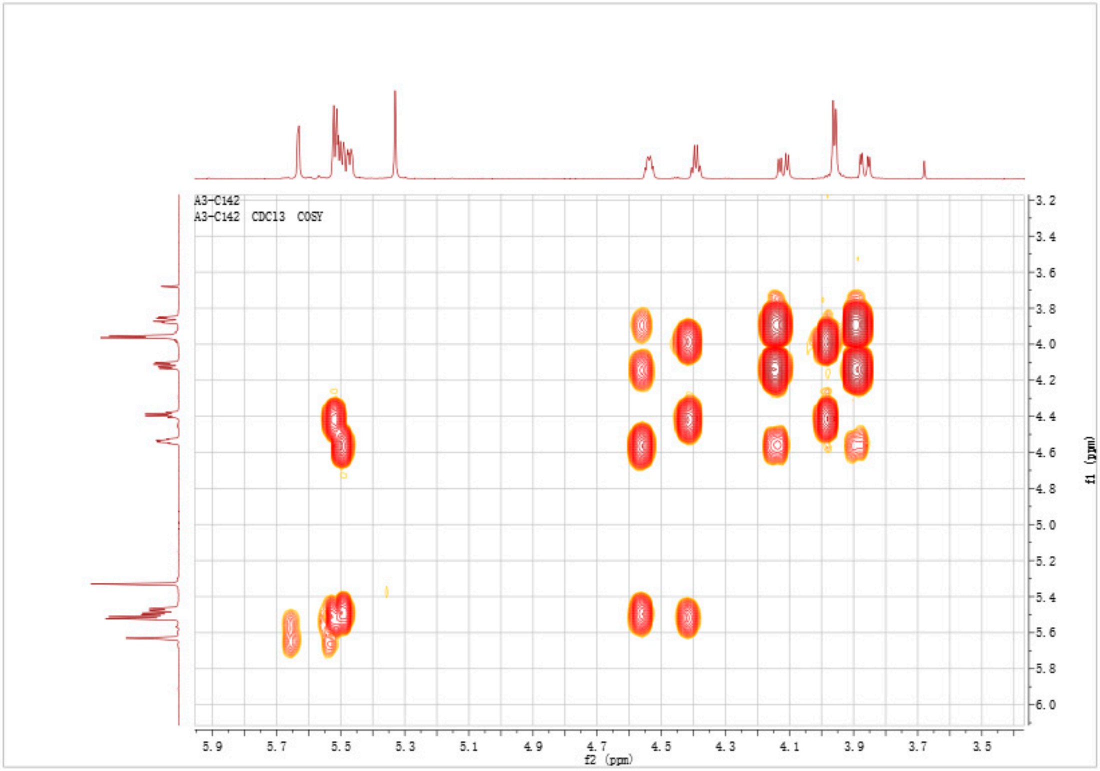

Figure S30. ^{1}H-^{1}H COSY of (CDCl3, 400 MHz) of 9

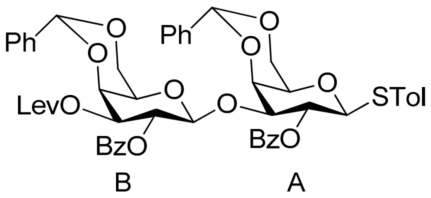

12

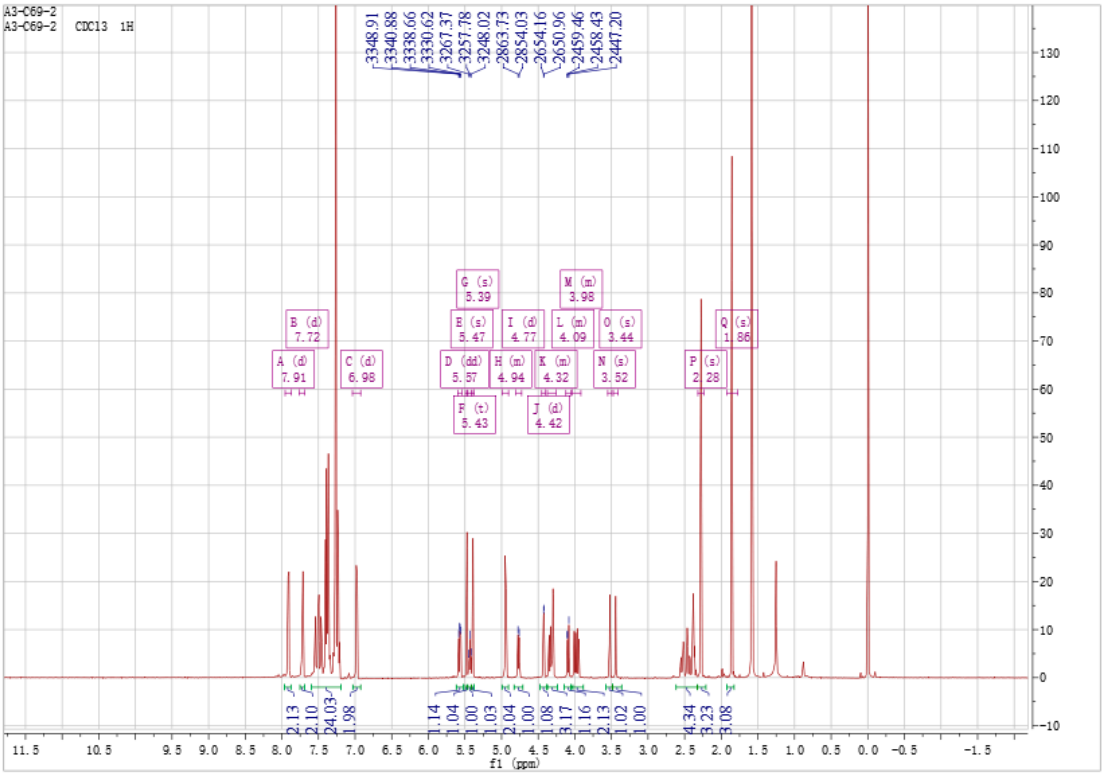

Figure S31. ^{1}H-NMR (CDCl3, 500 MHz) of 12

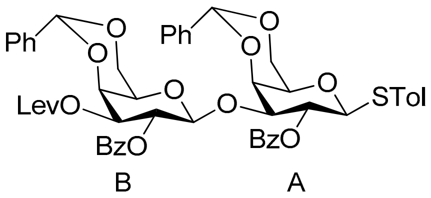

12

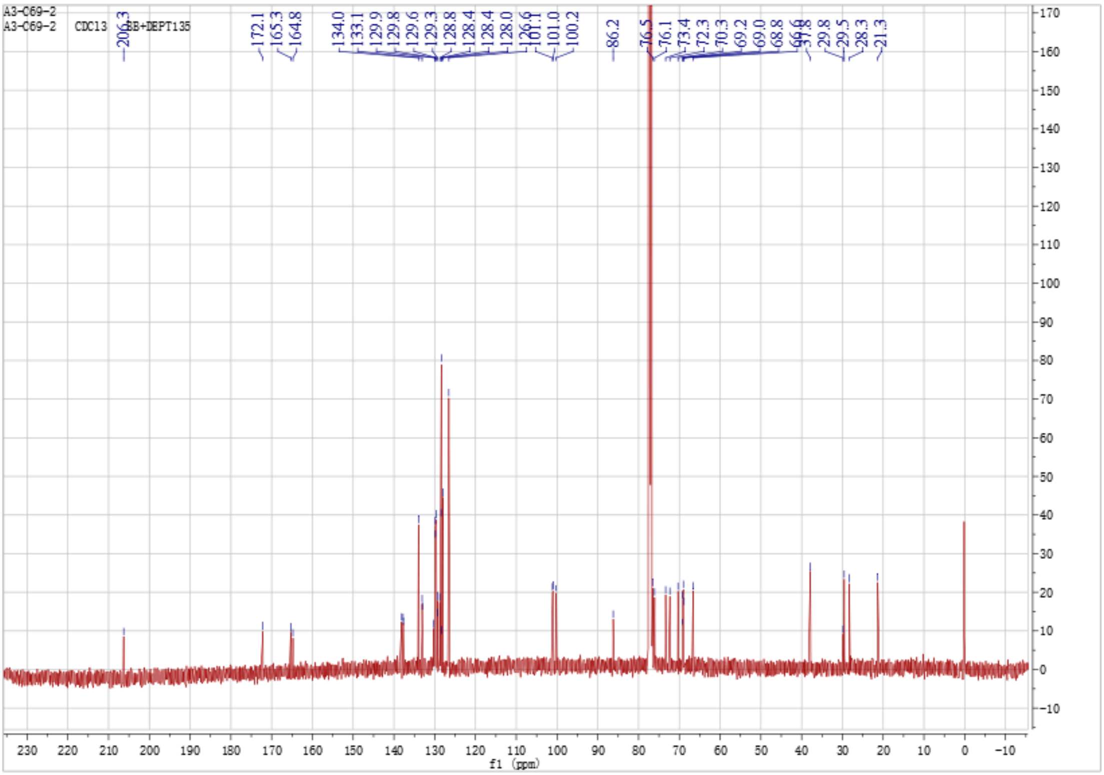

Figure S32. ^{13}C-NMR (CDCl3, 125 MHz) of 12

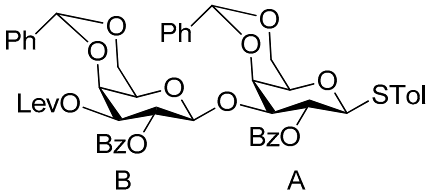

12

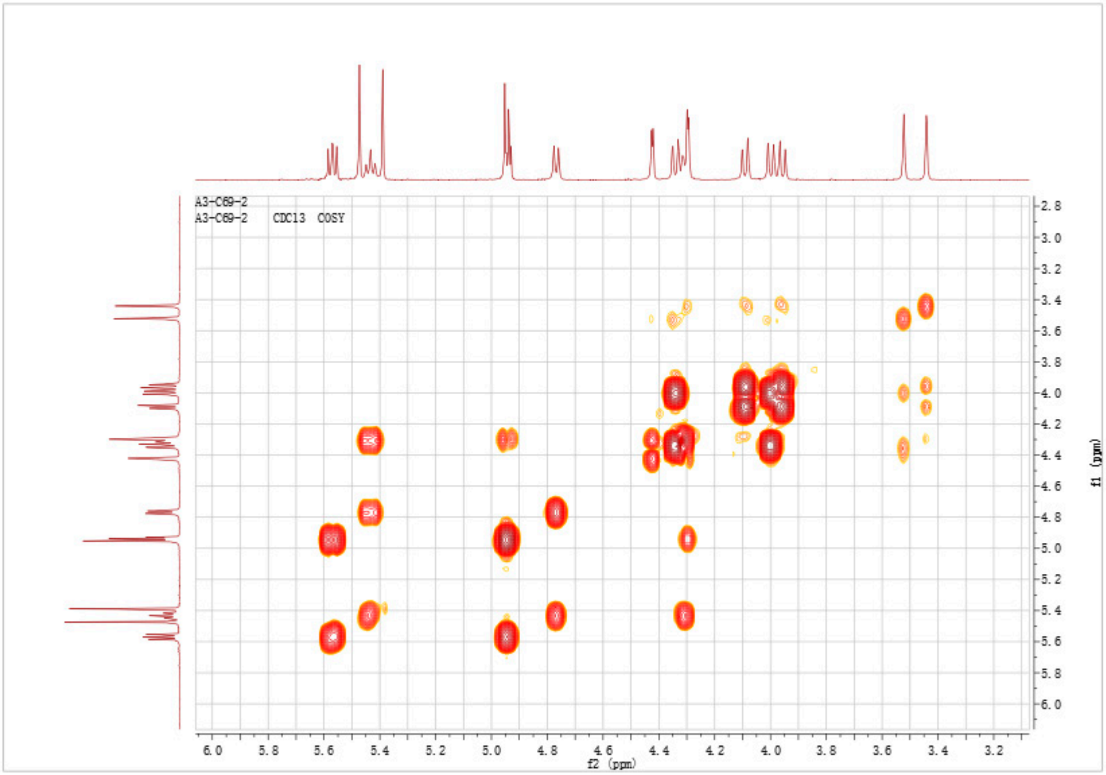

Figure S33. ^1H-^1H COSY of (CDCl3, 500 MHz) of 12

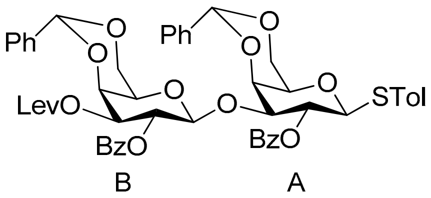

12

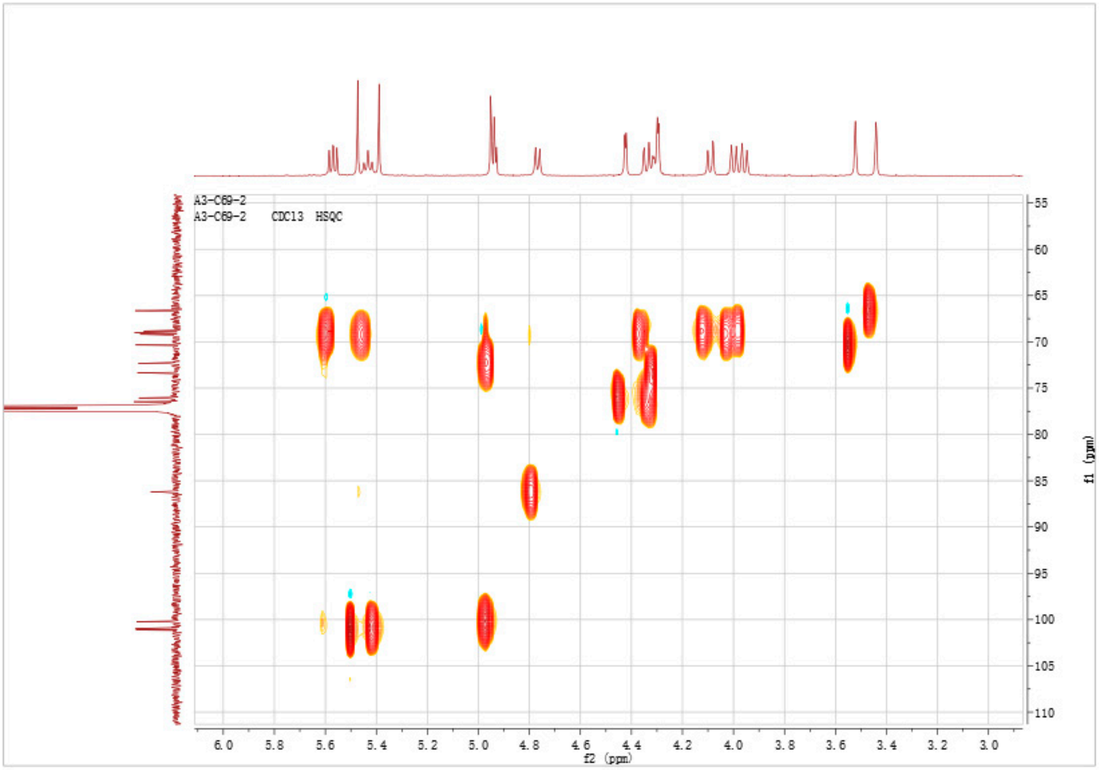

Figure S34. ^{1}H-^{13}C HSQC (CDCl3, 125 MHz) of 12

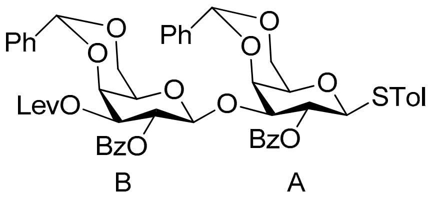

12

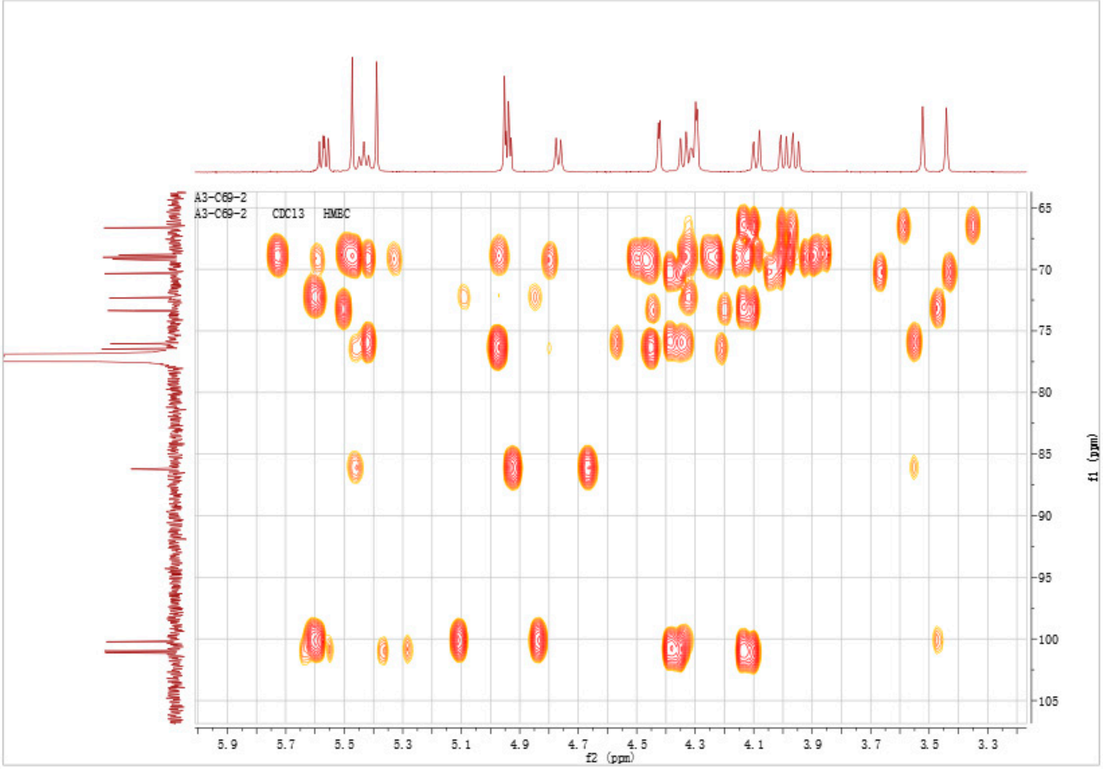

Figure S35. ^{1}H-^{13}C HMBC (CDCl3, 125 MHz) of 12

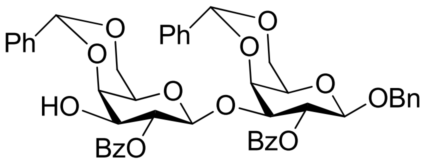

14

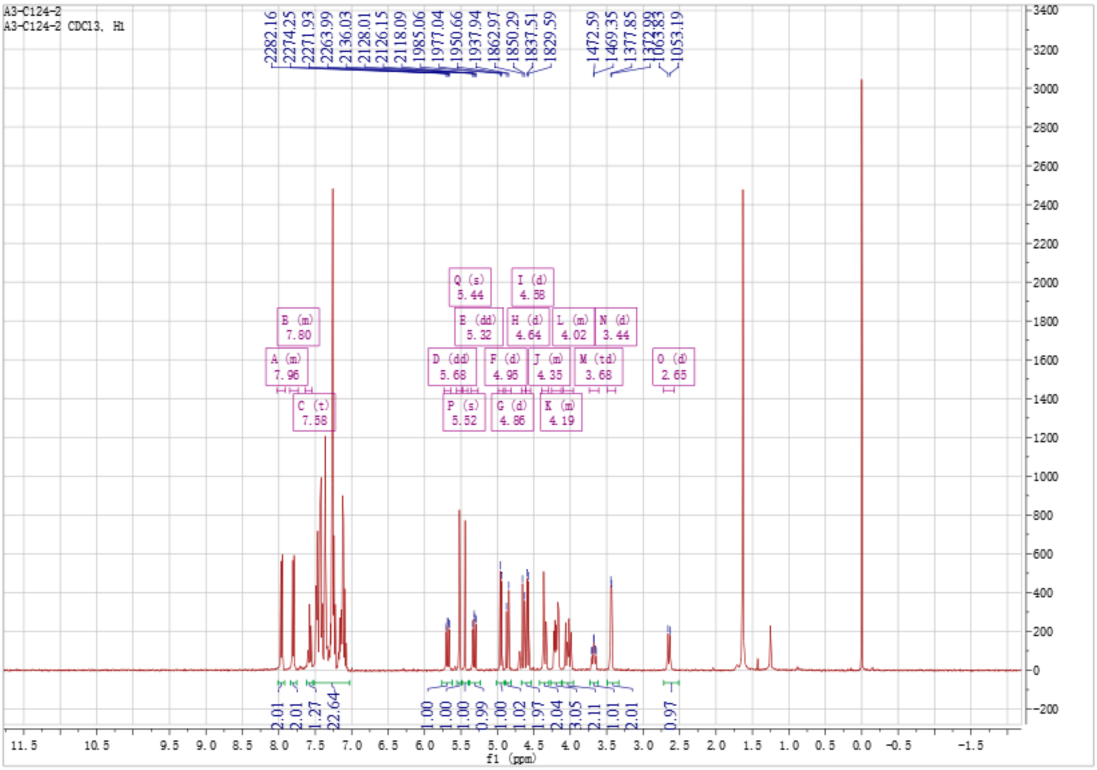

Figure S36. ^{1}H-NMR (CDCl3, 400 MHz) of 14

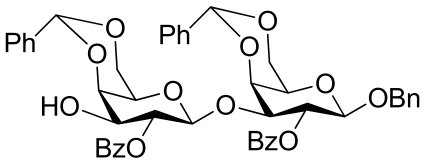

14

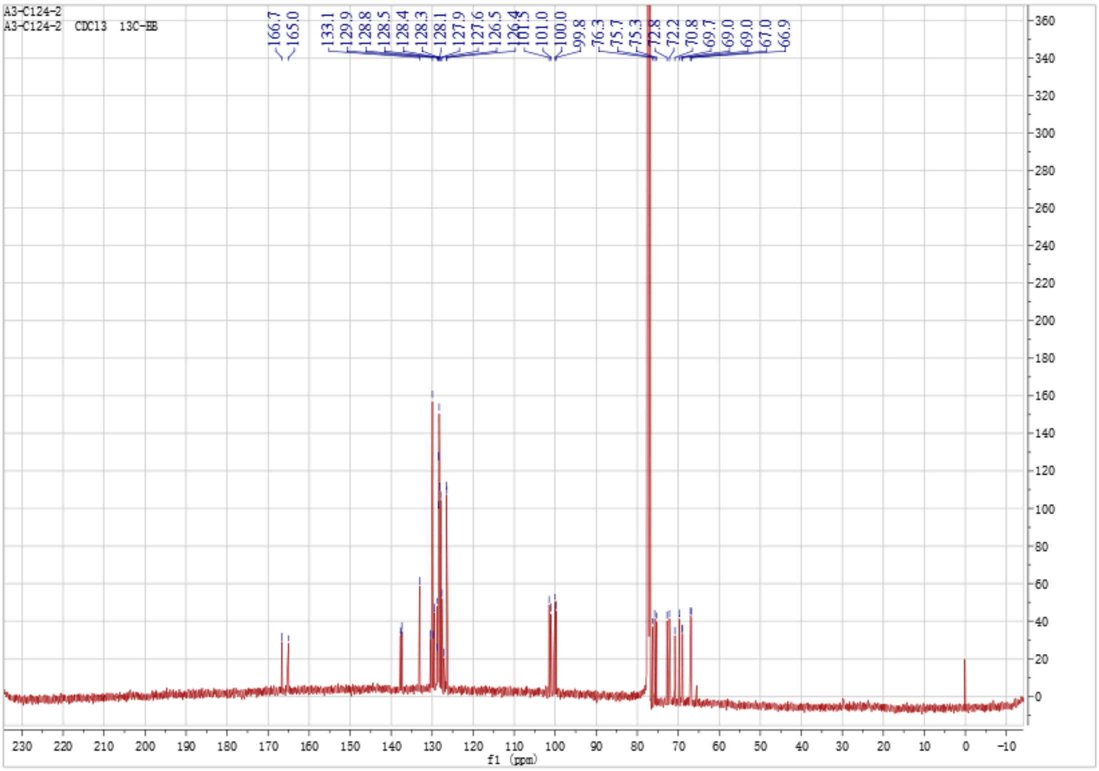

Figure S37. ^{13}C-NMR (CDCl3, 100 MHz) of 14

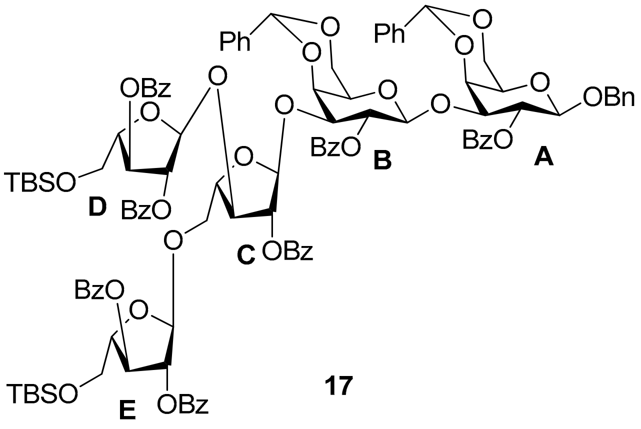

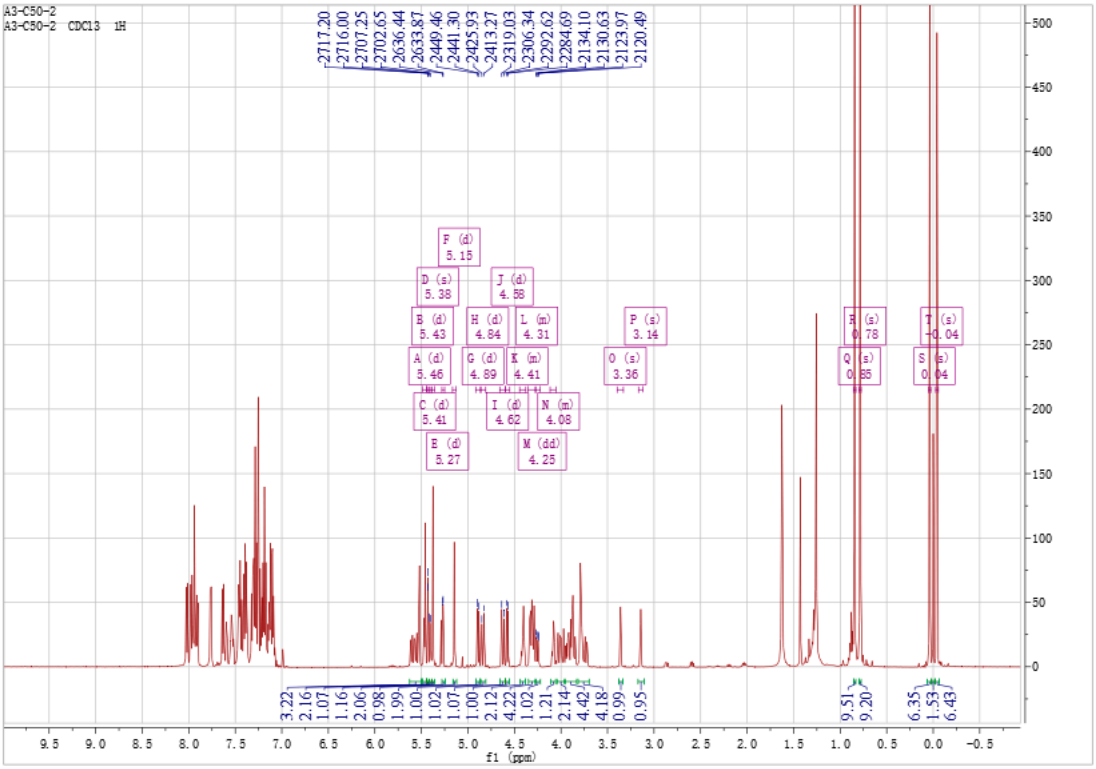

Figure S38. ¹H-NMR (CDCl3, 500 MHz) of 17

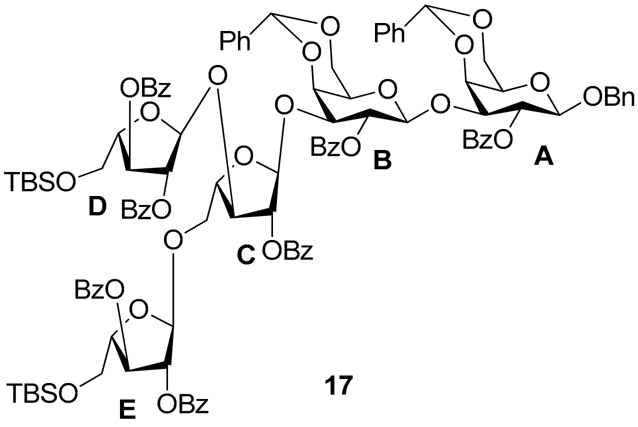

17

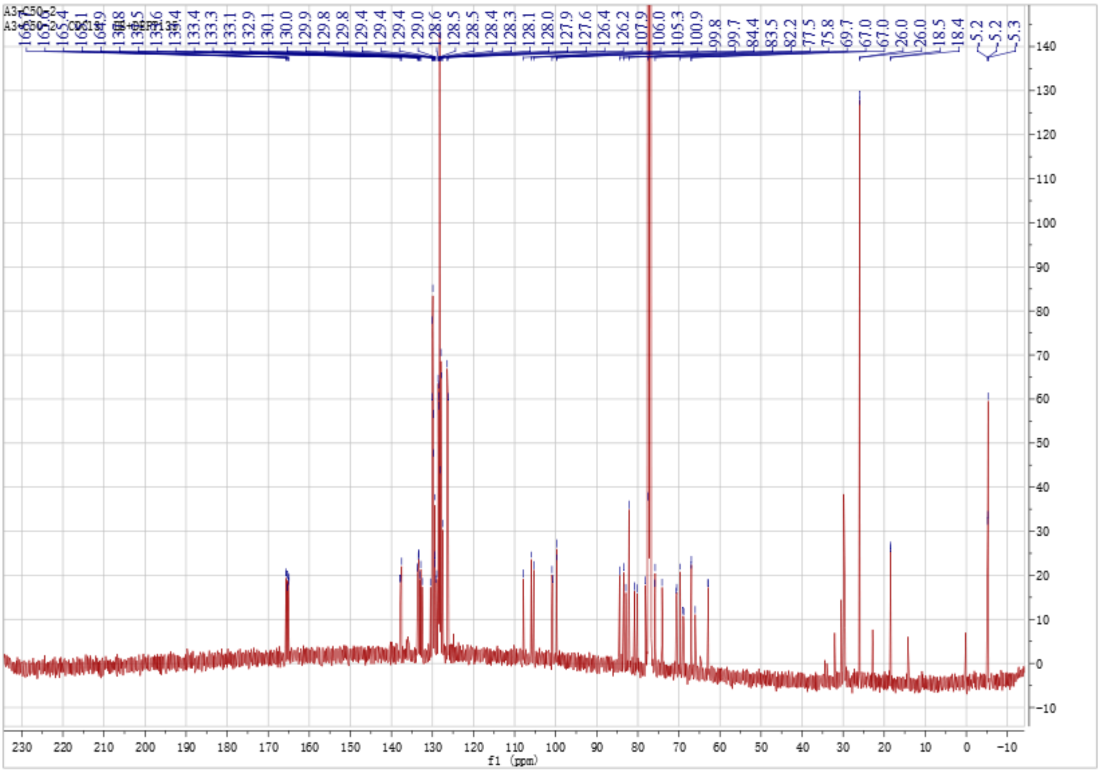

Figure S39. ^{13}C-NMR (CDCl3, 125 MHz) of 17

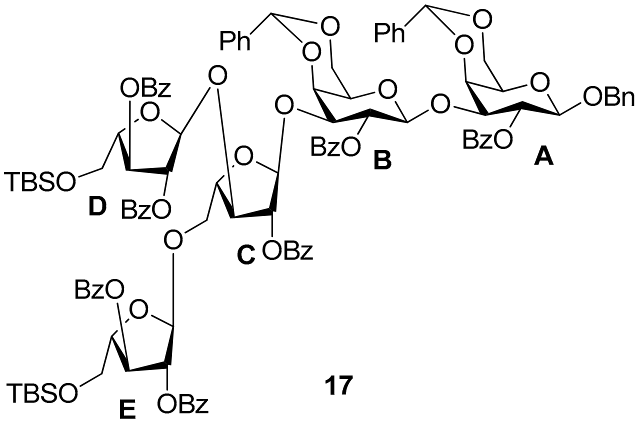

17

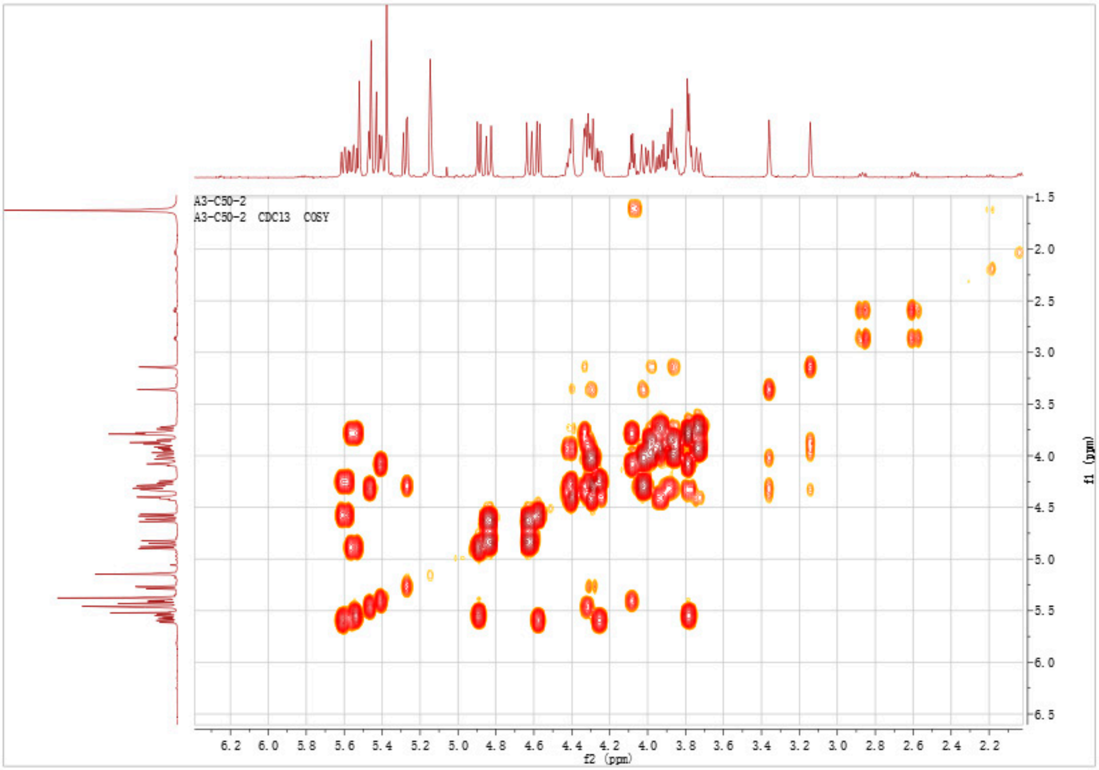

Figure S40. ^{1}H-^{1}H COSY of (CDCl3, 500 MHz) of 17

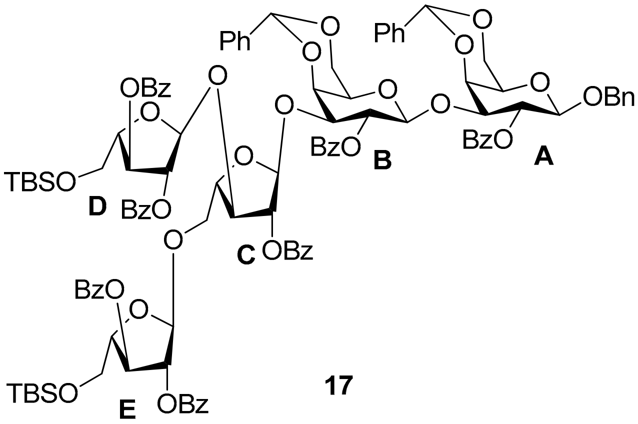

17

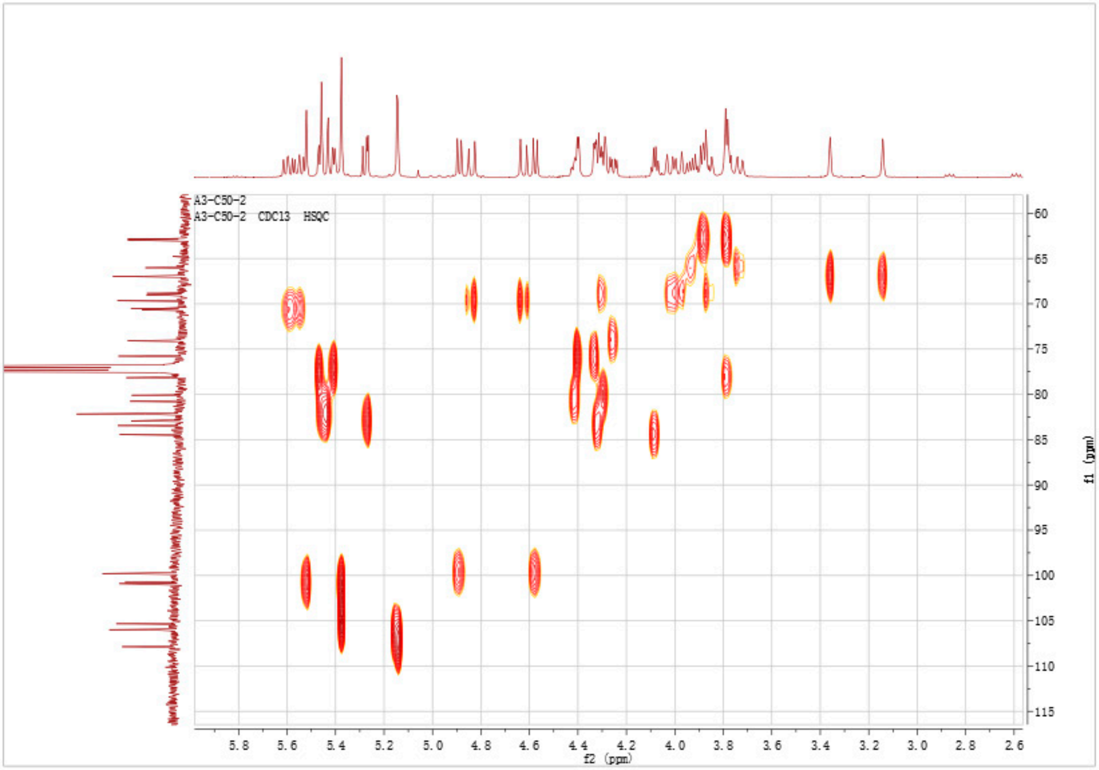

Figure S41. ^{1}H-^{13}C HSQC (CDCl3, 125 MHz) of 17

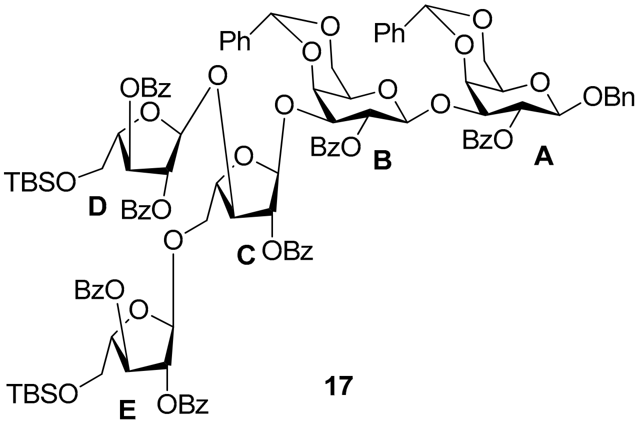

17

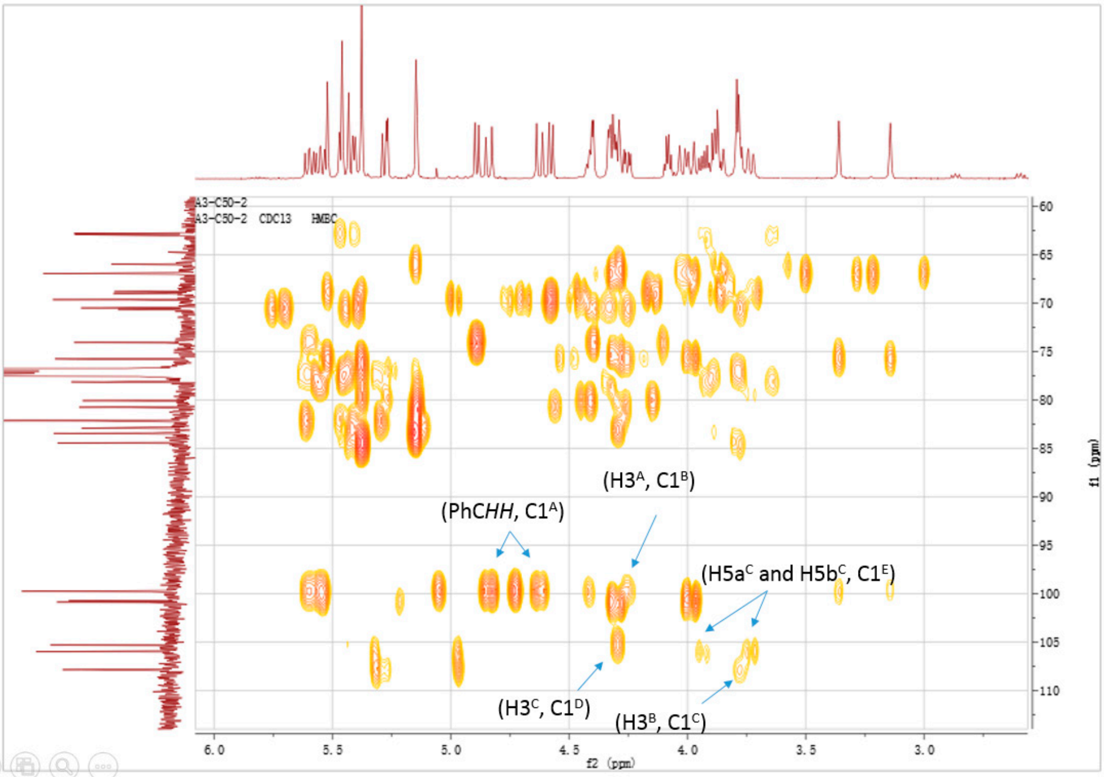

Figure S42. ^{1}H-^{13}C HMBC (CDCl3, 125 MHz) of 17

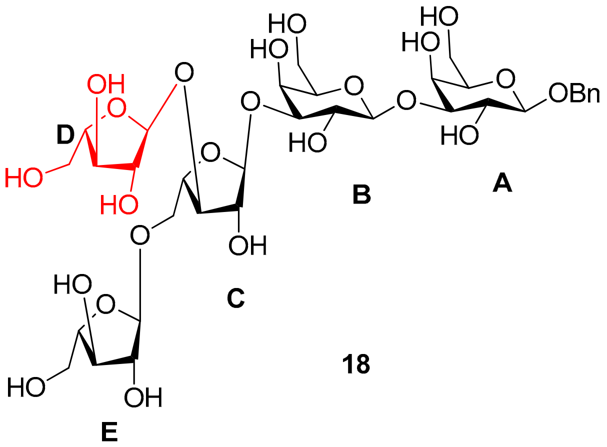

18

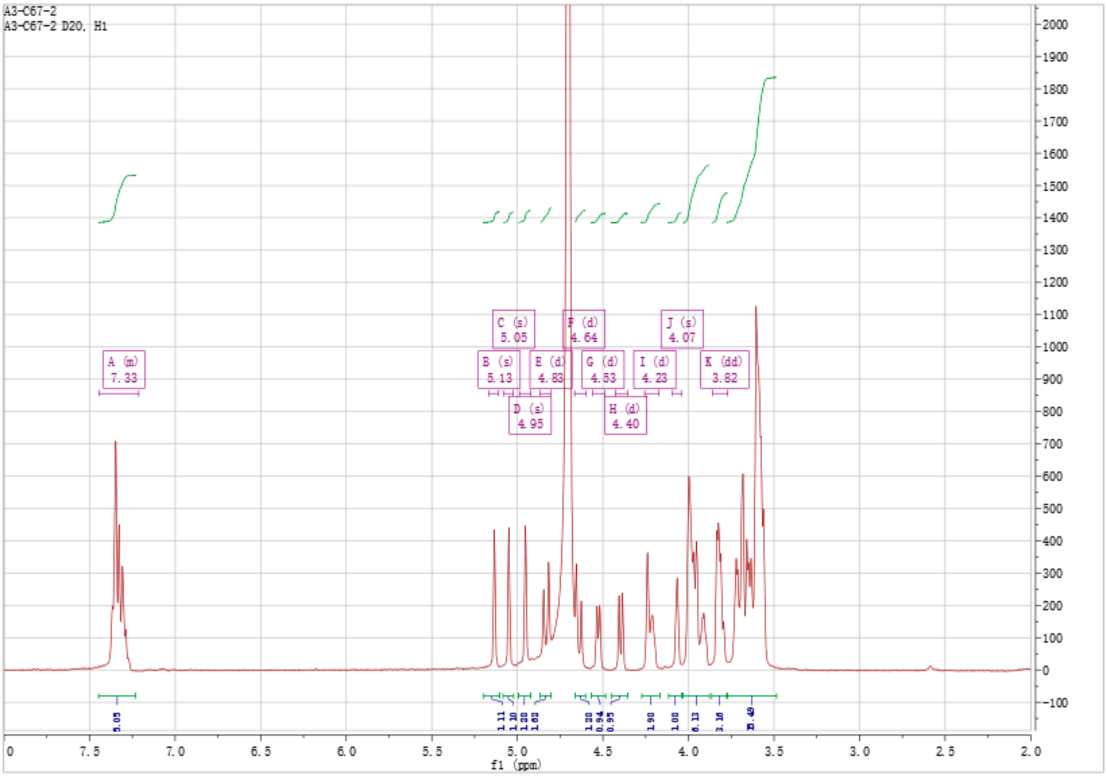

Figure S43. ^{1}H-NMR (CDCl3, 500 MHz) of 18

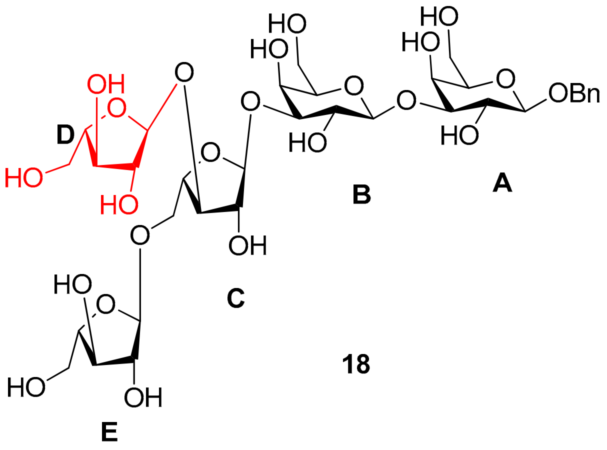

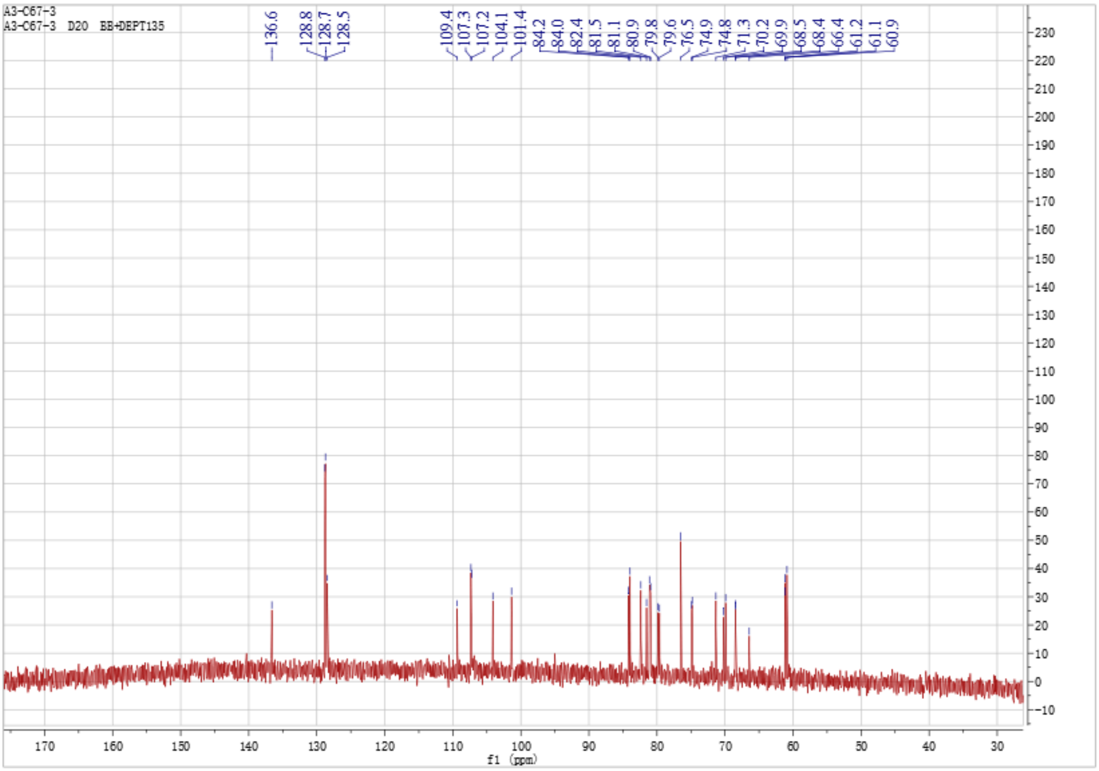

Figure S44. ^{13}C-NMR (CDCl3, 125 MHz) of 18

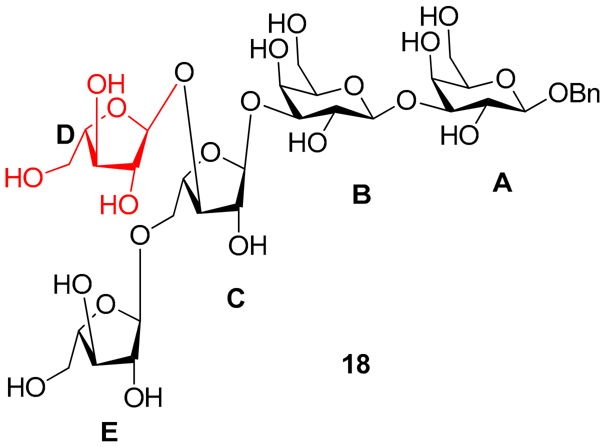

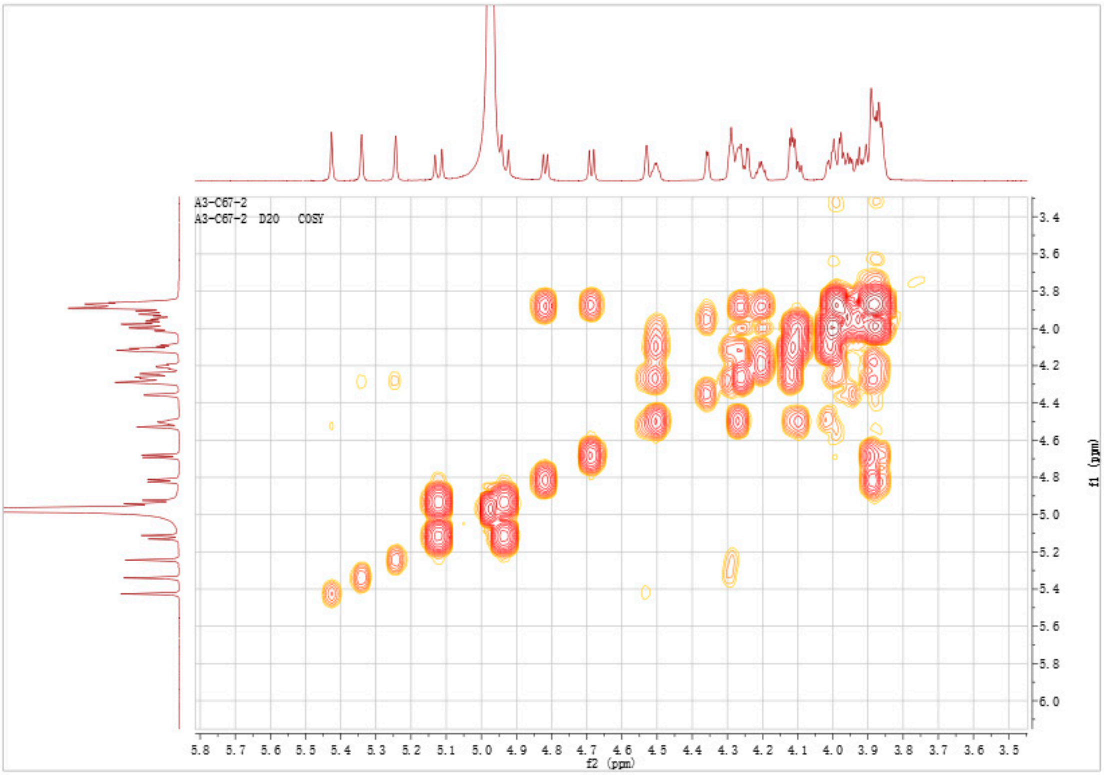

Figure S45. ^{1}H-^{1}H COSY of (CDCl3, 500 MHz) of 18

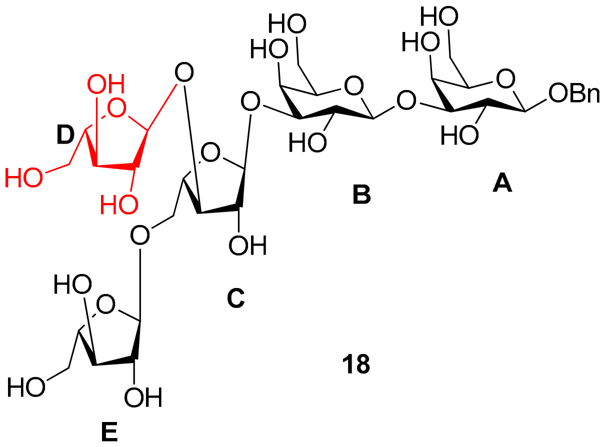

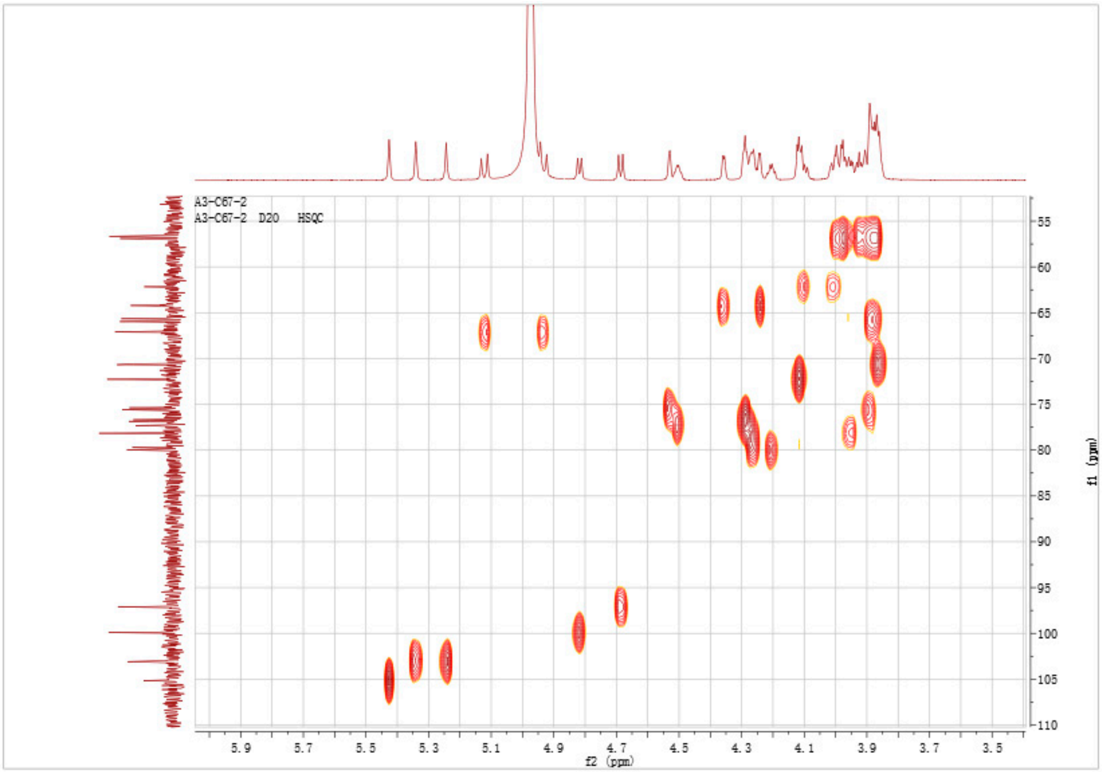

Figure S46. ^{1}H-^{13}C HSQC (CDCl3, 125 MHz) of 18

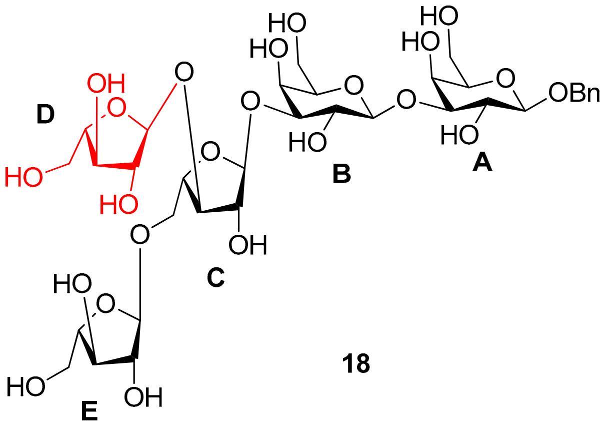

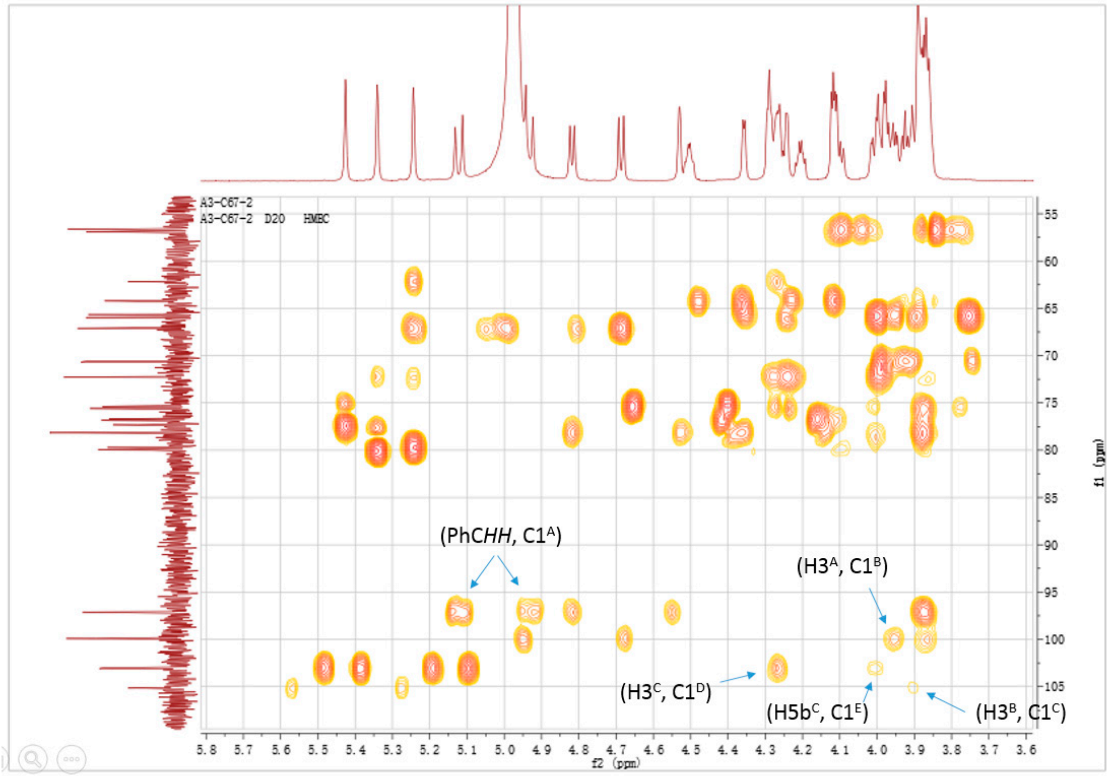

Figure S47. ^{1}H-^{13}C HMBC (CDCl3, 125 MHz) of 18

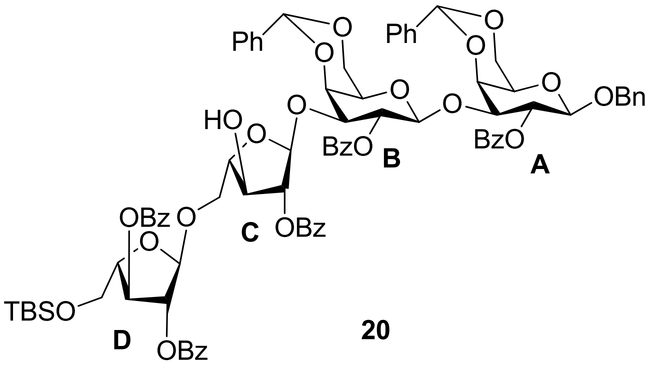

20

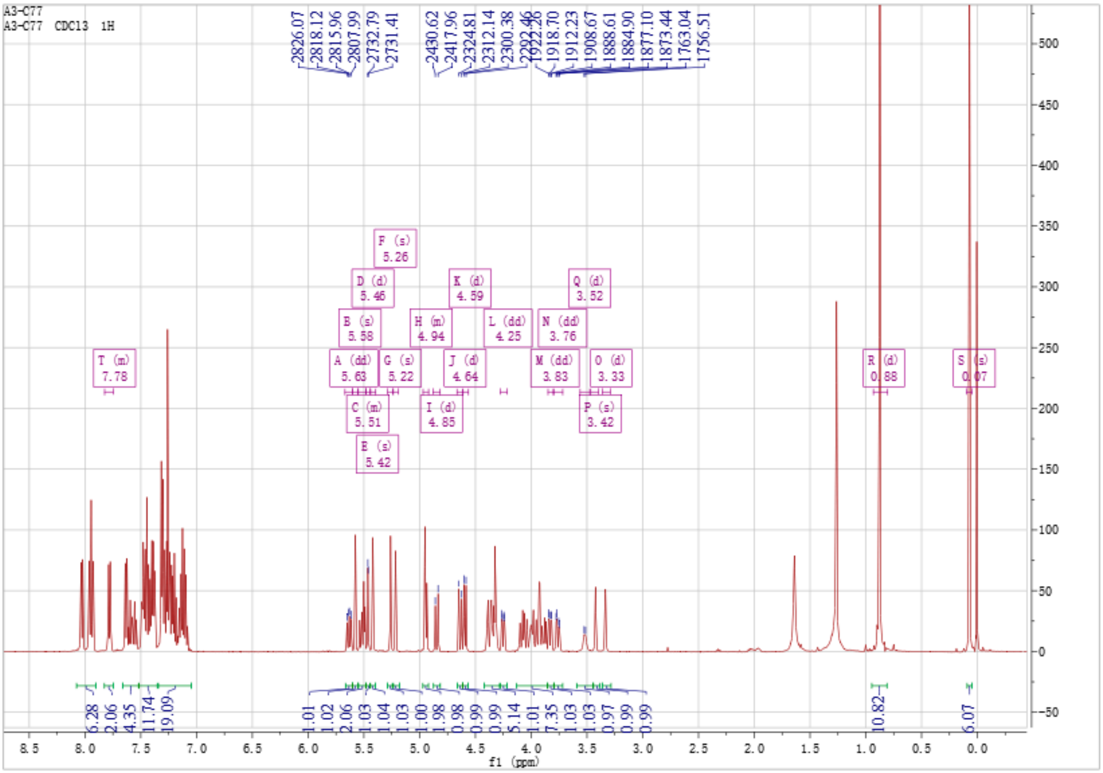

Figure S48. ¹H-NMR (CDCl3, 500 MHz) of 20

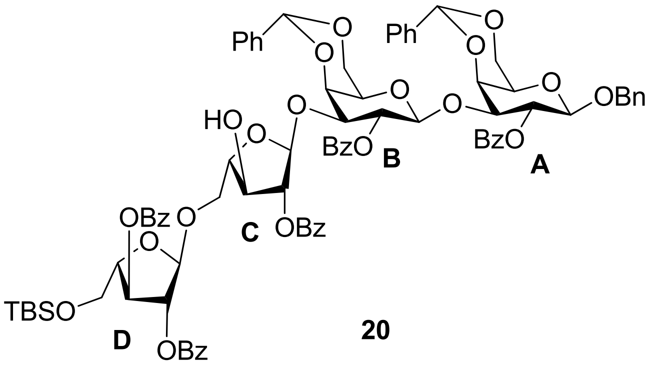

20

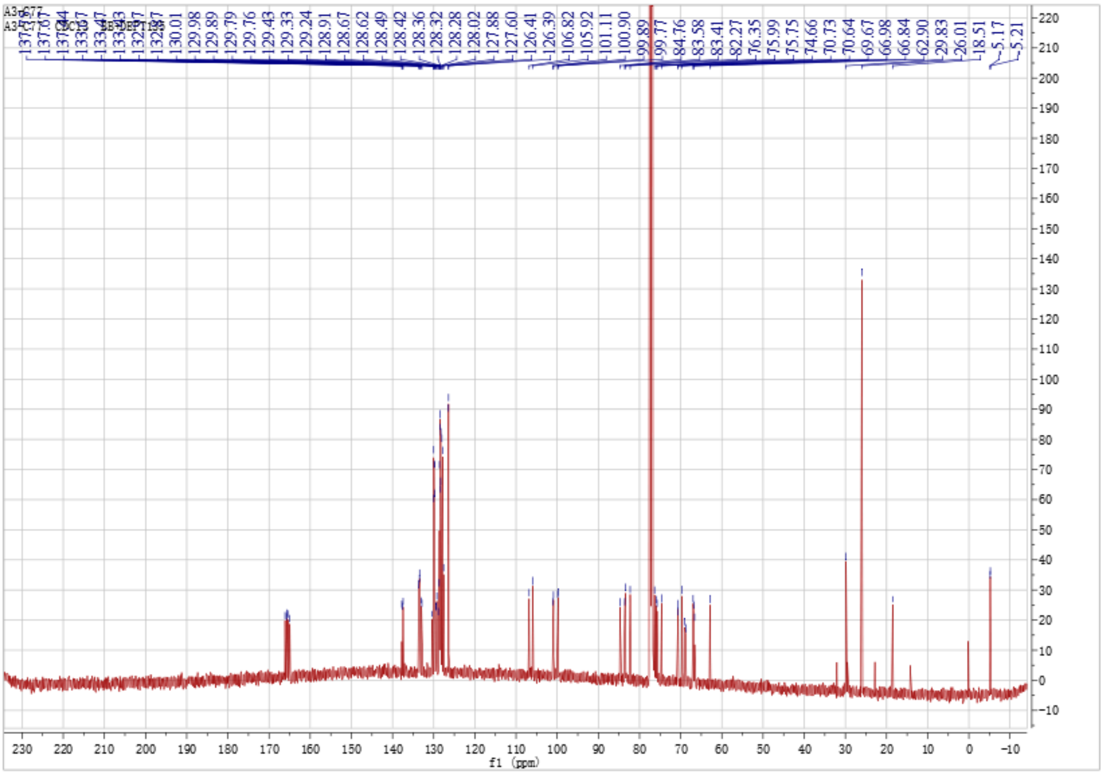

Figure S49. ^{13}C-NMR (CDCl3, 125 MHz) of 20

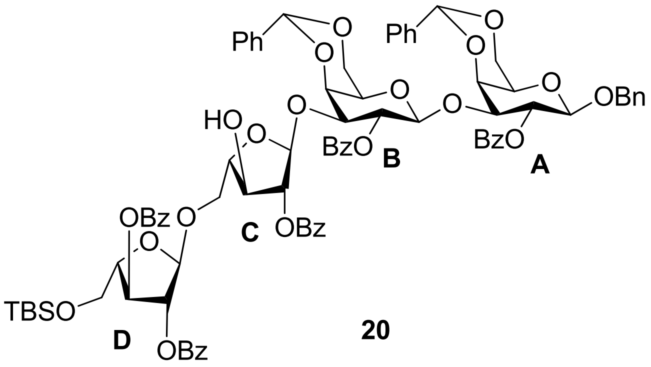

20

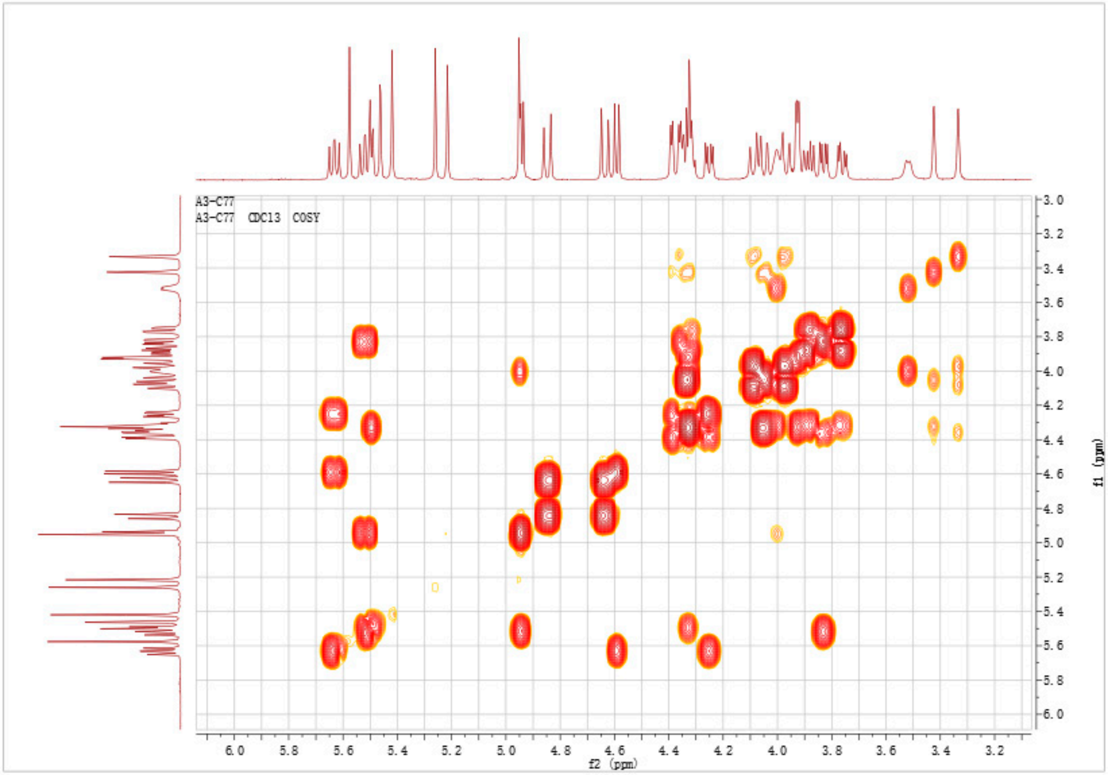

Figure S50. ^{1}H-^{1}H COSY of (CDCl3, 500 MHz) of 20

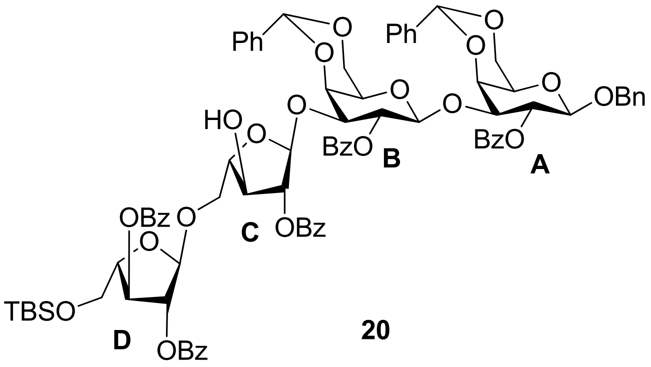

20

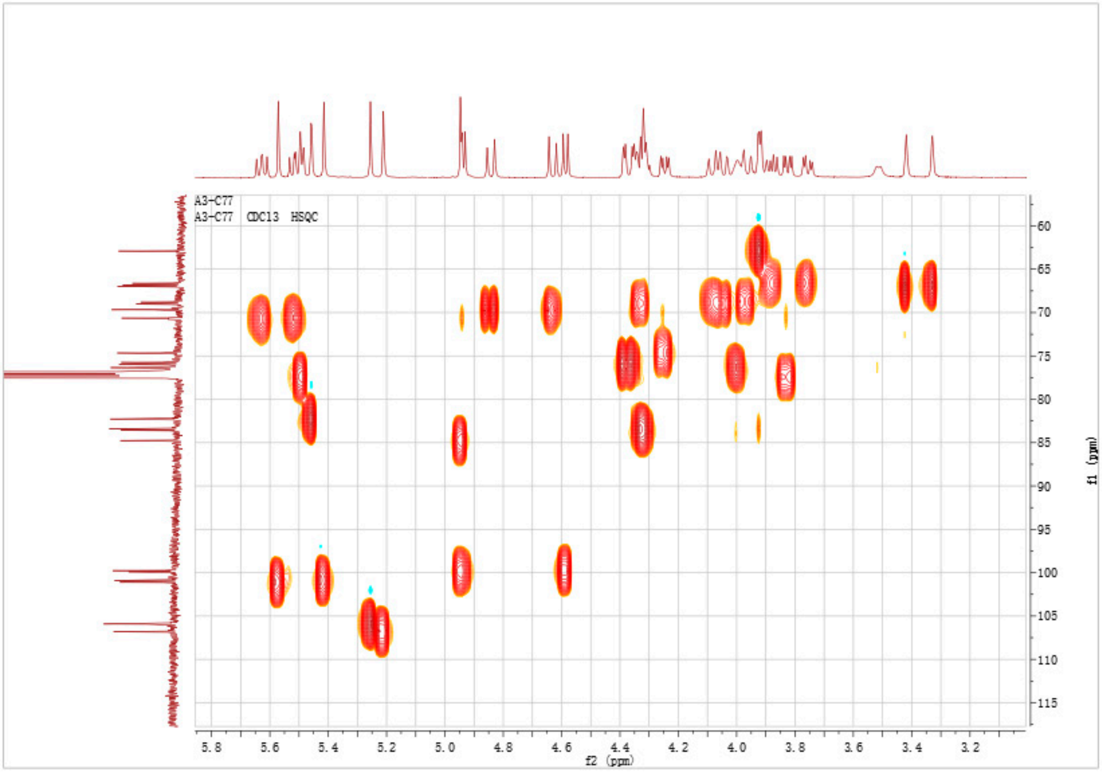

Figure S51. ^{1}H-^{13}C HSQC (CDCl3, 125 MHz) of 20

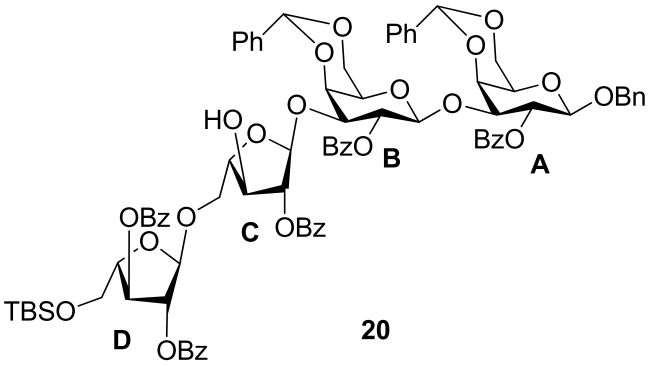

20

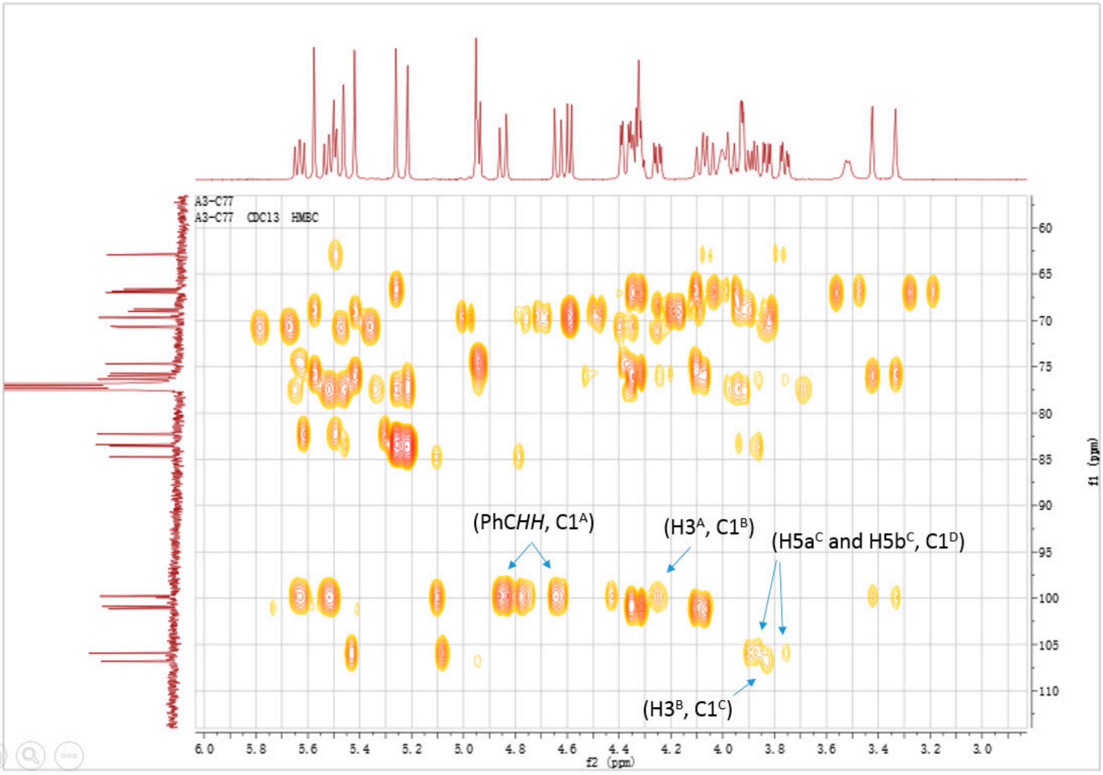

Figure S52. ^{1}H-^{13}C HMBC (CDCl3, 125 MHz) of 20

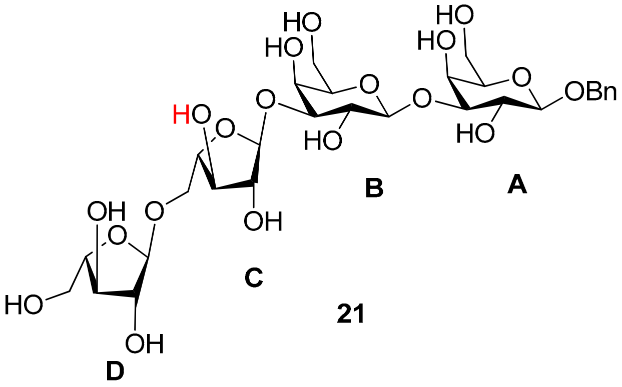

21

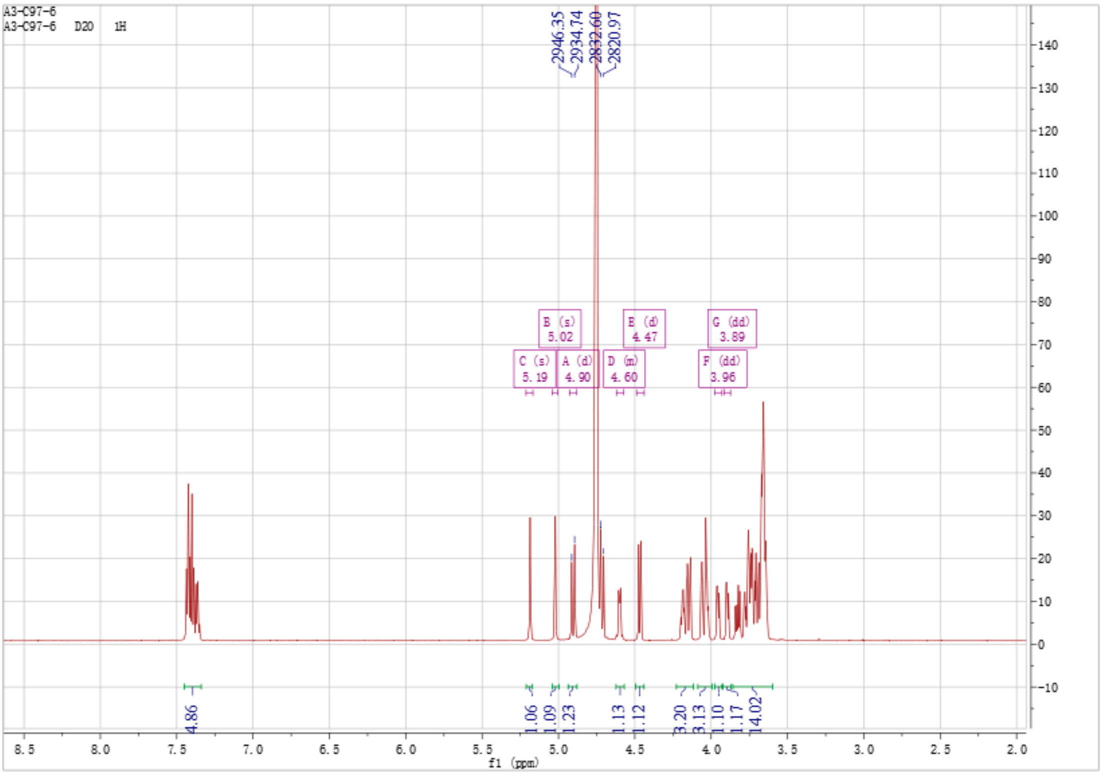

Figure S53. ^{1}H-NMR (CDCl3, 500 MHz) of 21

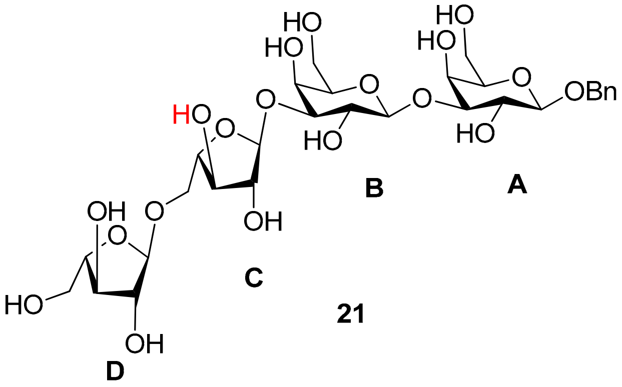

21

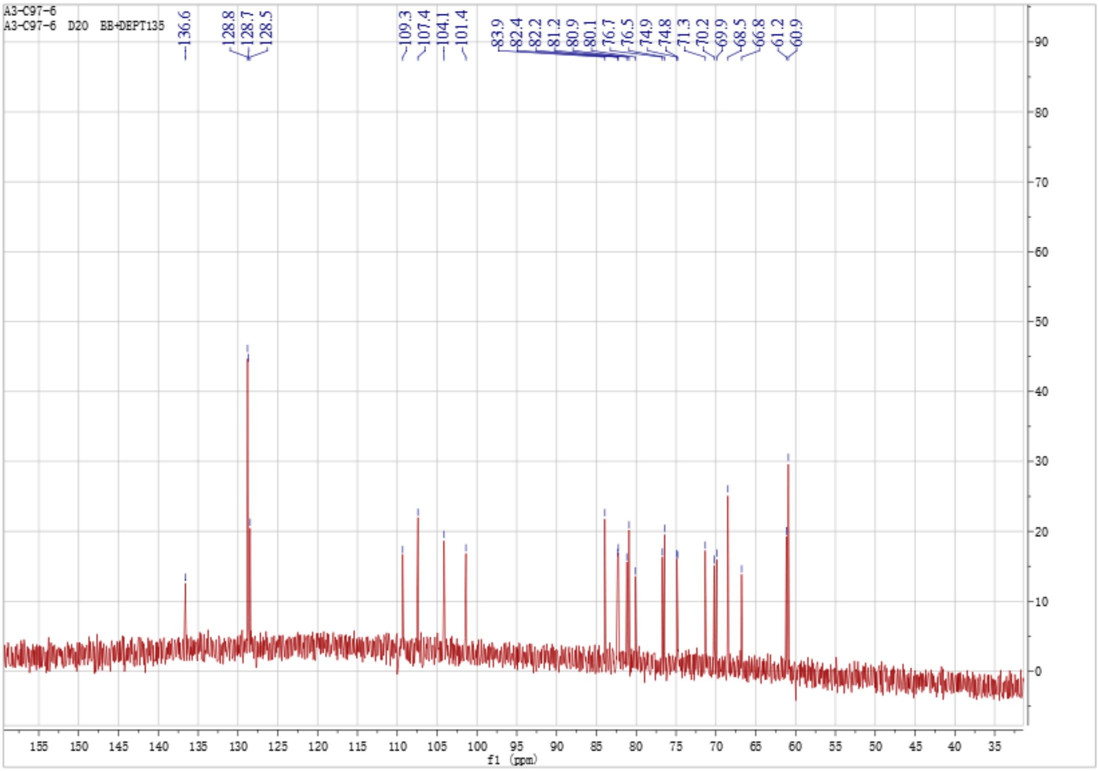

Figure S54. ^{13}C-NMR (CDCl3, 125 MHz) of 21

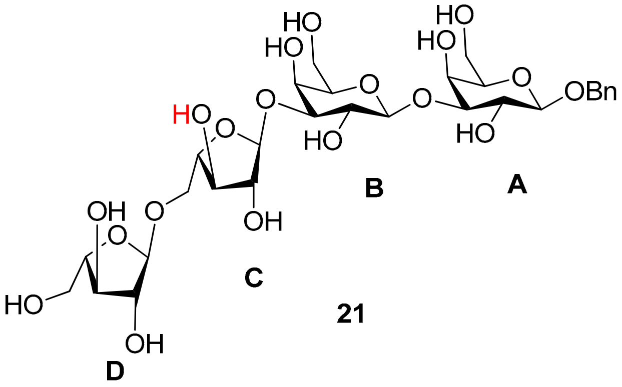

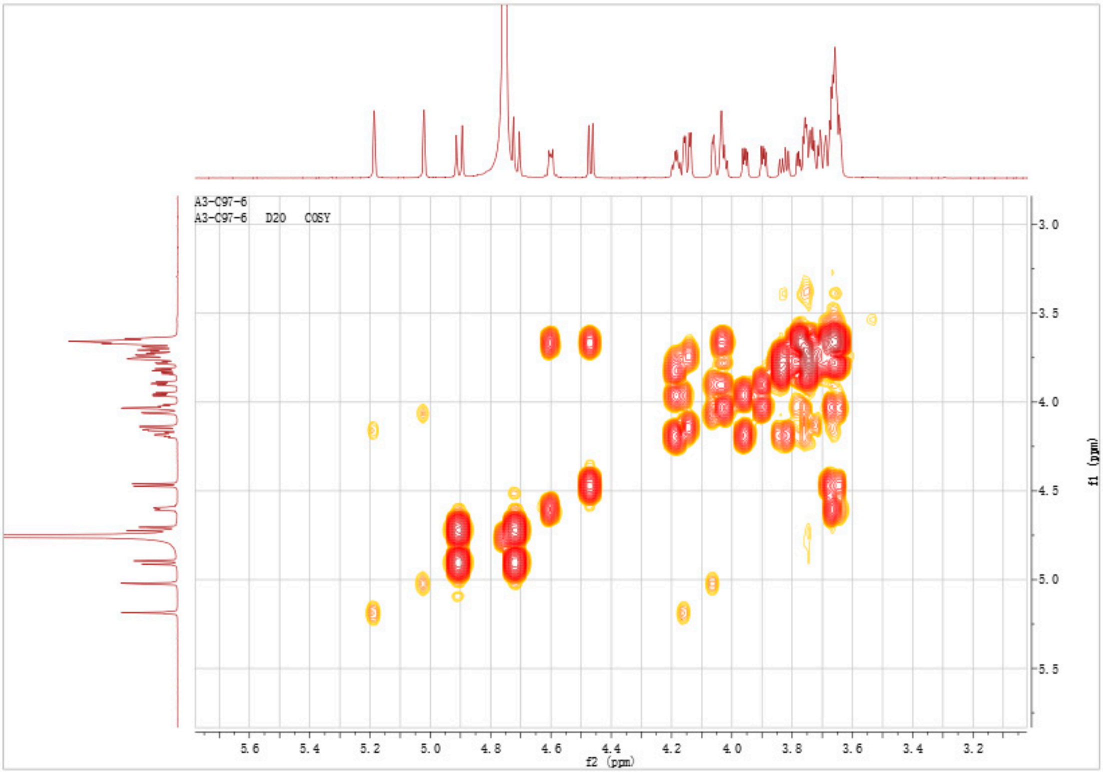

Figure S55. ^{1}H-^{1}H COSY of (CDCl3, 500 MHz) of 21

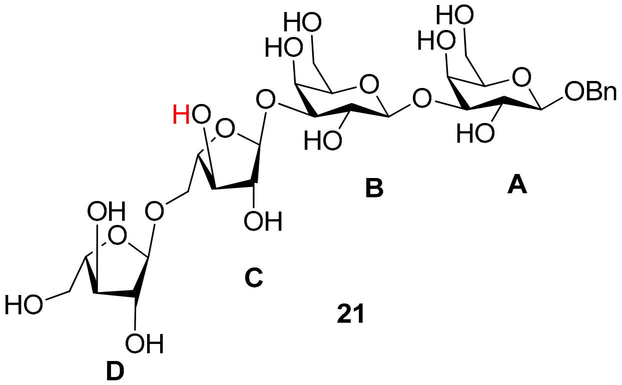

21

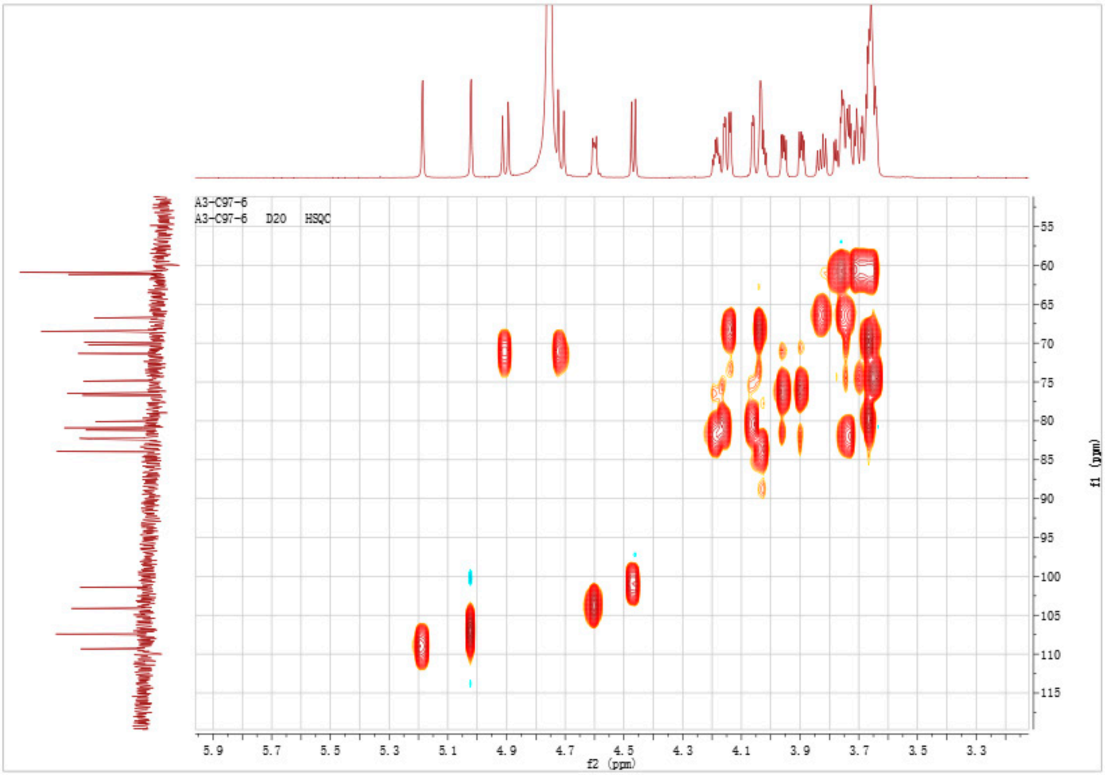

Figure S56. ^1H-^{13}C HSQC (CDCl3, 125 MHz) of 21

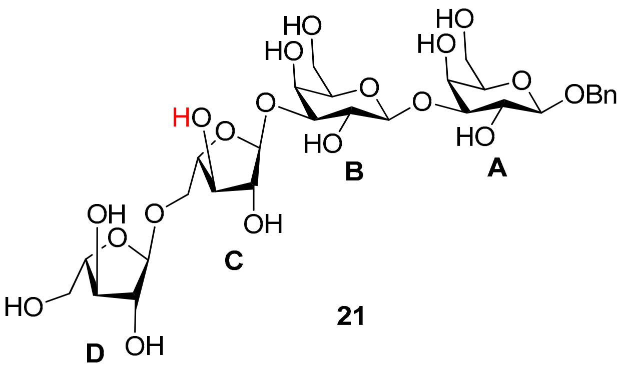

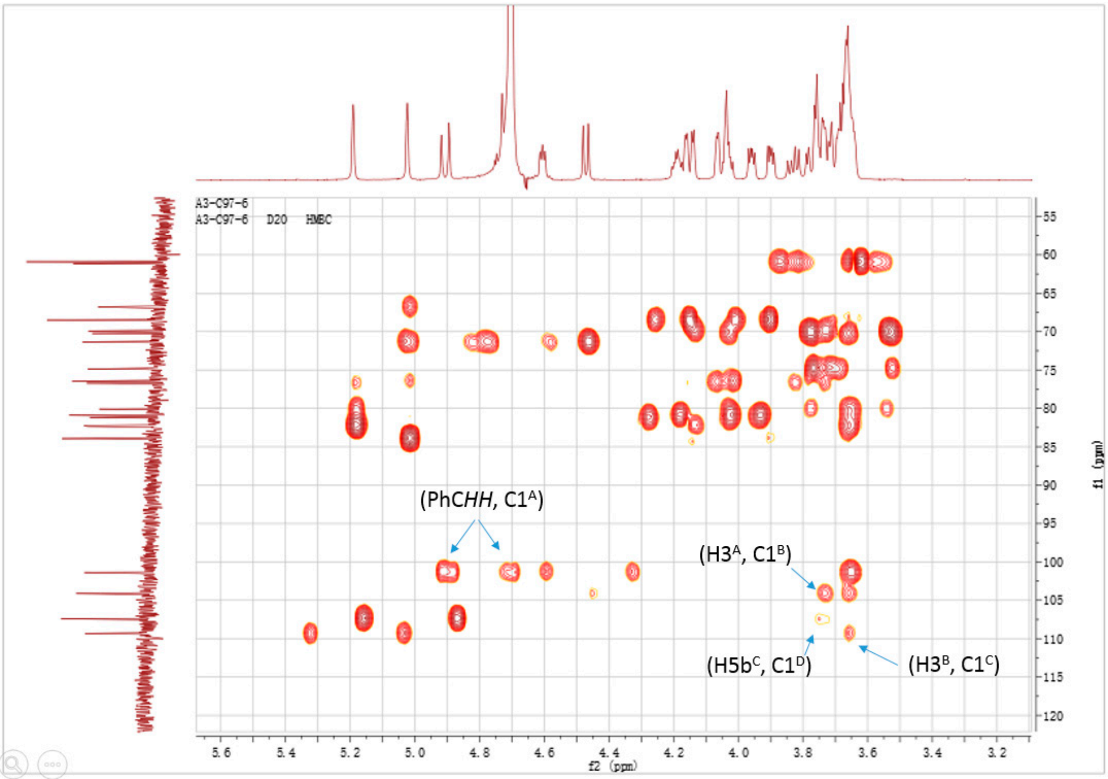

Figure S57. ^{1}H-^{13}C HMBC (CDCl3, 125 MHz) of 21

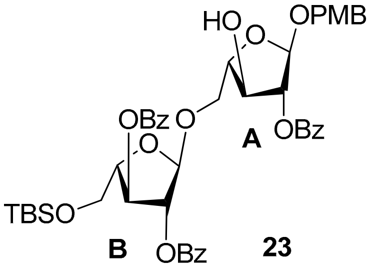

23

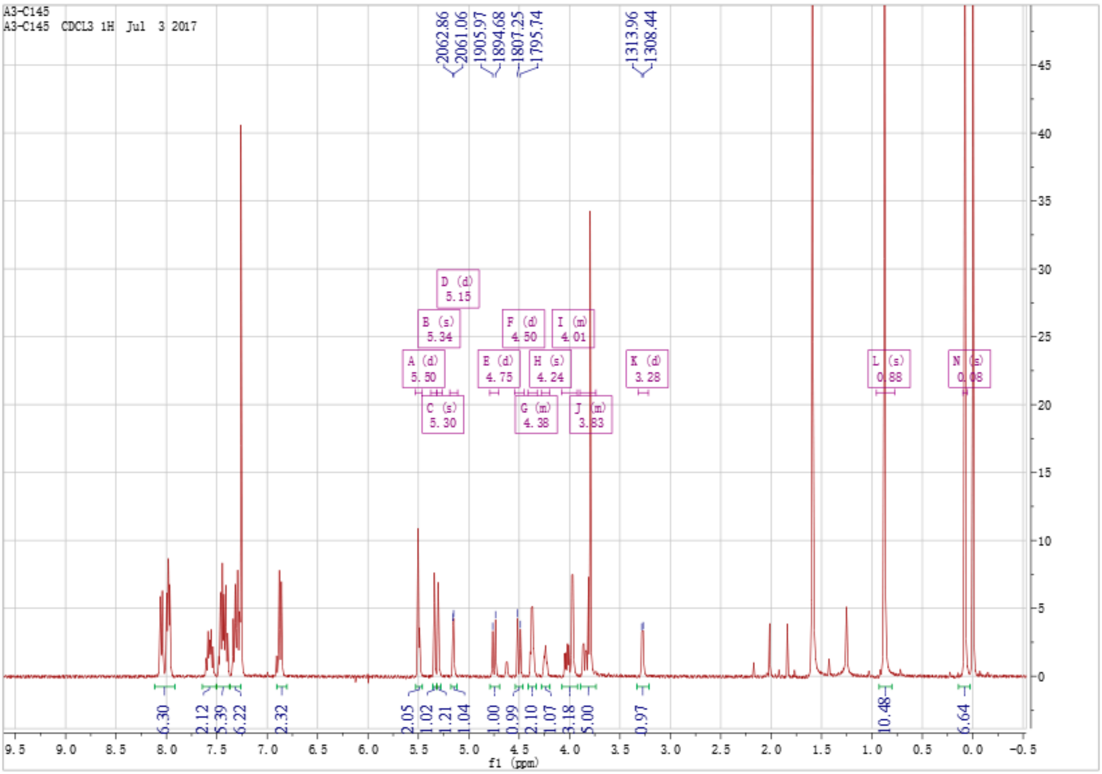

Figure S58. ^{1}H-NMR (CDCl3, 400 MHz) of 23

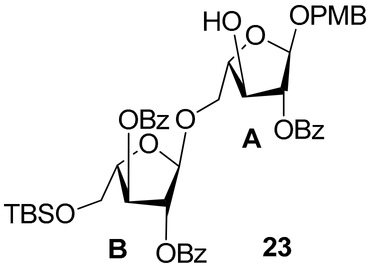

23

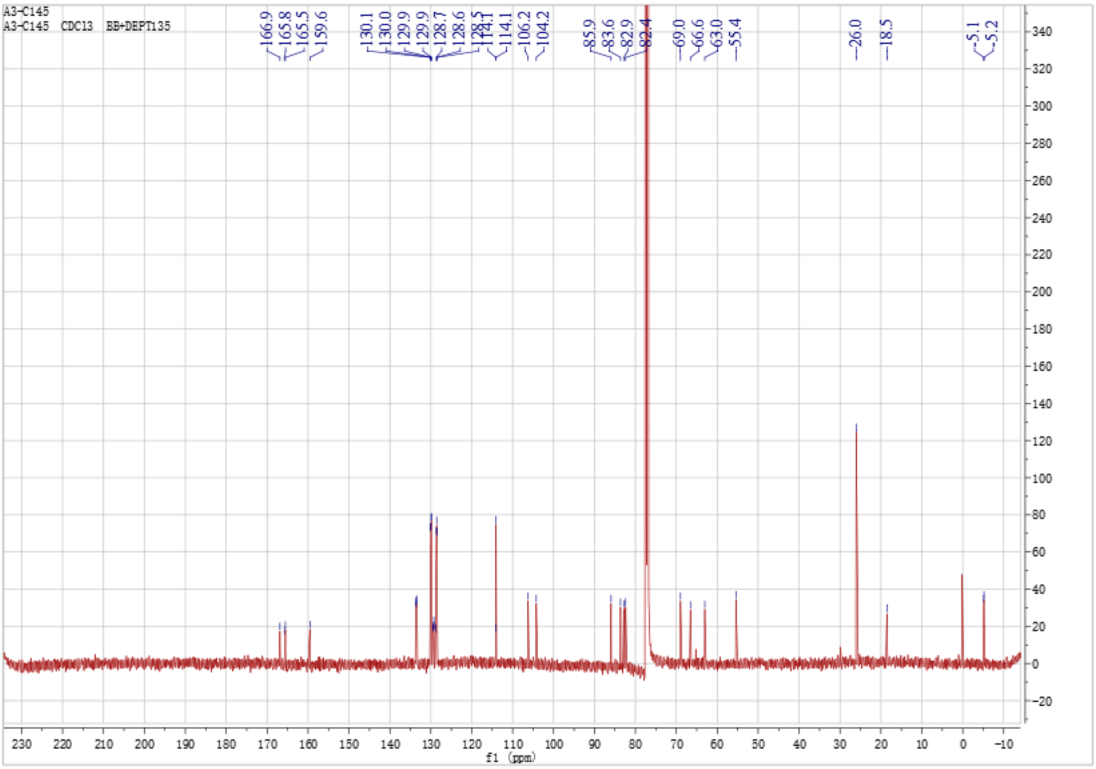

Figure S59. ^{13}C-NMR (CDCl3, 100 MHz) of 23

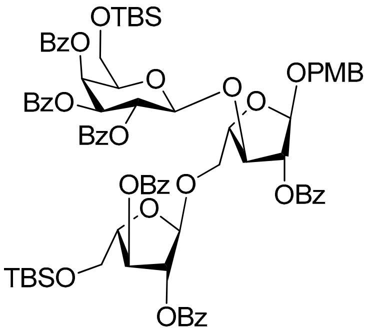

24

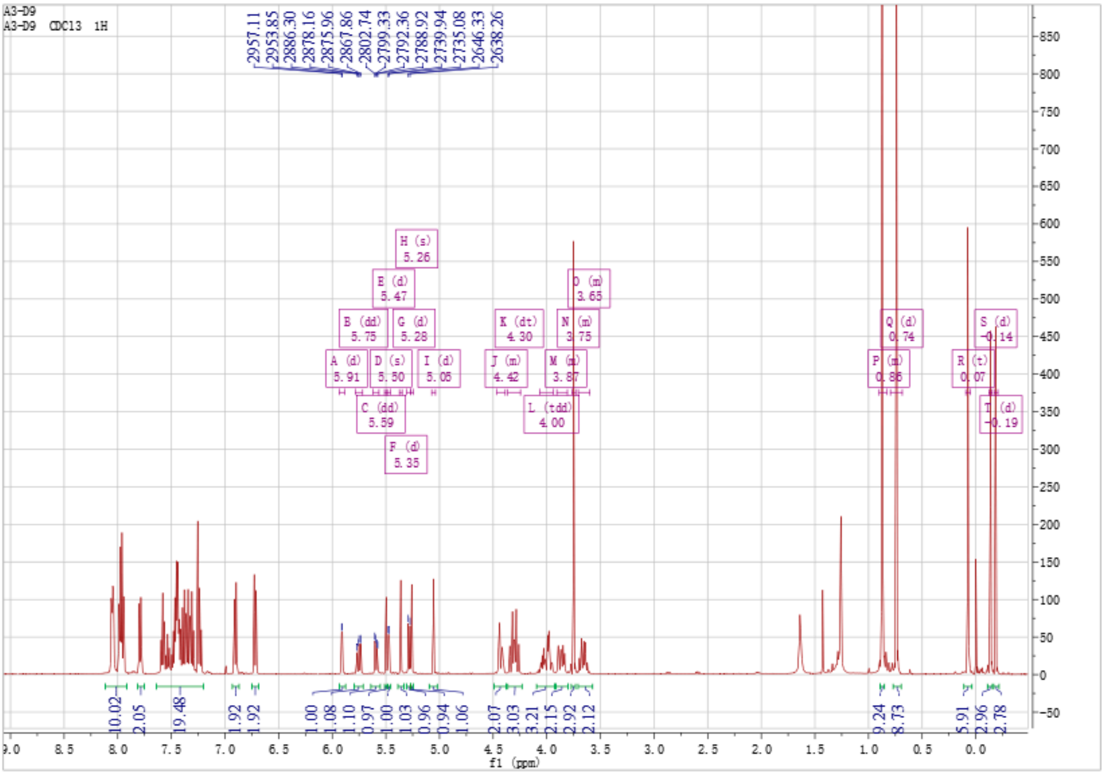

Figure S60. ^{1}H-NMR (CDCl3, 500 MHz) of 24

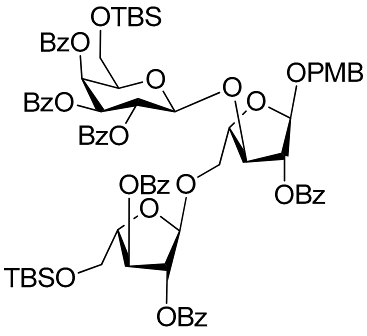

24

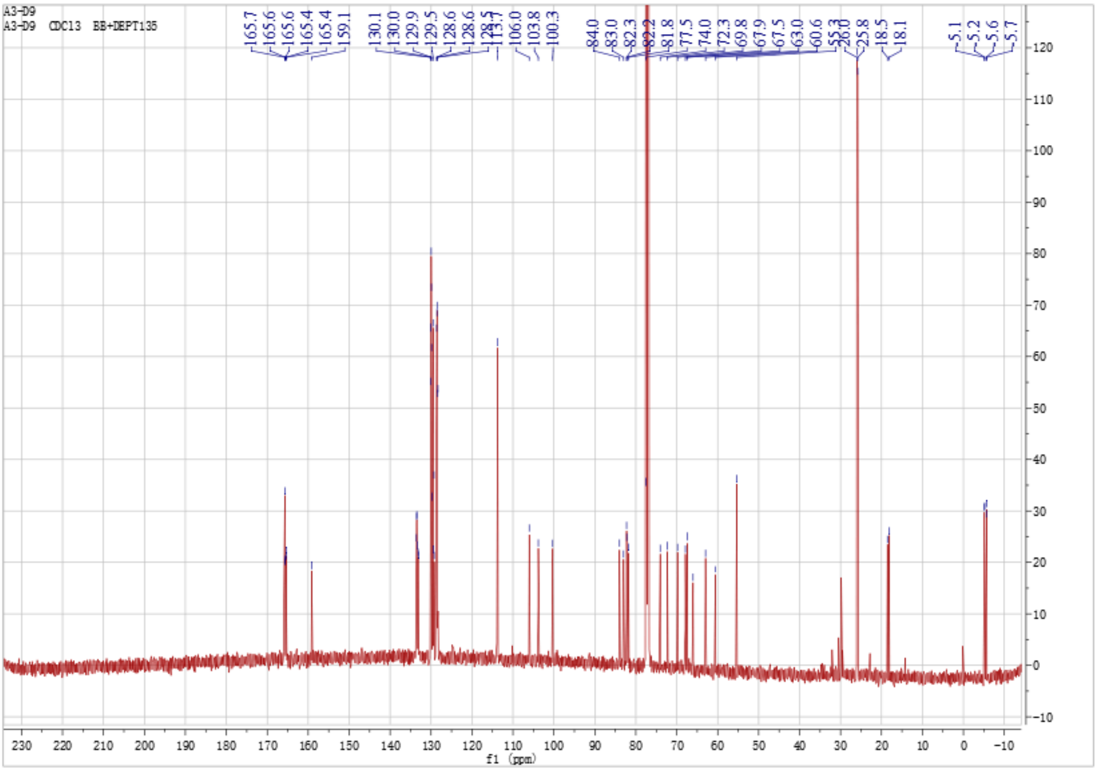

Figure S61. ^{13}C-NMR (CDCl3, 125 MHz) of 24

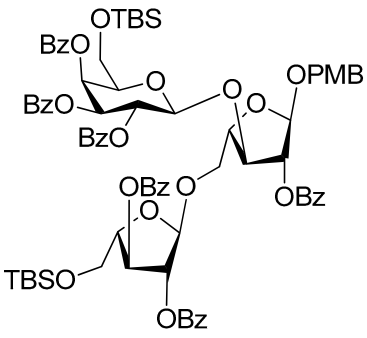

24

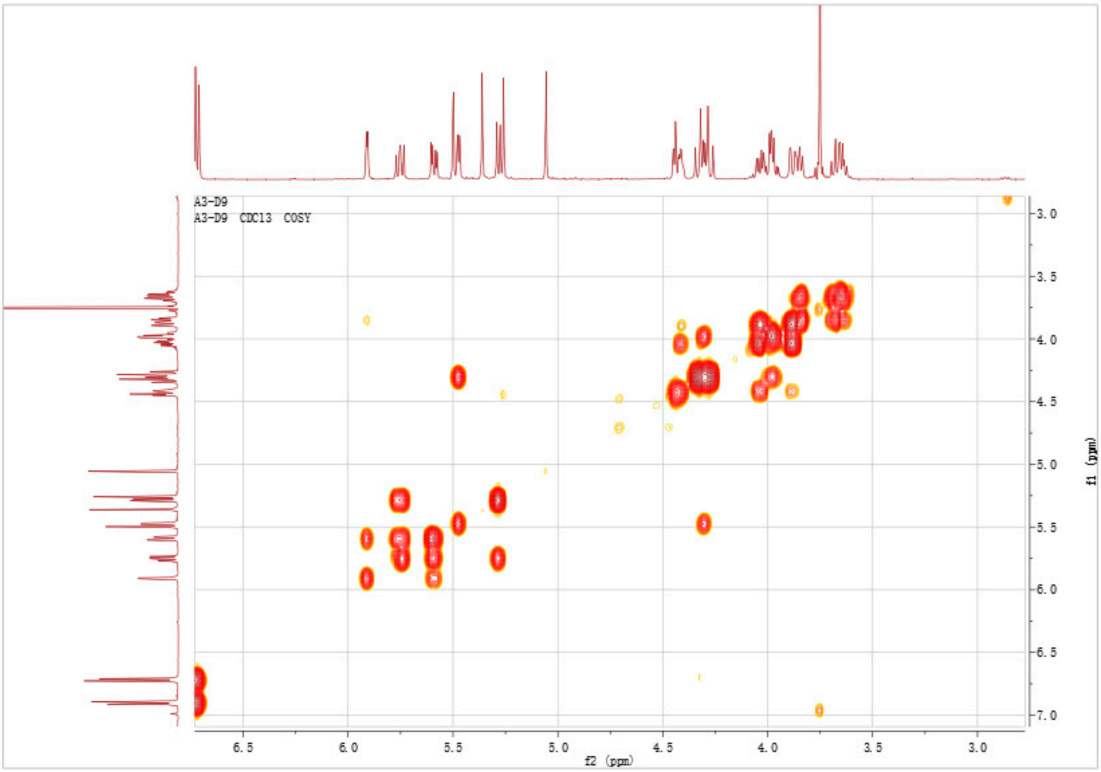

Figure S62. ^1H-^1H COSY of (CDCl3, 500 MHz) of 24

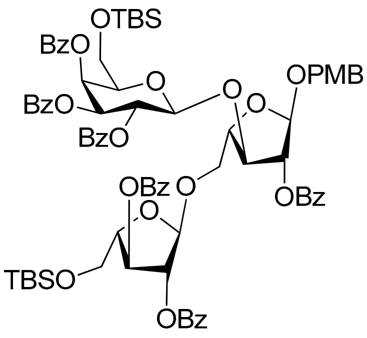

24

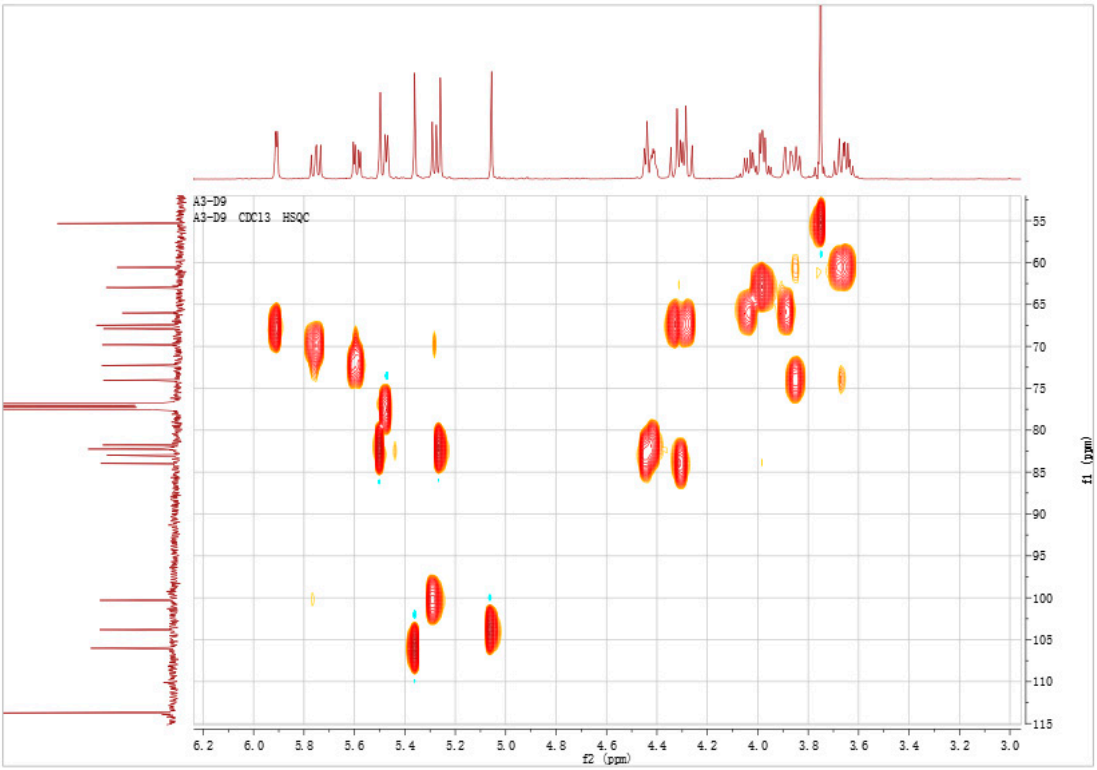

Figure S63. ^{1}H-^{13}C HSQC (CDCl3, 125 MHz) of 24

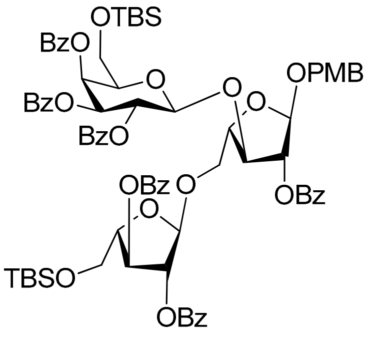

24

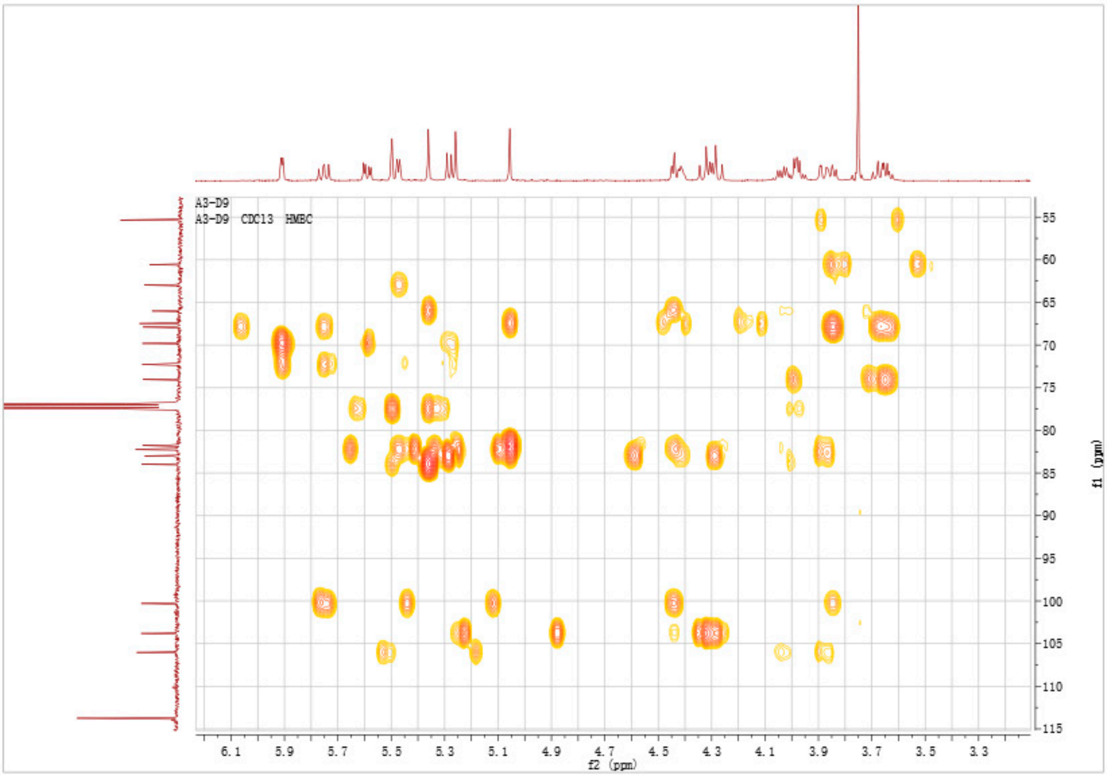

Figure S64. ^1H-^{13}C HMBC (CDCl3, 125 MHz) of 24

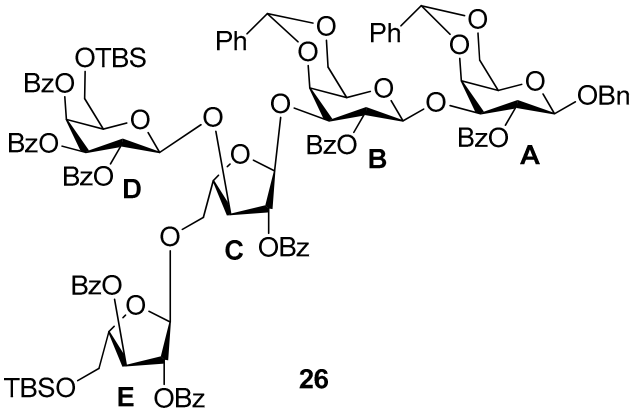

26

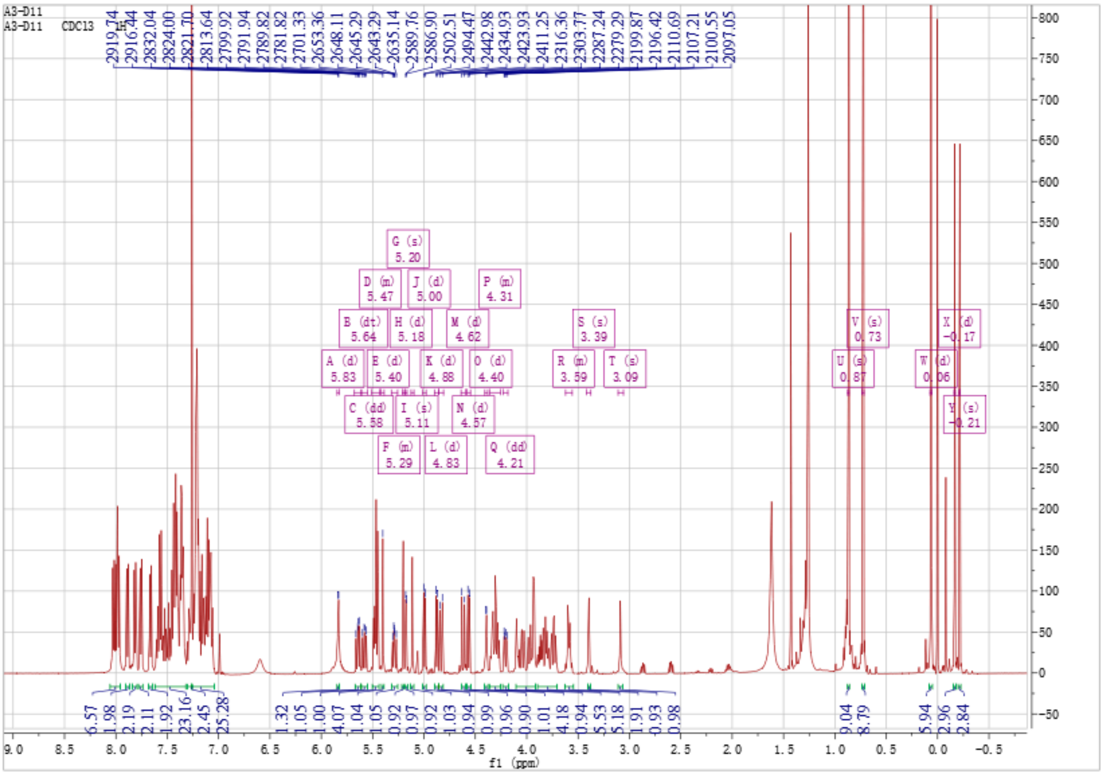

Figure S65. ^{1}H-NMR (CDCl3, 500 MHz) of 26

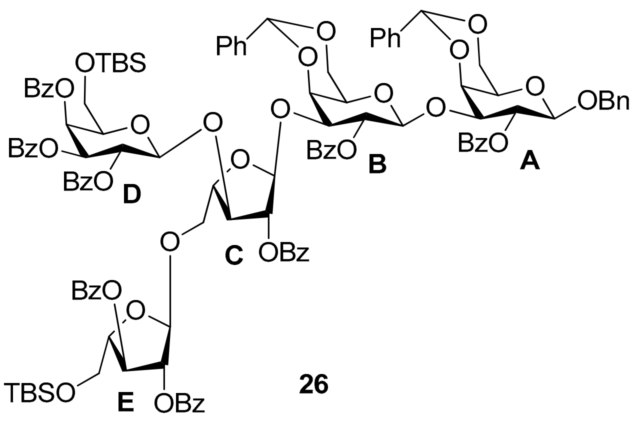

26

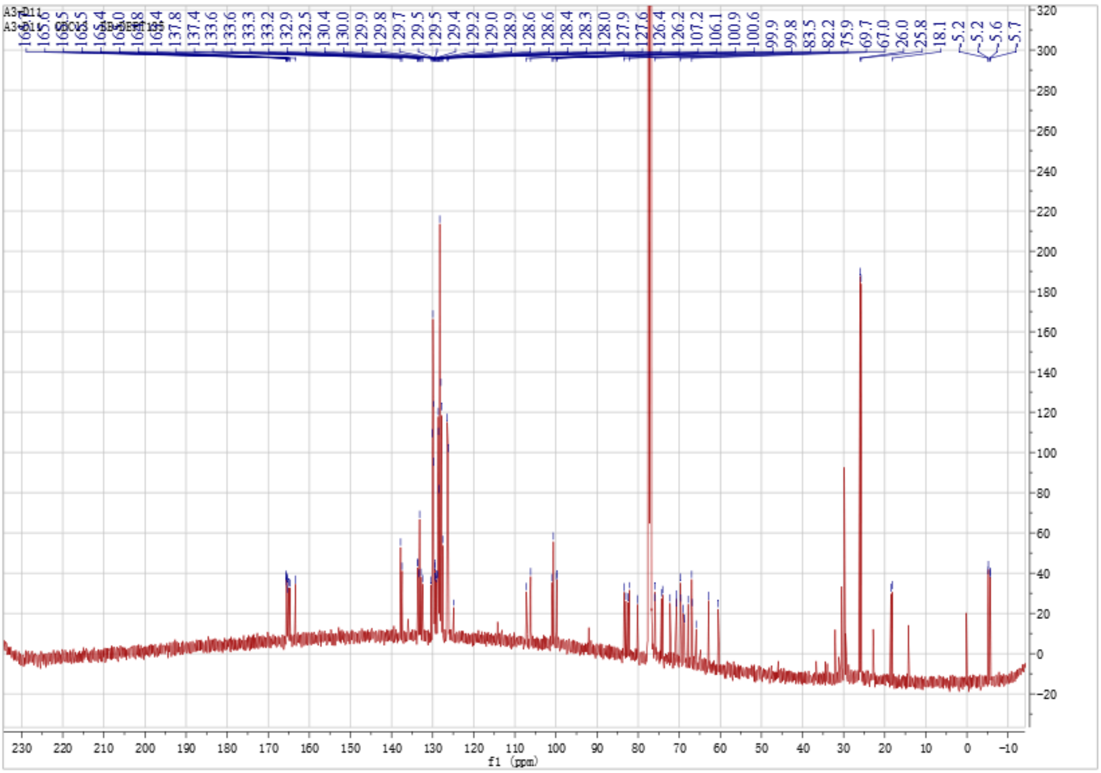

Figure S66. ^{13}C-NMR (CDCl3, 125 MHz) of 26

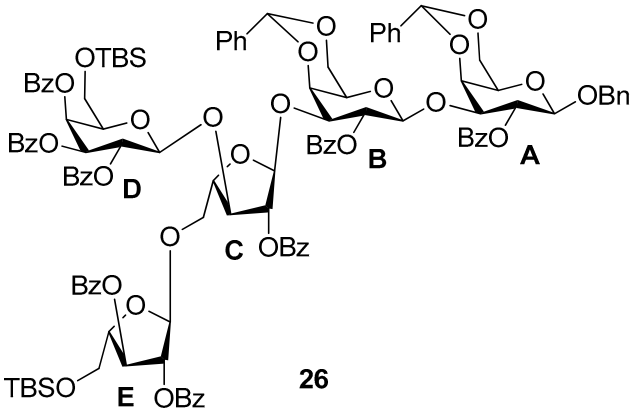

26

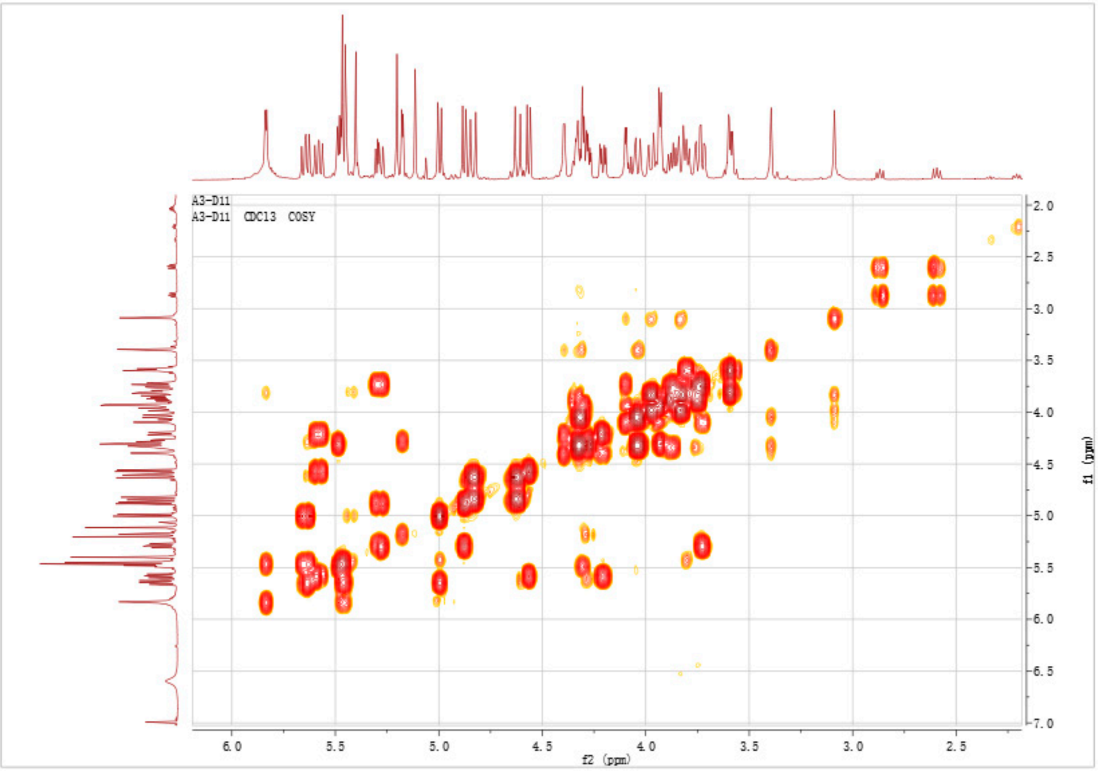

Figure S67. ^1H-^1H COSY of (CDCl3, 500 MHz) of 26

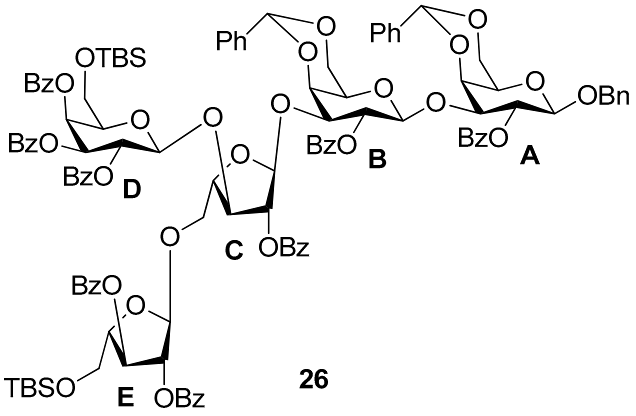

26

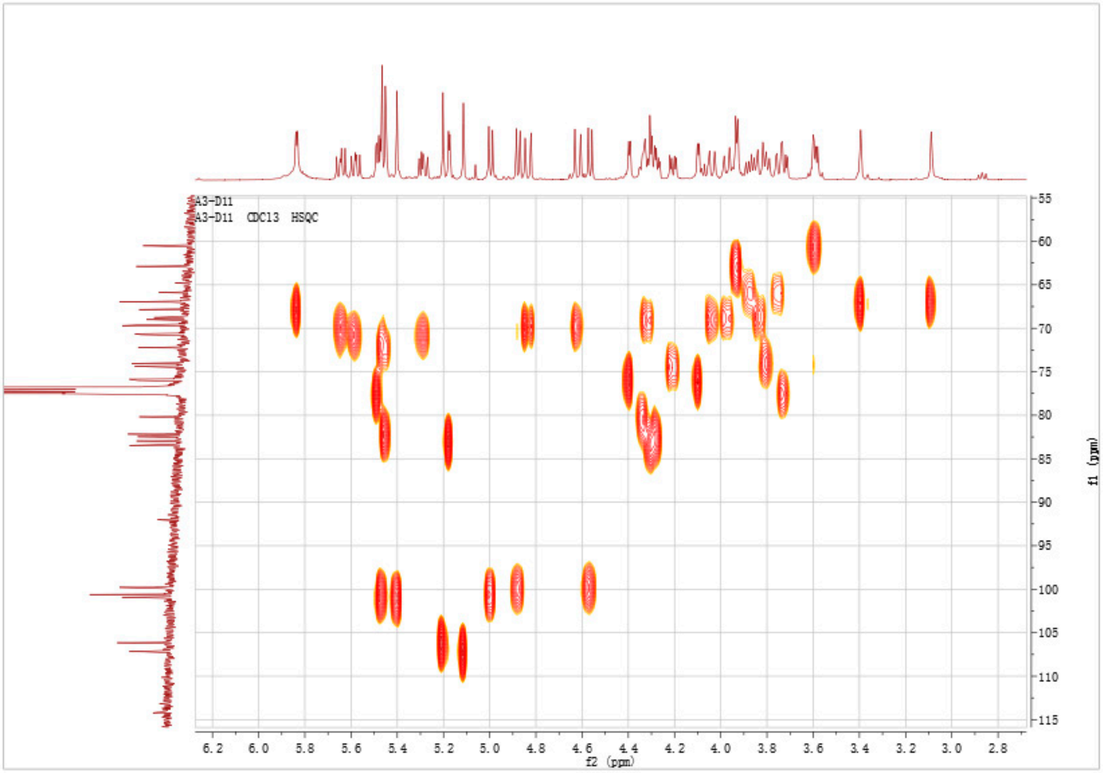

Figure S68. ^{1}H-^{13}C HSQC (CDCl3, 125 MHz) of 26

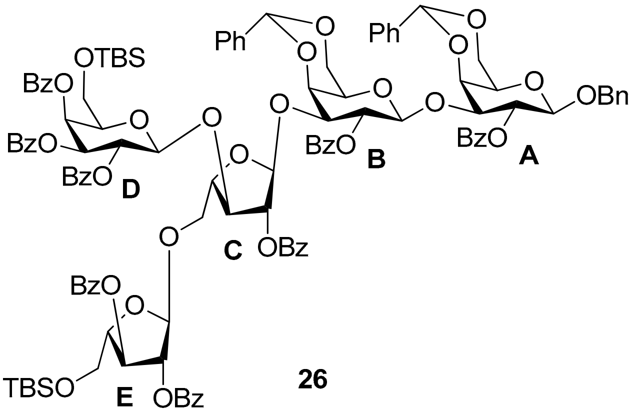

26

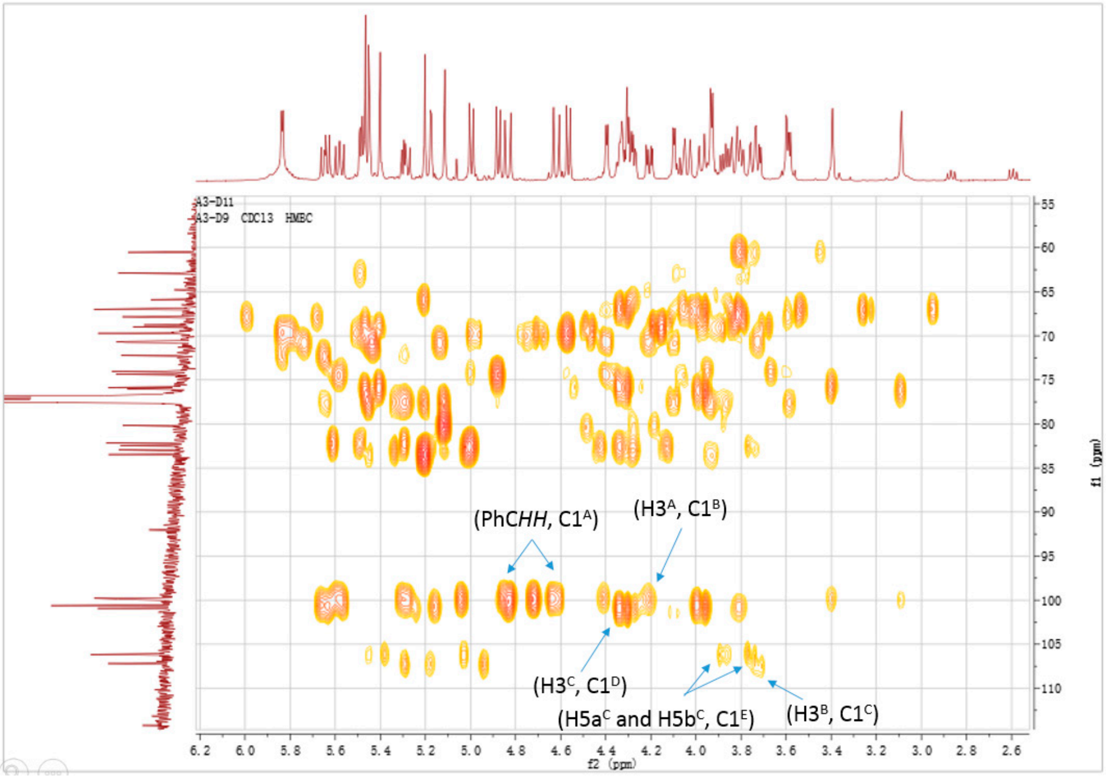

Figure S69. ^{1}H-^{13}C HMBC (CDCl3, 125 MHz) of 26

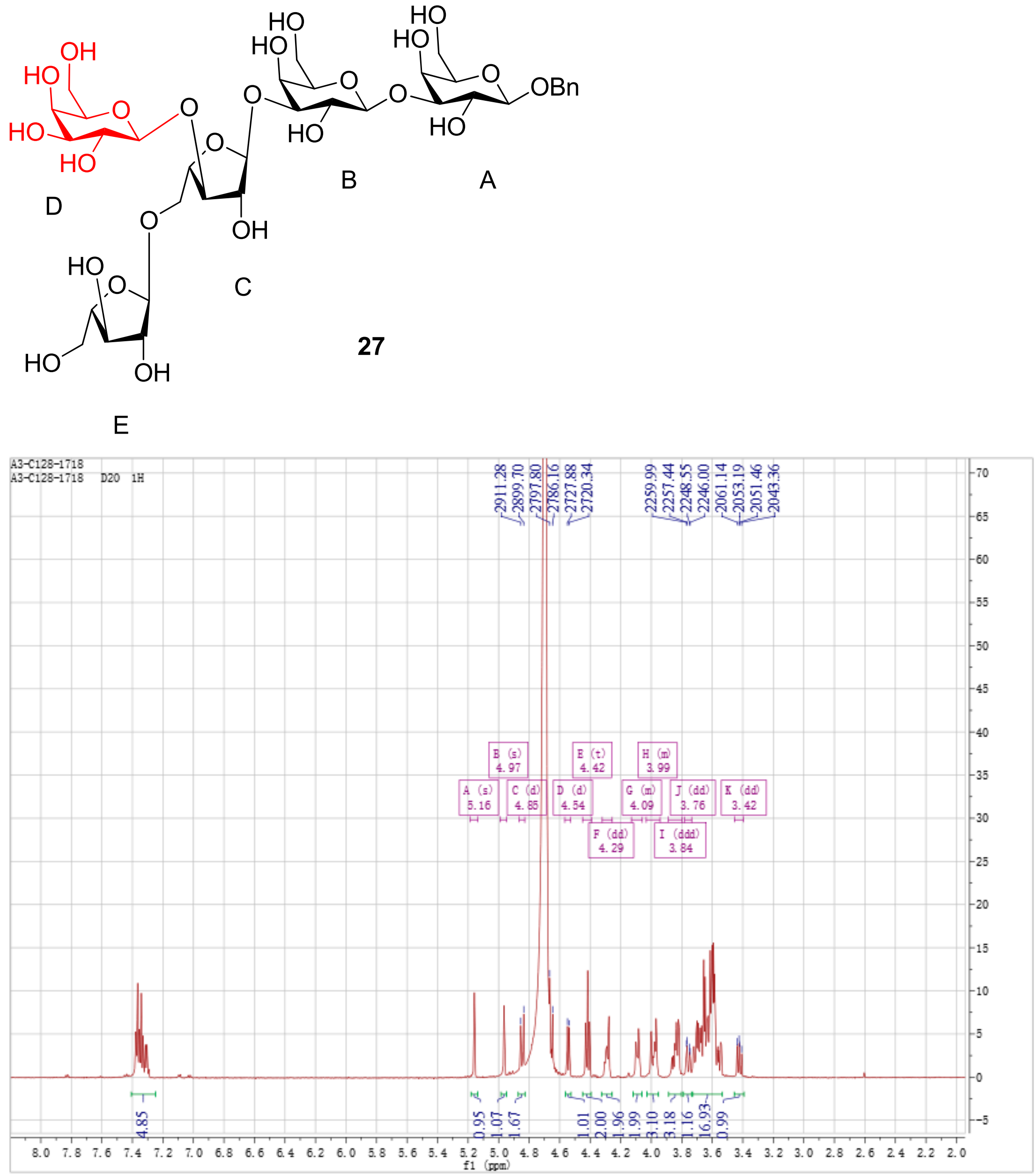

Figure S70. ^{1}H-NMR (CDCl3, 500 MHz) of 27

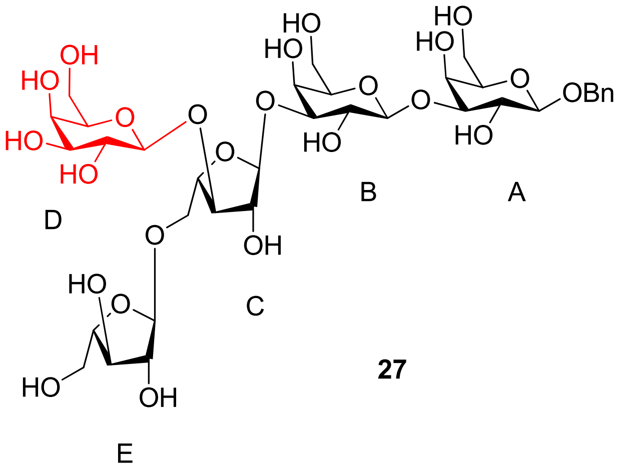

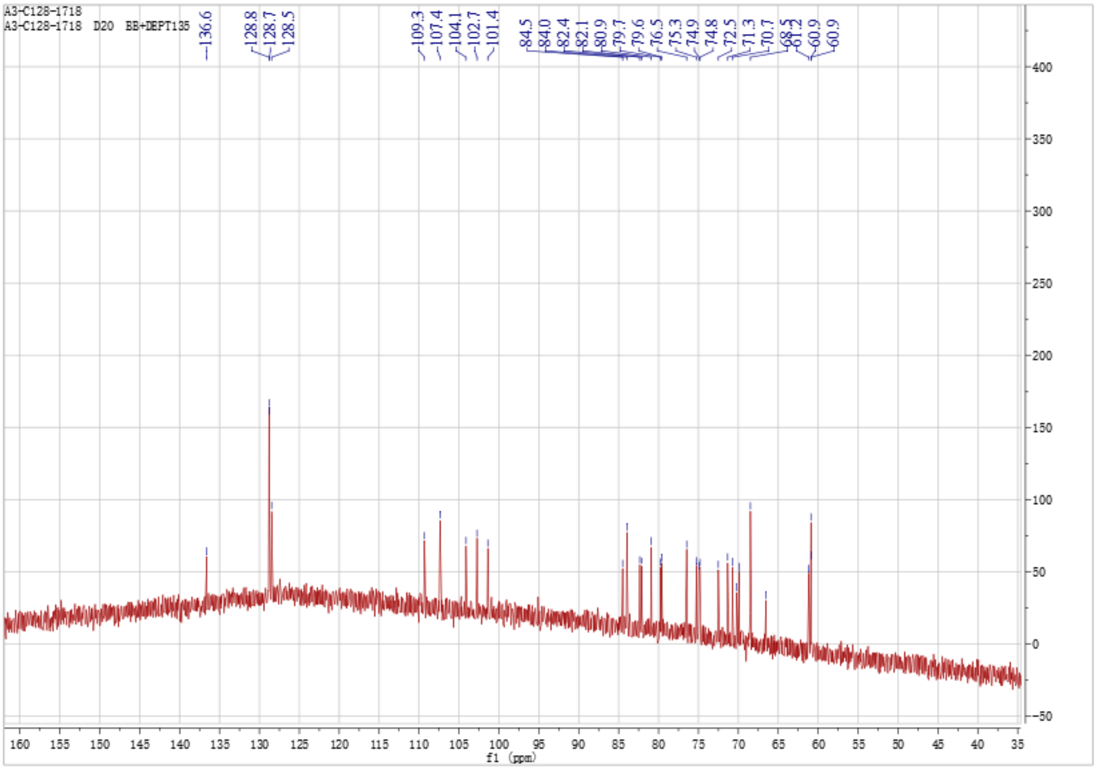

Figure S71. ^{13}C-NMR (CDCl3, 125 MHz) of 27

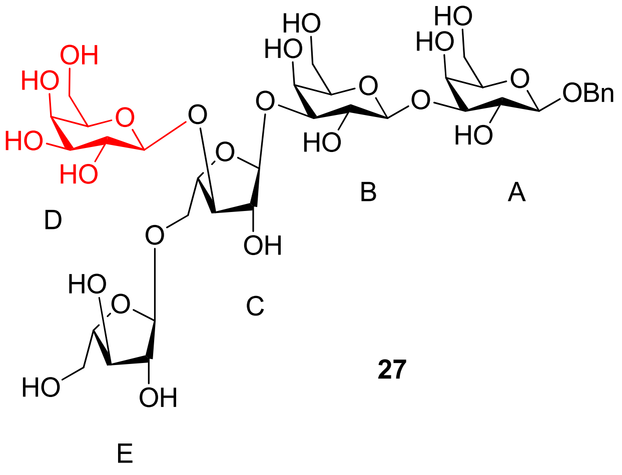

27

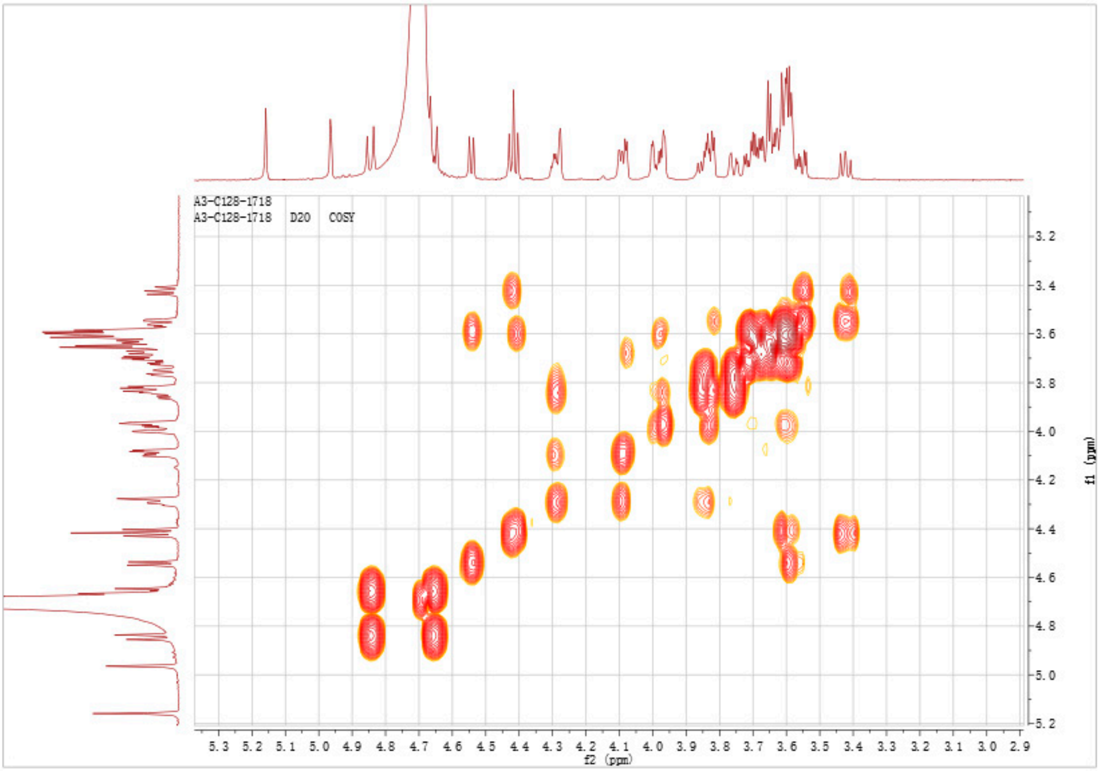

Figure S72. ^{1}H-^{1}H COSY of (CDCl3, 500 MHz) of 27

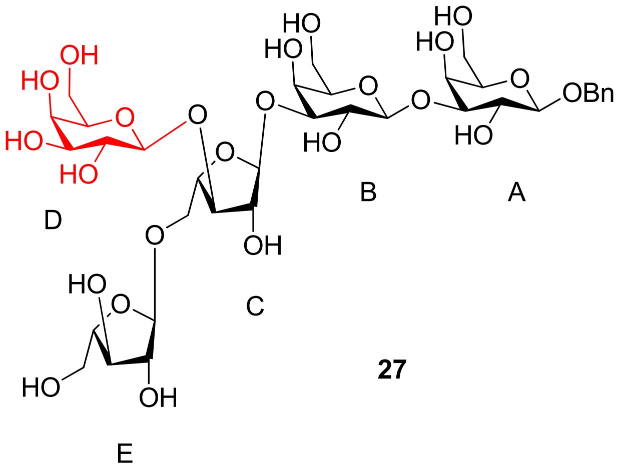

27

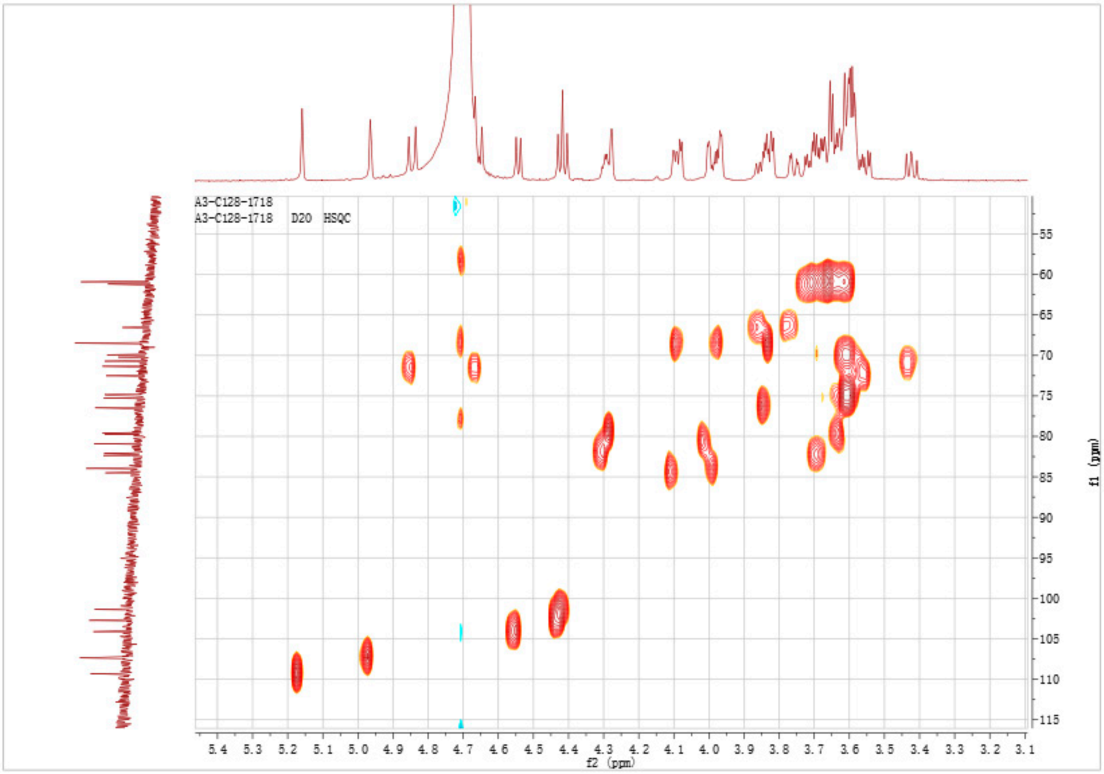

Figure S73. ^{1}H-^{13}C HSQC (CDCl3, 125 MHz) of 27

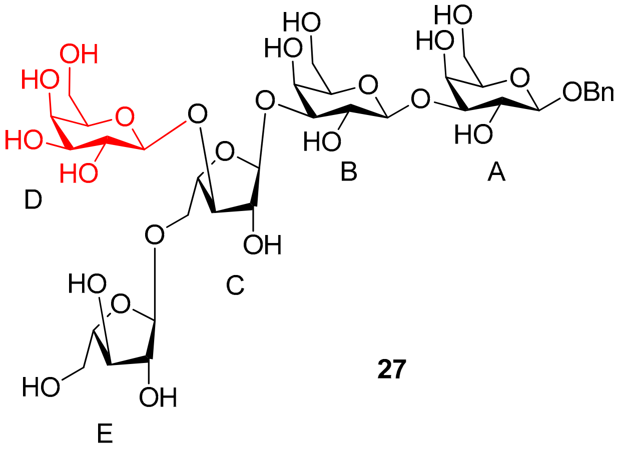

27

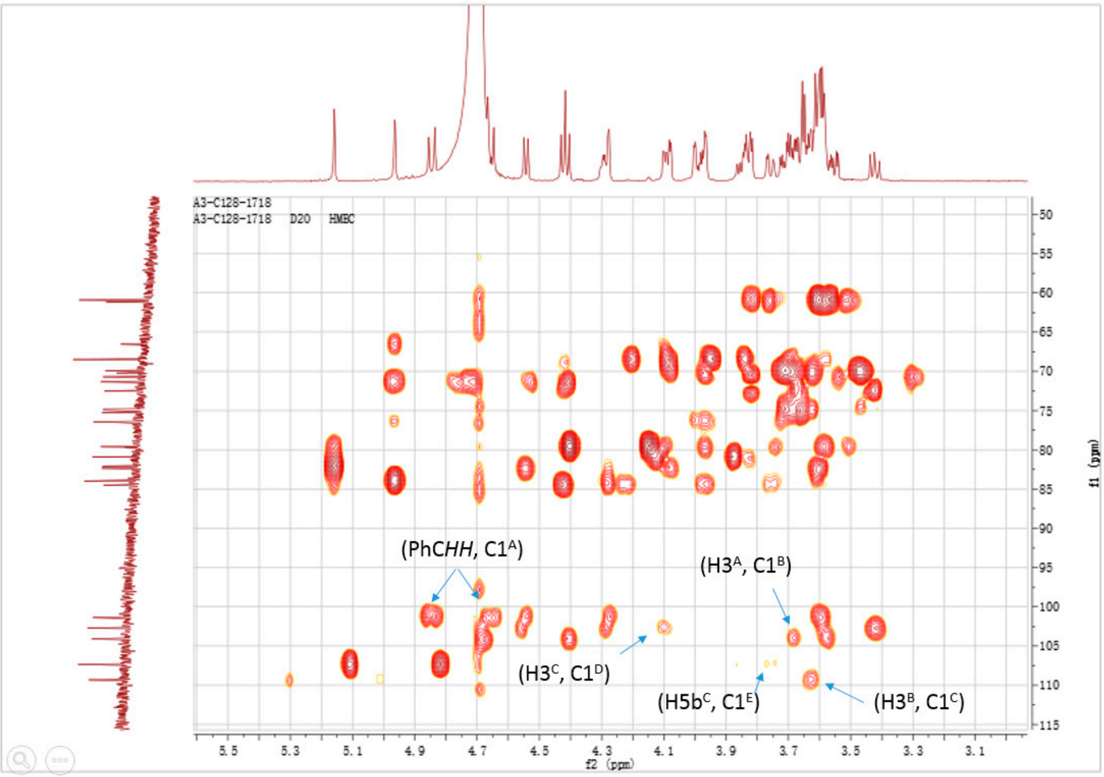

Figure S74. ^{1}H-^{13}C HMBC (CDCl3, 125 MHz) of 27

© 2017 by the authors. Submitted for possible open access publication under the terms and conditions of the Creative Commons Attribution (CC BY) license (http://creativecommons.org/licenses/by/4.0/).
